# Synergistic Carbon Encapsulation and Silver Decoration Enable Durable and Selective CO_2_-to-Ethylene Conversion on Copper Oxide Photoelectrode

**DOI:** 10.34133/research.1206

**Published:** 2026-03-16

**Authors:** Songying Qu, Ruiquan Yu, Min Gao, Jun Zhang

**Affiliations:** ^1^ Macao Institute of Materials Science and Engineering, Faculty of Innovation Engineering, Macau University of Science and Technology, Taipa 999078, Macao SAR.; ^2^ Institute of Environment and Ecology, Tsinghua Shenzhen International Graduate School, Tsinghua University, Shenzhen 518055, China.; ^3^ Advanced Interdisciplinary Institute of Environment and Ecology, Guangdong Provincial Key Laboratory of Wastewater Information Analysis and Early Warning, School of Technology for Sustainability, Beijing Normal University, Zhuhai 519087, China.

## Abstract

Chemical alterations in metal oxides during manipulation greatly diminish their potential in artificial photosynthesis. Clarifying and overcoming these changes is crucial for realizing the sustainable generation of solar fuels and chemicals. Here, employing multimodal operando techniques, we elucidated the degradation mechanism of copper oxide (CuO_x_) photocathodes under operational conditions, revealing an electron-mediated reductive photocorrosion pathway: Cu_2_O/CuO → Cu_2_O → Cu. These key findings led us to engineer an ultrathin carbon layer encapsulation strategy formed via electrodeposition-coupled self-assembly of carbon nanodots. This protective layer enables efficient photoelectron extraction and spatial isolation. The resulting CuO_x_@C exhibits gratifying durability with unaltered phases and steady photocurrent throughout extended operation exceeding 24 h. To enhance activity and selectivity toward ethylene, Ag nanoparticles were integrated onto CuO_x_@C. The Ag decoration enhances CO_2_ adsorption, stabilizes *CO intermediate, and facilitates the crucial *CO–*CO coupling. The Faraday efficiency for CO_2_-to-ethylene conversion on CuO_x_@C/Ag reaches up to ~66.4% and retains ~95% of its initial performance after prolonged use. This synergistic strategy of carbon encapsulation and metal decoration exhibits broad applicability, as validated by CuO_x_@C/Ru, CuO_x_@C/Pd, and BiVO_4_@C/Pt. Our work provides a universal design framework for efficient and durable photoelectrodes, accelerating their transition from laboratory prototypes to scalable technologies.

## Introduction

The consumption and combustion of fossil fuels have caused severe energy shortages and environmental pollution, posing a major challenge to sustainable development [1,2]. To address it, there is increasing focus on the conversion and storage of renewable solar energy, especially through the reduction of CO_2_ into clean fuels as alternatives to fossil fuels [3,4]. Photoelectrochemical platforms, which utilize semiconductor photoelectrodes to absorb solar energy and drive uphill chemical reactions, offer a promising solution [5,6]. Among various photoelectrocatalysts, metal oxide photoelectrodes stand out owing to their low cost, facile preparation, and being environment friendly, which are crucial for practical applications [7,8].

However, existing efficient metal oxide photoelectrodes often suffer from catastrophic degradation under operational conditions, markedly hindering the development of photoelectrochemical systems as a viable alternative for energy conversion and storage [9,10]. Take, for example, copper oxide (CuO_x_, a heterojunction of Cu_2_O/CuO) photocathodes, possessing a narrow band gap as well as a suitable band alignment, which can effectively harvest sunlight to drive CO_2_ reduction, hydrogen evolution, and nitrogen fixation, among others [11–13]. Unfortunately, CuO_x_ typically exhibits a sharp decay in activity and selectivity within a few minutes [4,14]. Despite the development of some schemes [15–17], up to now, the comprehension of the dynamic chemical transformations occurring in these materials, including CuO_x_, under light exposure remains extremely limited, despite being crucial for determining how to precisely improve their stability [18,19]. In this context, a rigorous understanding of the chemical transformation pathways of unstable photoelectrodes including CuO_x_ under operational conditions is imperative for designing rational protection strategies.

The most effective and convenient approach to overcoming the activity–stability trade-off is to deposit a conformal on unstable photoelectrodes [20,21]. Ideally, such a surface layer can promote carrier dynamics in addition to preventing degradation. However, the processes for constructing protective layers reported to date, such as the deposition of TiO_2_, graphene, g-C_3_N_4_, metal-organic frameworks, and polypyrrole, are often complex, costly, nonuniform, and uncontrollable [22–26]. Moreover, the protective efficacy of these clad layers is generally mediocre and far from satisfactory. There is thus an urgent need for new surface coating materials and methods to advance the practical application of metal oxide photoelectrodes in artificial photosynthesis. While a protective layer can promote charge migration and separation, thus enhancing photostability, it fails to provide the precise active sites necessary for high activity and selectivity in specific catalytic transformations. To address it, we propose the strategic integration of tailored cocatalysts (e.g., Ag, Ru, and Pd) onto the protection layer, forming an innovative sandwich architecture [27–29]. This rational design strategy offers a versatile route toward efficient and durable photoelectrochemical systems.

**Fig. 1.**
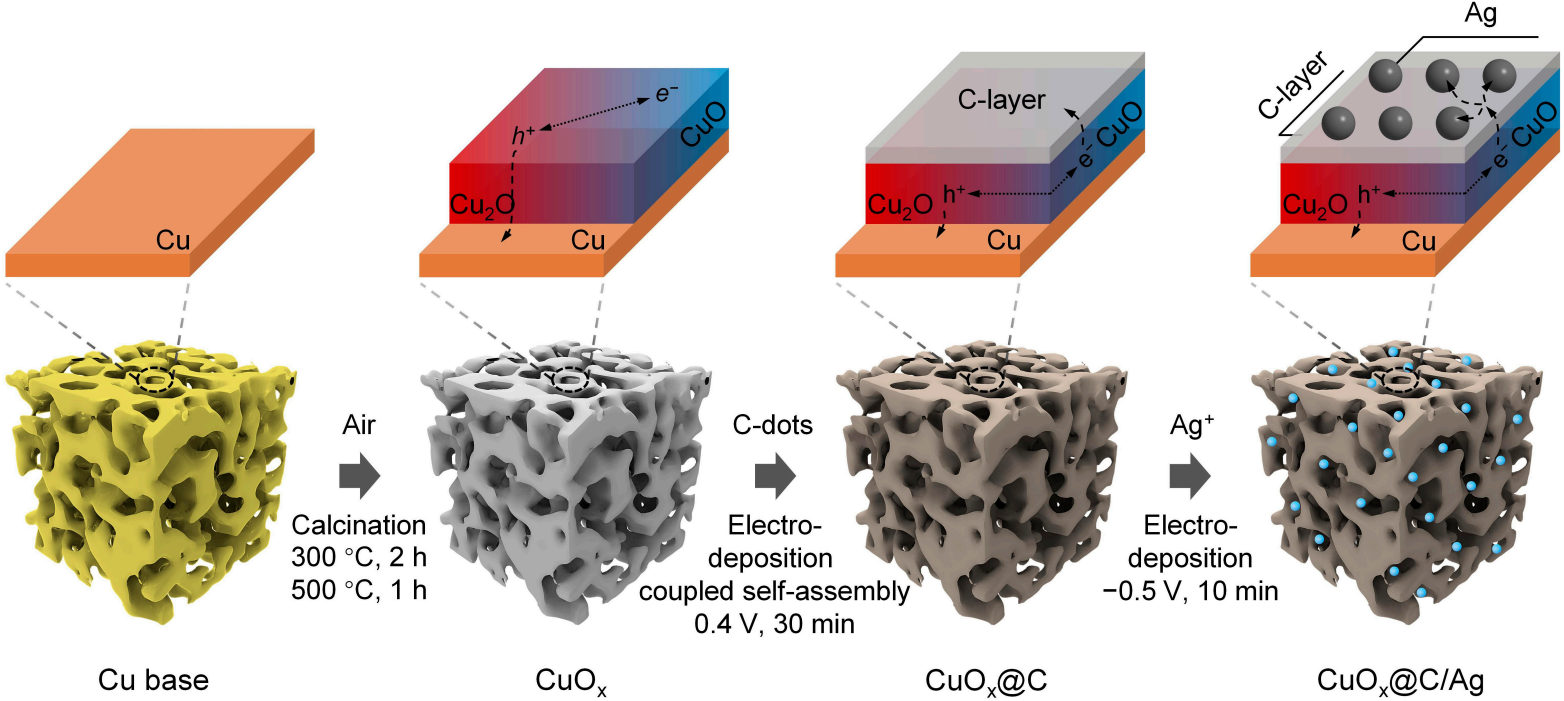
Schematic diagram of synthesis path of CuO_x_@C/Ag photoelectrodes.

Herein, we elucidated the photoelectrocatalytic transformation mechanisms of CuO_x_, as a model photoelectrode, using a correlative characterization approach. Under illumination, CuO_x_ undergoes electron-mediated reduction photocorrosion, summarized as: Cu_2_O/CuO → Cu_2_O → Cu. To mitigate this degradation, we innovatively proposed an ultrathin carbon layer encapsulation strategy formed via the electrodeposition-coupled self-assembly of carbon nanodots (Fig. 1 and Fig. S1). This protective layer effectively extracts photoelectrons, thereby achieving spatial isolation. Further functionalization of the resulting CuO_x_@C with Ag nanoparticles enhances the activity and selectivity of CO_2_ reduction to ethylene (C_2_H_4_), achieving a Faraday efficiency (FE) of ~66.4% and retaining ~95% of its initial performance after prolonged use. This approach also stabilizes photoanodes (e.g., BiVO_4_) extending their service life.

## Results and Discussion

### In situ visualization of CuO_x_ degradation

**Fig. 2.**
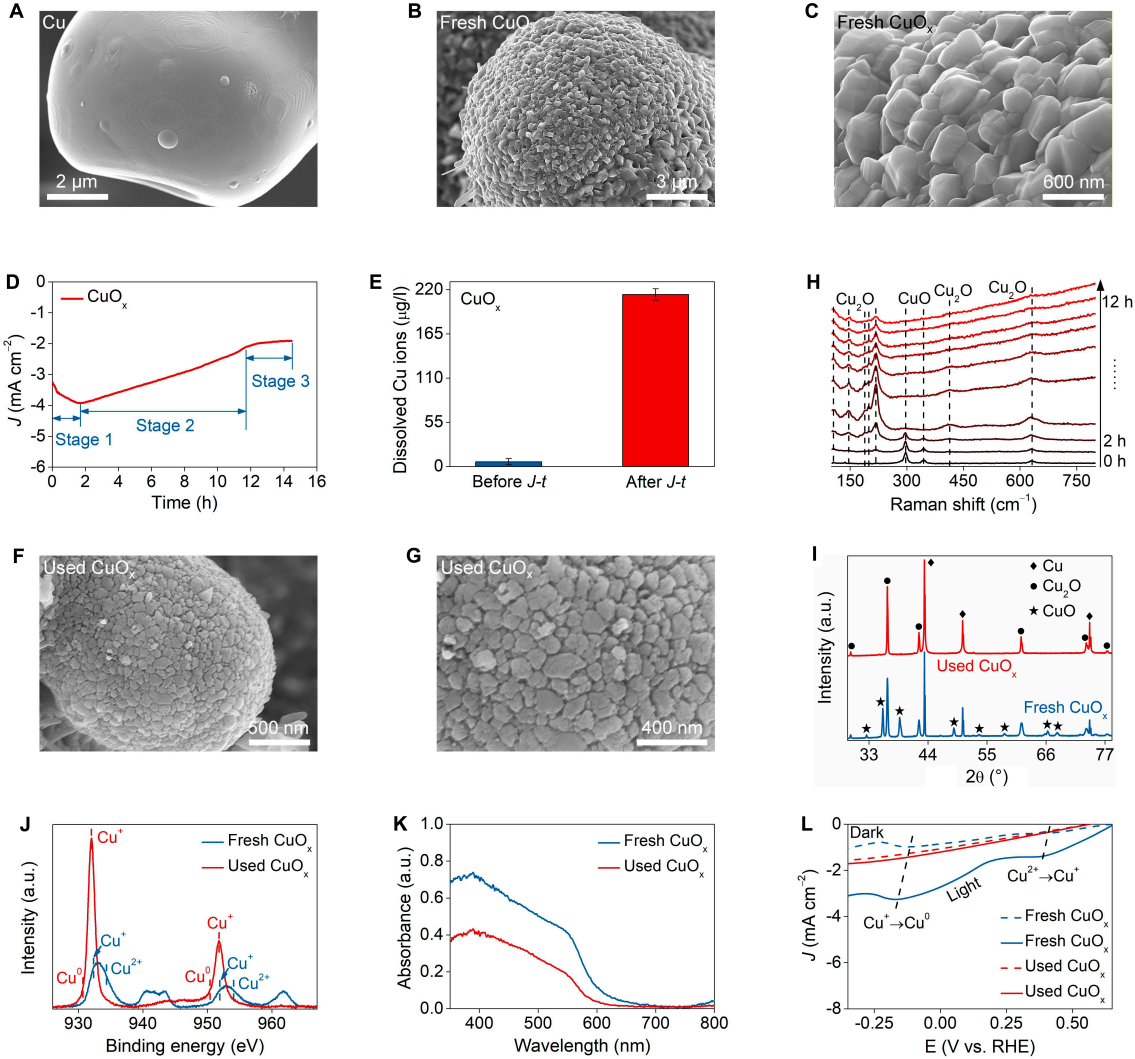
In situ visual investigation of CuO_x_ degradation. The scanning electron microscope (SEM) images of (A) Cu base and (B and C) fresh CuO_x_. (D) The *J*–*t* plot of CuO_x_. (E) The released copper ions from CuO_x_. (F and G) The SEM images of used CuO_x_. (H) The in situ Raman spectra of CuO_x_. The (I) x-ray diffraction (XRD) patterns, (J) x-ray photoelectron spectroscopy (XPS) spectra, (K) diffuse reflection spectroscopy (DRS) spectra, and (L) *J*–*V* plots of fresh and used CuO_x_. Experimental conditions: at −0.1 V vs. reversible hydrogen electrode (RHE) under AM 1.5G simulated sunlight (100 mW/cm^2^) using 0.1 M KHCO_3_ as electrolyte (CO_2_-saturated). Data are presented as mean ± SD (*n* = 3 independent chemical replicates) in (D).

The degradation process and mechanism of CuO_x_ photoelectrodes were investigated using several in situ characterization methods. Scanning electron microscope (SEM) images showed that the pristine Cu base surface was smooth and clean, free of any impurities (Fig. 2A and Fig. S2). By contrast, the CuO_x_ surface became rough due to the formation of Cu_2_O/CuO crystal (Fig. 2B). Irregularly shaped Cu_2_O/CuO particles, with an average size of ~500 nm, were evenly embedded on the Cu base surface (Fig. 2C and Fig. S3). When CuO_x_ was subjected to 15 h of long-term photoelectrocatalytic CO_2_ reduction operation, the photocurrent decayed severely and exhibited 3 distinct stages of change (Fig. 2D). In the first 2 h, the photocurrent dropped from ~−3.2 to ~−4.0 mA/cm^2^ (Stage 1). Then, the photocurrent reversed and rose to ~−2.1 mA/cm^2^ after the intermediate 10 h (Stage 2) and finally stabilized at ~−1.7 mA/cm^2^ after the last 3 h (Stage 3). Meanwhile, copper ions continuously leached from CuO_x_ during the operation, and the leaching rate was slow at first, became fast, and went slow again, which corresponded to the photocurrent changes (Fig. S4). The cumulative amount of copper ions leached reached up to ~212 μg/L at the end of the reaction (Fig. 2E).

The fresh CuO_x_ was black in appearance but turned rufous after use, indicating an obvious change in morphology and structure (Fig. S5). SEM images showed that the used CuO_x_ surface became relatively flat, and the Cu_2_O/CuO crystal particles became obscured in contour and amorphous, confirming the degradation of morphology and structure (Fig. 2F and G and Fig. S6). In situ Raman spectra indicated that the peaks of CuO declined heavily in Stage 1 and almost disappeared by the end (Fig. 2H). In contrast, the peaks of Cu_2_O were markedly enhanced in Stage 1, and then gradually weakened until they became blurred. The results demonstrated the successive degradation of CuO and Cu_2_O. X-ray diffraction (XRD) patterns further proved that the CuO phase had completely degraded in used CuO_x_ (Fig. 2I). Based on the x-ray photoelectron spectroscopy (XPS) of the Cu 2p spectrum of used CuO_x_, the peaks of Cu^2+^ disappeared, while the peaks of Cu^+^ were markedly enhanced (Fig. 2J). Meanwhile, the characteristic peaks of Cu^0^ appeared in used CuO_x_. XPS O 1s spectra showed that the peak corresponding to Cu–O bonds was severely reduced in used CuO_x_ (Fig. S7). However, a new peak corresponding to Cu^2+^–OH species appeared [4].

The above analyses revealed that most of the CuO phase in CuO_x_ was reduced to Cu_2_O phase by photoelectrons in Stage 1, leading to a remarkable increase in the Cu_2_O proportion (Fig. S8). The intrinsic activity of Cu_2_O was better than that of CuO; thus, the absolute value of photocurrent increased slightly. This phase transition released a small amount of copper ions due to the loss of half of lattice oxygen. In Stage 2, most of Cu_2_O sharply decayed to Cu, resulting in rapid photocurrent attenuation. This phase transition released a large amount of copper ions due to the loss of almost all lattice oxygen. In Stage 3, the remaining CuO and Cu_2_O further degraded, resulting in continuous but gradual photocurrent reduction and copper ion release. The degradation process can be summarized as: Cu_2_O/CuO → Cu_2_O → Cu. Such electron-mediated severe photocorrosion led to an obvious decrease in the light capture performance and photoelectric conversion efficiency of used CuO_x_ (Fig. 2K and Fig. S9). Although the formation of Cu reduced the impedance and increased conductivity, the disappearance of active components resulted in disastrous photocurrent decay (Fig. 2L and Fig. S10).

### In situ visualization of CuO_x_@C durability

**Fig. 3.**
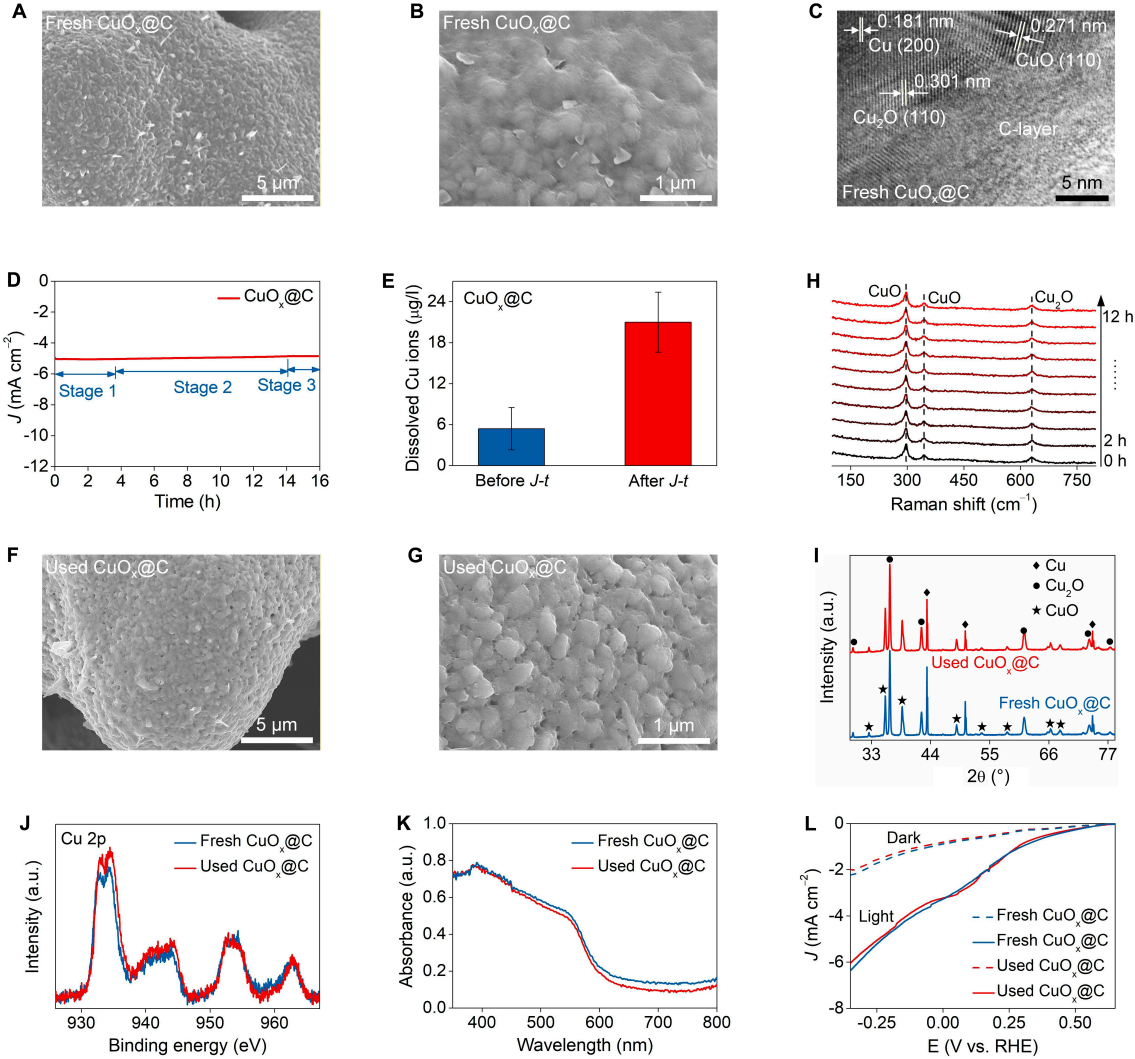
In situ visual investigation of CuO_x_@C durability. The (A and B) SEM and (C) transmission electron microscope (TEM) images of fresh CuO_x_@C. (D) The *J*–*t* plot of CuO_x_@C. (E) The released copper ions from CuO_x_@C. (F and G) The SEM images of used CuO_x_@C. (H) The in situ Raman spectra of CuO_x_@C. The (I) XRD patterns, (J) XPS spectra, (K) DRS spectra, and (L) *J*–*V* plots of fresh and used CuO_x_@C. Experimental conditions: at −0.1 V vs. RHE under AM 1.5G simulated sunlight (100 mW/cm^2^) using 0.1 M KHCO_3_ as electrolyte (CO_2_-saturated). Data are presented as mean ± SD (*n* = 3 independent chemical replicates) in (D).

Next, we investigated the durability of CuO_x_@C photoelectrodes using the same methods. SEM images showed that CuO_x_ was fully encapsulated in a carbon layer (Fig. 3A and B and Fig. S11). Transmission electron microscope (TEM) images illustrated that coating was extremely uniform, with an average thickness of ~6 nm, confirming that the carbon layer was formed by the self-assembly of single-row carbon nanodots (Fig. 3C and Figs. S12 and S13). When CuO_x_@C was subjected to 15 h of long-term photoelectrocatalytic CO_2_ reduction operation, the photocurrent remained almost constant (Fig. 3D). The absolute value of the photocurrent only decreased slightly from the initial ~4.8 mA/cm^2^ to the final ~4.7 mA/cm^2^. It is worth noting that the carbon layer encapsulation increased the onset photocurrent by ~30%. Additionally, there was extremely low copper ion leaching from CuO_x_@C during the long-term operation, and the amount of copper ions leached did not exceed ~20 μg/L at the end of reaction (Fig. 3E and Fig. S14).

CuO_x_@C was black in appearance, and its color hardly changed after use, indicating negligible changes in morphology and structure (Fig. S15). SEM and TEM images showed that used CuO_x_@C surface was almost identical to fresh CuO_x_@C, with the carbon layer (thickness ~6 nm) uniformly covering the CuO_x_ surface (Fig. 3F and G and Figs. S16 and S17). The atomic ratios of Cu, O, and C in CuO_x_@C remained unchanged before and after use, further confirming the structural stability. In situ Raman spectra indicated that the peaks of CuO and Cu_2_O in CuO_x_@C remained unchanged throughout the long-term operation (Fig. 3H and Fig. S18). XRD patterns further proved that neither CuO phase nor Cu_2_O phase degraded during use (Fig. 3I). In the XPS spectra of Cu 2p, O 1s, and C 1s for CuO_x_@C, the positions and intensities of all peaks remained largely unchanged before and after use (Fig. 3J and Figs. S19 and S20).

The above analyses revealed that the carbon layer encapsulation effectively mitigated CuO_x_ degradation, endowing CuO_x_@C with gratifying durability. The CuO phase and Cu_2_O phase remained stable throughout all stages of the long-term operation, resulting in a steady photocurrent with only slight attenuation (Fig. S21). Meanwhile, there was extremely low copper ion release due to the absence of obvious phase transition and lattice oxygen loss. This outstanding stability resulted in negligible changes in the light capture performance, photoelectric conversion efficiency, and electrochemical impedance of used CuO_x_@C (Fig. 3K and Figs. S22 and S23). Linear sweep voltammetry (LSV) curves of CuO_x_ exhibited 2 reduction peaks, corresponding to the processes of Cu^2+^ → Cu^1+^ and Cu^1+^ → Cu^0^, respectively. In contrast, these 2 peaks were almost undetectable in the LSV curves of CuO_x_@C, further demonstrating its satisfactory stability (Fig. 3L).

### The protective mechanism of carbon layer encapsulation

**Fig. 4.**
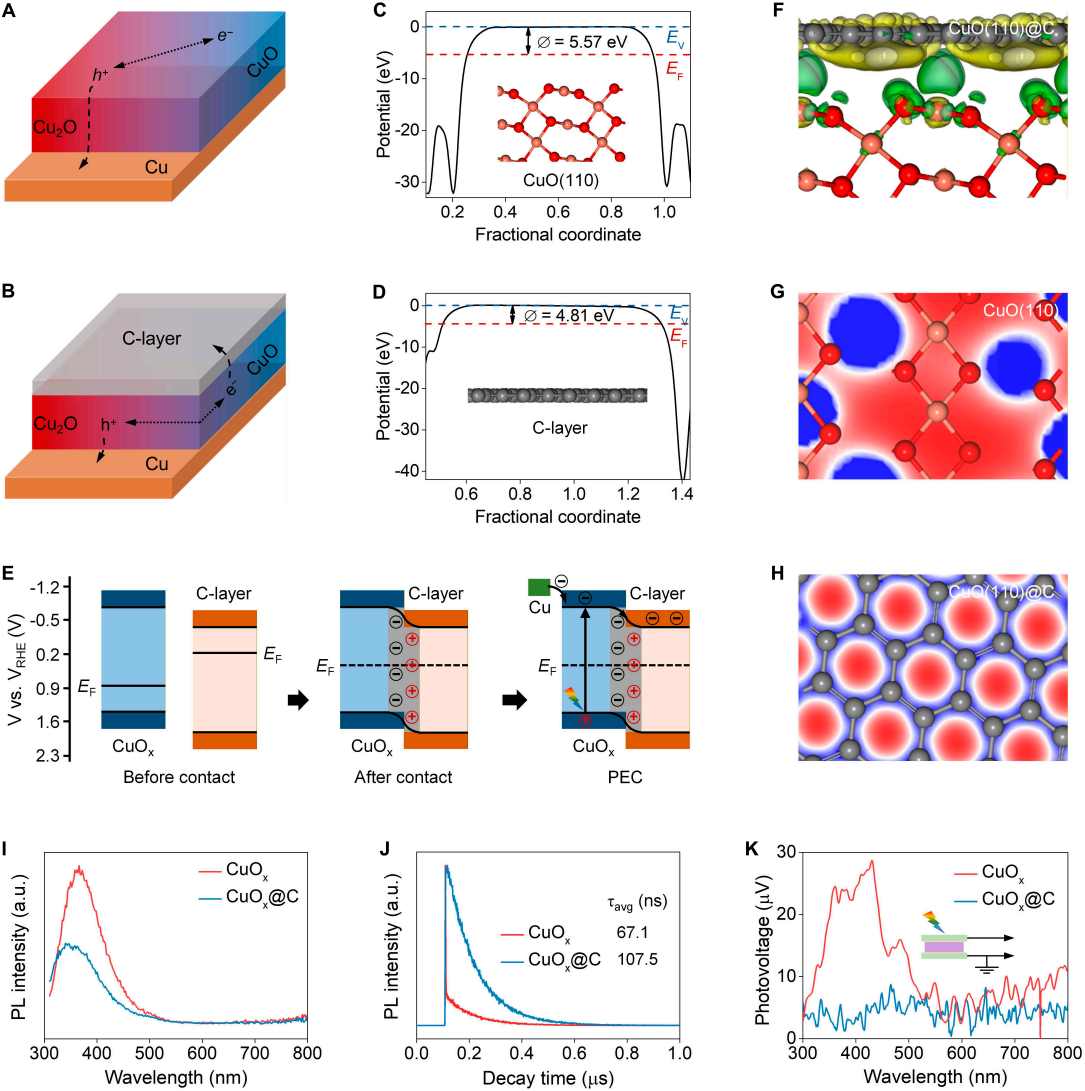
The protective mechanism of carbon layer encapsulation. Schematic diagrams of migration and separation path of photogenerated carriers in (A) CuO_x_ and (B) CuO_x_@C. Electrostatic potentials of (C) CuO(110) and (D) graphite carbon(100) (the yellow, red, and brown spheres stand for Cu, O, and C atoms; *E*_V_ denotes vacuum level). (E) Schematic illustration of CuO_x_@C heterojunction: IEF-induced interfacial charge transfer, separation, and the formation of Type II heterojunction. (F) Side-view charge density difference map of CuO(110)/graphite carbon(100) (the yellow and green regions represent net electron accumulation and depletion). Electron density distribution of (G) CuO_x_ and (H) CuO_x_@C (the blue and red regions represent net electron accumulation and depletion). The (I) steady-state PL spectra, (J) time-resolved transient PL spectra, and (K) SPV spectra of CuO_x_ and CuO_x_@C. Experimental conditions: at −0.1 V vs. RHE under AM 1.5G simulated sunlight (100 mW/cm^2^) using 0.1 M KHCO_3_ as electrolyte (CO_2_-saturated).

The carbon layer acted as an electron reservoir and effectively extracted photoelectrons from CuO_x_, resulting in spatial isolation and thus inhibiting photocorrosion (Fig. 4A and B). To accurately unveil the interfacial charge migration mechanism, the energy band structures of CuO_x_, carbon layer, and CuO_x_@C were systematically investigated (Fig. S24). Diffuse reflection spectroscopy (DRS) spectra revealed distinct optical absorption edges at ~605 nm for CuO_x_ and ~476 nm for carbon layer [30,31]. Tauc plots derived from the Kubelka–Munk transformation yielded an optical band gap (*E*_g_) of ~2.06 eV for CuO_x_ and a molecular energy gap (Δ*E*) of ~2.60 eV for the carbon layer. Ultraviolet photoelectron spectroscopy (UPS) spectra positioned the valence band (VB) of CuO_x_ at ~−5.71 eV and the highest occupied molecular orbital (HOMO) of the carbon layer at ~−6.64 eV. The conduction band (CB) of CuO_x_ was derived as ~−3.65 eV (*E*_g_ + VB), and the lowest unoccupied molecular orbital (LUMO) of the carbon layer was found at ~−4.04 eV (Δ*E* + HOMO). These energy levels were calibrated relative to the reversible hydrogen electrode (RHE) scale using the standard conversion factor (−4.44 eV for 0 V) [32]. The resulting potentials positioned the CB and VB of CuO_x_ at ~−0.79 and ~1.27 V, respectively, while the LUMO and HOMO of carbon layer were located at ~−0.40 and ~2.20 V, respectively. CuO_x_@C retained the precise band energy alignment of pristine CuO_x_, indicating that a heterojunction was formed between CuO_x_ and the carbon layer.

Density functional theory (DFT) calculation was employed to simulate carrier dynamics [33,34]. Both CuO_x_ and the carbon layer had a relatively small work function (*Φ*), which facilitated smooth electron transfer from the bulk phase to the surface (Fig. 4C and D). The Fermi levels (*E*_F_) of CuO_x_ and the carbon layer were −5.57 and −4.81 eV, respectively, indicating that the *E*_F_ of CuO_x_ was close to VB, while the *E*_F_ of the carbon layer was close to LUMO (Fig. 4E). When they came into contact with each other, electrons transferred from the carbon layer to CuO_x_ until an equilibrium state was reached, namely, the same *E*_F_ at interface. This created an internal electric field (IEF) that pointed from the carbon layer to CuO_x_. Charge density difference and partial density of states (PDOS) further confirmed that there was obvious charge redistribution at the interface, with electrons accumulating around the CuO_x_ surface (Fig. 4F and Figs. S25 and S26). Meanwhile, the IEF bent the interfacial energy bands of CuO_x_ and the carbon layer, resulting in the formation of a Type II heterojunction between CuO_x_ and the carbon layer. During photoelectrocatalytic operation, photoelectrons continuously transferred from the CB of CuO_x_ to the LUMO of the carbon layer. Electron density distribution showed that the electrons mainly accumulated around oxygen atoms in CuO_x_ (Fig. 4G). By contrast, in CuO_x_@C, electrons were extracted into carbon layer and evenly distributed around carbon atoms (Fig. 4H).

In situ Kelvin probe force microscopy (KPFM) was employed to map the spatial distribution of photocarriers in CuO_x_@C (Fig. S27) [35–37]. The surface contact potential difference (CPD) of the carbon layer-coated edge zone and central CuO_x_ area was ~380 and ~420 mV, respectively. The CPD difference (∆CPD) was up to ~40 mV, indicating that photoelectrons rapidly and directionally transferred from CuO_x_ to the carbon layer. Such fast charge migration and spatial isolation effectively suppressed electron–hole recombination and extended the photocarriers’ lifetime, further evidenced by photoluminescence (PL) analyses. There was an obvious emission peak at ~364 nm in the steady-state PL spectrum of CuO_x_, which was induced by the intrinsic radiative recombination in CuO_x_ (Fig. 4I). In contrast, the peak intensity dramatically decreased in CuO_x_@C, implying the substantial inhibition of electron–hole recombination. Moreover, the recorded transient PL decay curves indicated that the average lifetime of carriers in CuO_x_@C (~107.5 ns) was markedly longer than that in CuO_x_ (~67.1 ns) (Fig. 4J). Surface photovoltage (SPV) spectra showed that the SPV of CuO_x_@C (~8.6 μV) was much smaller than that of CuO_x_ (~27.4 μV), further suggesting that electrons transferred and accumulated in the carbon layer in CuO_x_@C (Fig. 4K). In short, the carbon layer effectively extracted photoelectrons from CuO_x_, resulting in efficient inhibition of electron-induced reduction photocorrosion and suppression of electron–hole recombination, thereby enhancing the activity and durability of CuO_x_@C (Fig. S28).

### Rational design in selective catalytic conversion

**Fig. 5.**
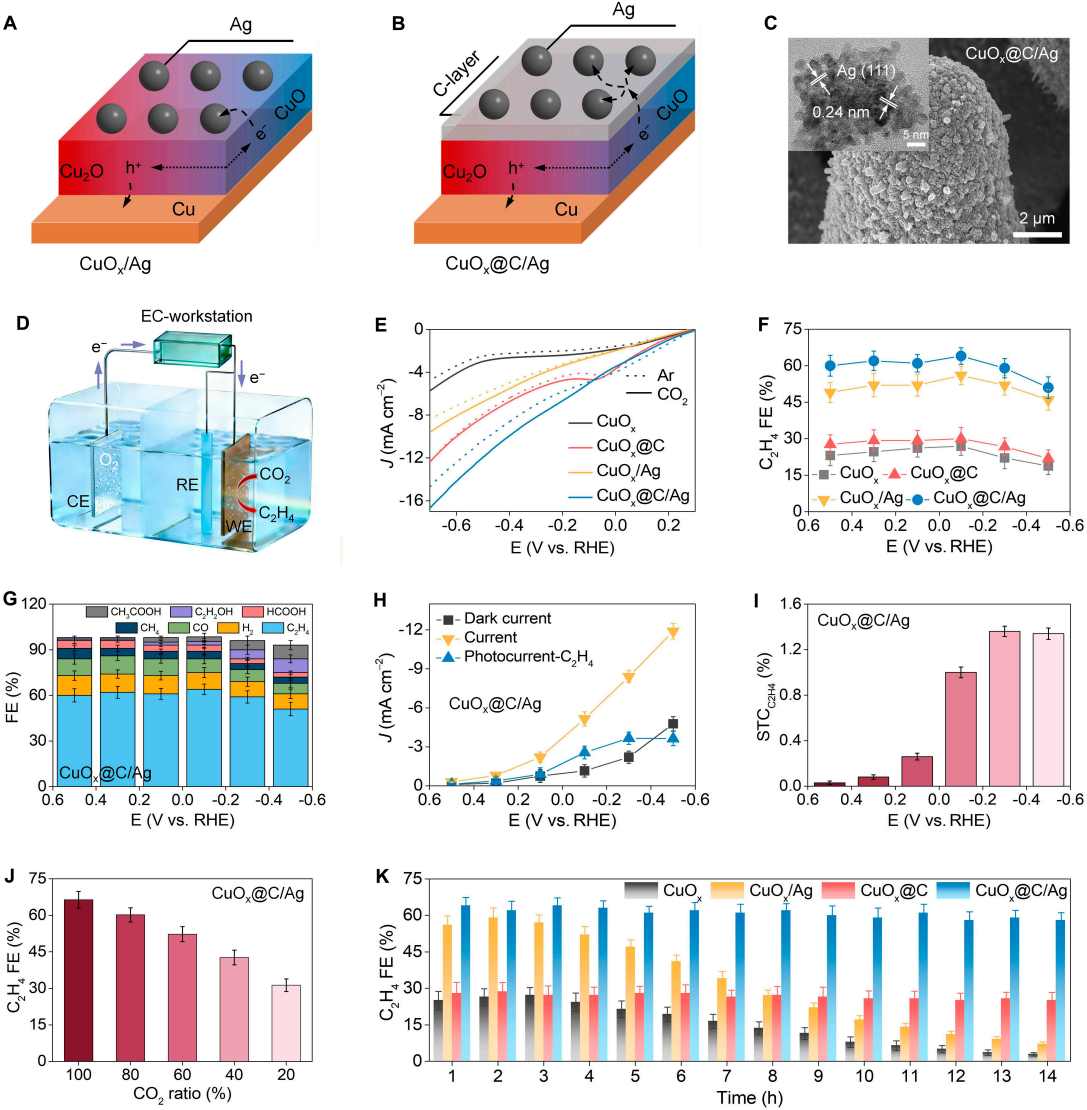
Photoelectrocatalytic activity, selectivity, and stability for CO_2_-to-C_2_H_4_ conversion. Schematic diagrams of migration and separation path of photogenerated carriers in (A) CuO_x_/Ag and (B) CuO_x_@C/Ag. (C) The SEM and TEM images of CuO_x_@C/Ag. (D) The schematic diagram of photoelectrocatalytic CO_2_ reduction. (E) The linear sweep voltammetry (LSV) curves of CuO_x_, CuO_x_@C, CuO_x_/Ag, and CuO_x_@C/Ag. (F) The C_2_H_4_ FE under different potentials on CuO_x_, CuO_x_@C, CuO_x_/Ag, and CuO_x_@C/Ag. (G) The FE of reduction products under different potentials on CuO_x_@C/Ag. (H) The photocurrent density dedicated to C_2_H_4_ production under different potentials on CuO_x_@C/Ag. (I) The STC conversion efficiency for C_2_H_4_ production under different potentials on CuO_x_@C/Ag. (J) The C_2_H_4_ FE in different initial CO_2_ concentrations on CuO_x_@C/Ag. (K) The durability of CuO_x_, CuO_x_@C, CuO_x_/Ag, and CuO_x_@C/Ag for CO_2_ reduction to C_2_H_4_. Experimental conditions: at −0.1 V vs. RHE under AM 1.5G simulated sunlight (100 mW/cm^2^) using 0.1 M KHCO_3_ as electrolyte (CO_2_-saturated). Data are presented as mean ± SD (*n* = 3 independent chemical replicates) in (D).

The CuO_x_ photoelectrodes have garnered much attention in the fields of energy conversion and environmental purification, with applications including CO_2_ reduction, nitrate reduction, and dehalogenation, among others [38–40]. To enhance the activity and selectivity of the target reactions, it was typically necessary to modify the CuO_x_ surface with specific cocatalysts, such as Ag, Ru, and Pd (Fig. 5A and Fig. S29) [28,29,41]. However, their wide application has been consistently hindered by the poor stability of CuO_x_, which was caused by severe photocorrosion. In this work, we innovatively designed and synthesized the CuO_x_@C photoelectrodes, which exhibited outstanding activity and unprecedented stability. As a result, CuO_x_@C can replace conventional CuO_x_ and be further modified with specific cocatalysts, including Ag, Ru, and Pd, to achieve large-scale applications (Fig. S30).

We have successfully prepared ternary CuO_x_@C/Ag hybrid by incorporating Ag onto the CuO_x_@C through electrodeposition and used it for CO_2_ reduction to C_2_H_4_ (Fig. 5B). SEM and EDX-mapping images revealed a uniform and dense distribution of Ag nanoparticles (~80 nm in diameter) firmly anchored on the CuO_x_@C surface in CuOx@C/Ag (Fig. 5C and Fig. S31). TEM image resolved distinct Ag (111) lattice fringes with a spacing of ~0.24 nm, confirming the excellent crystallinity of Ag nanoparticles, which was further corroborated by XRD patterns (Fig. S32) [41]. XPS spectra demonstrated that Ag nanoparticles remained predominantly in the metallic state both before and after prolonged photoelectrocatalytic CO_2_ reduction operation, establishing metallic Ag^0^ as the durable active phase in reaction, which was consistent with some previous reports (Fig. S33) [41,42]. DRS spectra showed that CuO_x_@C/Ag exhibited markedly broadened and enhanced light absorption compared to CuO_x_@C, which was mainly caused by the strong localized surface plasmon resonance of Ag nanoparticles (Fig. S34) [43]. KPFM measurements revealed a substantially enhanced ∆CPD (~60 mV) between edge zone and central area in CuO_x_@C/Ag, indicating that the Ag modification further facilitated the charge migration and separation (Fig. S35). This was further validated by the PL spectra of CuO_x_@C/Ag (Figs. S36 and S37). A substantially quenched steady-state PL emission at ~364 nm was observed, which was complemented by a markedly extended carrier lifetime of 126.4 ns in the transient PL decay. SPV spectra showed that the SPV value of CuO_x_@C/Ag was as low as ~7.7 μV, indicating that the Ag nanoparticles served as electron sinks that effectively extracted electrons from the carbon layer (Fig. S38). The aggregation of electrons on the Ag surface was highly beneficial for subsequent CO_2_ adsorption and activation. Electrochemical active surface area (ECSA) analysis indicates that carbon layer coating and Ag modification, the latter in particular, markedly increase the ECSA, thereby offering more active sites available for the subsequent CO_2_-to-ethylene reduction reaction (Fig. S39).

The photoelectrocatalytic activity, selectivity, and stability of CuO_x_@C/Ag for CO_2_ reduction to C_2_H_4_ was systematically evaluated (Fig. 5D). The CO_2_ temperature-programmed desorption tests demonstrated that Ag decoration markedly enhanced CO_2_ uptake, as evidenced by a distinct peak around 300 °C (Fig. S40). Such moderate-intensity adsorption not only facilitated efficient CO_2_ activation but also promoted the timely desorption of products, thus preventing active-site blocking. LSV measurements showed that the modification with the carbon layer, Ag, and carbon layer/Ag increased the photocurrent density of CuO_x_ by ~2.1 times (CuO_x_@C), ~1.7 times (CuO_x_/Ag), and ~2.8 times (CuO_x_@C/Ag), respectively, indicating greater electron accumulation on the CuO_x_@C/Ag surface, which was conducive to enhancing the CO_2_ reduction activity (Fig. 5E). Moreover, CuO_x_/Ag and CuO_x_@C/Ag exhibited a stronger response to CO_2_ compared to CuO_x_ and CuO_x_@C, demonstrating that Ag was a more effective active site for CO_2_ activation. The C_2_H_4_ FE of CuO_x_@C/Ag reached as high as ~66.4% at −0.1 V vs. RHE, obviously higher than that of CuO_x_ (~26.3%), CuO_x_@C (~29.1%), and CuO_x_/Ag (~54.7%), confirming the optimal selectivity of CuO_x_@C/Ag for CO_2_ reduction to C_2_H_4_ (Fig. 5F). The CuO_x_@C/Ag maintained high C_2_H_4_ FE (>60%) across a wide potential window from 0.5 to −0.1 V vs. RHE, demonstrating its operational flexibility. At potentials below −0.1 V vs. RHE, the growing contribution of electrocatalysis promoted competing reduction channels, thereby leading to a moderate decline in C_2_H_4_ FE.

In addition to C_2_H_4_, some other products, including H_2_, CO, CH_4_, HCOOH, C_2_H_5_OH, and CH_3_OOH, were also detected on CuO_x_@C/Ag (Fig. 5G). Especially at low potentials, as the proportion of electrocatalysis increased, the FE of C_2+_ products markedly improved, which was consistent with the existing reports [44,45]. The effective photocurrent density of CuO_x_@C/Ag dedicated to C_2_H_4_ production was derived by correcting the total current for dark contributions and scaling by the C_2_H_4_ FE (Fig. 5H). This value increased gradually as the potential decreases and then stabilized at potentials more negative than −0.3 V vs. RHE, reaching a maximum of ~−3.66 mA/cm^2^, suggesting full extraction of available photogenerated carriers under these conditions. Correspondingly, the solar-to-chemical (STC) conversion efficiency for C_2_H_4_ production of CuO_x_@C/Ag rose with decreasing potential before stabilizing, attaining a remarkable peak value of ~1.36% at −0.3 V vs. RHE (Fig. 5I). It was worth noting that even under a diluted CO_2_ atmosphere (~20%), CuO_x_@C/Ag retained a C_2_H_4_ FE exceeding 30%, highlighting its exceptional potential for practical deployment in real-world scenarios like industrial exhaust upgrading (Fig. 5J). Furthermore, the photoelectrocatalytic performance of CuO_x_@C/Ag toward CO_2_ reduction to ethylene was optimized by tuning the ratio of CuO to Cu_2_O (Fig. S41). The results reveal that a CuO-to-Cu_2_O ratio approaching 1:1 delivers the highest activity. This optimal performance is attributed to the formation of a more complete Type II heterostructure, which maximizes the density of Cu^2+^–O–Cu^+^ bond bridges, thereby facilitating the migration and separation of photogenerated charge carriers. The C_2_H_4_ FE of CuO_x_ and CuO_x_/Ag sharply decreased from ~26.3% to ~2.9% and from ~54.7% to ~7.6%, respectively, after 14 h of continuous use (Fig. 5K). In contrast, CuO_x_@C and CuO_x_@C/Ag maintained >95% of their initial performance after prolonged operation. The accompanying time–current curves showed that CuO_x_@C/Ag exhibited a remarkably stable current density with minimal decay, further underscoring its robust structural durability and resistance to deactivation (Fig. S42). Strikingly, the activity, selectivity, and durability of CuO_x_@C/Ag for CO_2_ reduction to C_2_H_4_ were outstanding and markedly superior to most existing studies (Fig. S43 and Table S1), demonstrating the effectiveness of the synergistic carbon encapsulation and Ag decoration strategy.

**Fig. 6.**
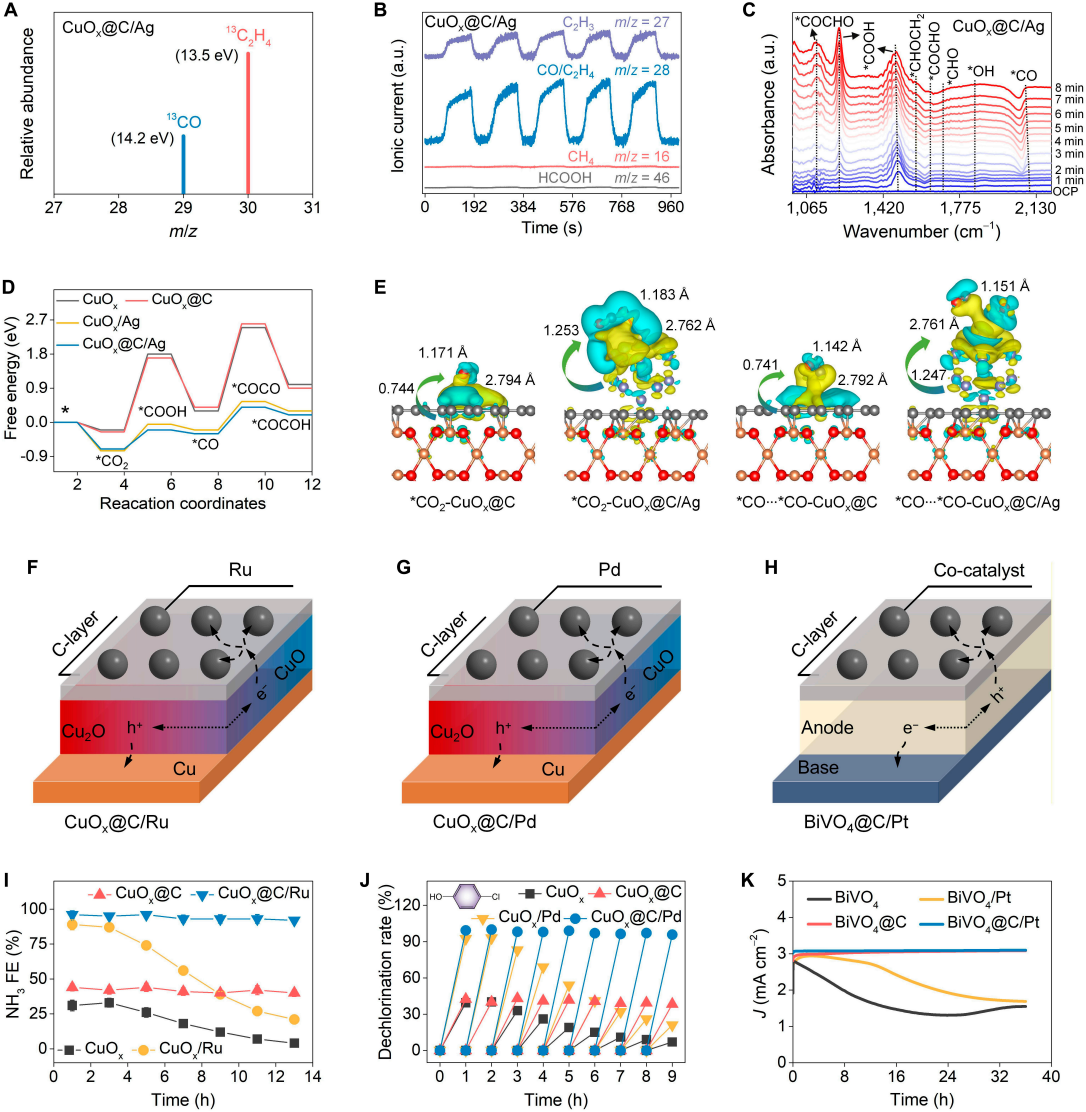
Photoelectrocatalytic mechanism for CO_2_-to-C_2_H_4_ conversion and expand applications. The (A) ^13^CO_2_ isotopic labeling experiment, (B) differential electrochemical mass spectrometry (DEMS) analysis, and (C) in situ attenuated total reflection Fourier-transform infrared (ATR-FTIR) test for CO_2_ reduction on CuO_x_@C/Ag (experimental conditions: at −0.1 V vs. RHE under AM 1.5G simulated sunlight [100 mW/cm^2^] using 0.1 M KHCO_3_ as electrolyte [CO_2_-saturated]). (D) The density functional theory (DFT) calculations of reaction pathways and free energy changes for CO_2_ reduction on different photoelectrodes. (E) Side-view charge density difference maps between different photoelectrode bases and critical intermediates (the yellow and green regions represent net electron depletion and accumulation). Schematic diagrams of migration and separation path of photogenerated carriers in (F) CuO_x_@C/Ru, (G) CuO_x_@C/Pd, and (H) BiVO_4_@C/Pt. (I) The FE of CuO_x_, CuO_x_@C, CuO_x_/Ru, and CuO_x_@C/Ru for nitrate reduction to NH_3_ (experimental conditions: at −0.1 V vs. RHE under AM 1.5G simulated sunlight [100 mW/cm^2^] using 0.1 M Na_2_SO_4_ + 10 mM NaNO_3_ as electrolyte [Ar-saturated]). (J) The rate of CuO_x_, CuO_x_@C, CuO_x_/Pd, and CuO_x_@C/Pd for dechlorination of p-chlorophenol (at −0.1 V vs. RHE under AM 1.5G simulated sunlight [100 mW/cm^2^] using 0.1 M Na_2_SO_4_ + 10 mM p-chlorophenol as electrolyte [Ar-saturated]). (K) The rate of BiVO_4_, BiVO_4_@C, BiVO_4_/Pt, and BiVO_4_@C/Pt for water oxidation (at 0.95 V vs. RHE under AM 1.5G simulated sunlight [100 mW/cm^2^] using 0.1 M Na_2_SO_4_ as electrolyte [Ar-saturated]). Data are presented as mean ± SD (*n* = 3 independent chemical replicates) in (D).

A comprehensive investigation of the reaction mechanism for CO_2_ reduction to C_2_H_4_ on CuO_x_@C/Ag was conducted. The ^13^CO_2_ isotopic labeling experiment verified that the C_2_H_4_ production originated from CO_2_ reduction rather than from other potential carbon sources (Fig. 6A). Meanwhile, the detection of ^13^CO species definitively identified CO as a pivotal intermediate in the C_2_H_4_ formation pathway [46]. The differential electrochemical mass spectrometry (DEMS) results further revealed the simultaneous generation of substantial CO (*m*/*z* = 28) and C_2_H_4_ (*m*/*z* = 28, verified by its characteristic fragment C_2_H_3_ [*m*/*z* = 27]), confirming the conversion sequence of CO_2_ → CO → C_2_H_4_ (Fig. 6B) [47]. The absence of DEMS signals of CH_4_ (*m*/*z* = 16) and HCOOH (*m*/*z* = 46) further underscored the remarkable selectivity toward C_2_H_4_ production on CuO_x_@C/Ag. The in situ attenuated total reflection Fourier-transform infrared (ATR-FTIR) spectroscopy collected on CuO_x_@C/Ag showed that the distinct vibrational signatures of *CO (~2,053.1 cm^−1^) and *COCHO (~1,112.2 and 1,637.3 cm^−1^) were observed, while the characteristic *CHO band (~1,698.6 cm^−1^) remained absent (Fig. 6C) [48,49]. This provided compelling evidence that the crucial C–C coupling step proceeded predominantly through symmetric dimerization of 2 *CO intermediates, rather than the alternative asymmetric *CO–*CHO pathway. The reaction cascade was reconstructed through further identification of additional intermediates, including *COOH (~1,216.2 and 1,480.3 cm^−1^) and *CHOCH_2_ (~1,567.2 cm^−1^), collectively establishing the general reaction sequence of *CO_2_ → *COOH → *CO → *COCO → *COCHO → *CHOCH_2_ → *C_2_H_4_. In contrast, all these intermediate signals were markedly attenuated on the Ag-free CuO_x_@C, consistent with its inferior activity and C_2_H_4_ selectivity, and highlighting the essential role of Ag in promoting intermediate stabilization and the decisive C–C coupling step (Fig. S44).

DFT calculations provided atomic-scale validation for the proposed reaction pathway (Fig. 6D and E and Fig. S45). The introduction of Ag markedly enhanced CO_2_ adsorption, with adsorption energies strengthening from −0.25 eV (CuO_x_@C) to −0.75 eV (CuO_x_@C/Ag). The hydrogenation barriers of *CO_2_ to *COOH dramatically reduced from 1.95 eV on CuO_x_@C to 0.5 eV on CuO_x_@C/Ag. This enhancement was further corroborated by differential charge density analysis, revealing substantial electron transfer between *CO_2_ and CuO_x_@C/Ag (Bader charge: 1.253 |e|) compared to only 0.744 |e| with CuO_x_@C. The strengthened interaction facilitated C–O bond activation (from 1.171 to 1.183 Å) and enabled efficient *CO_2_ hydrogenation. The resulting *CO intermediate was effectively stabilized on CuO_x_@C/Ag, with adsorption energies of −0.3 eV, in contrast to positive values (0.4 eV) on CuO_x_@C, where *CO tended to desorb. The comparison of formation barriers for *CO (−0.1 eV) and *HCOOH (0.1 eV) provided a theoretical foundation for the experimentally observed minimal HCOOH production. On CuO_x_@C/Ag, the C–C coupling barrier via *CO dimerization (~0.7 eV) was markedly lower than the barrier for *CO hydrogenation to *CHO (~0.9 eV), establishing the thermodynamic preference for symmetric *CO dimerization over asymmetric *CO–*CHO pathways. Meanwhile, the unfavorable *CHO formation fundamentally explained the suppressed productions of CH_4_ and CH_3_OH observed experimentally. In stark contrast, CuO_x_@C exhibited prohibitively high C–C coupling barrier (~2.2 eV). Further evidence emerged from the substantial charge transfer between *CO and CuO_x_@C/Ag (~1.247 |e| versus ~0.741 |e| for CuO_x_@C) and strengthened C–O bond activation (from 1.142 to 1.151 Å). The complete reaction pathway on CuO_x_@C/Ag for CO_2_ reduction to C_2_H_4_ proceeded through an optimized sequence of *CO_2_ → *COOH → *CO → *COCO → *COCHO → *COCHOH → *CHOCHOH → *CHOCH_2_OH → *CHOCH_2_ → *CHOHCH_2_ → *CH₂OHCH_2_ → *C_2_H_4_, demonstrating how Ag incorporation simultaneously enhanced CO_2_ adsorption, stabilized critical intermediates, and promoted selective C–C coupling toward C_2_H_4_ production.

To establish the general applicability of the coupled carbon-encapsulation and metal-decoration strategy, we further engineered a series of functional photoelectrodes, including CuO_x_@C/Ru, CuO_x_@C/Pd, and BiVO_4_@C/Pt (Fig. 6F to H). These systems were respectively deployed for nitrate reduction to NH_3_, chlorophenol dechlorination, and the oxygen evolution reaction, respectively (Fig. S30). A particularly noteworthy configuration was realized in the BiVO_4_@C/Pt, where the carbon layer served as an efficient hole-extraction mediator. This led to spatial isolation of photoholes, thereby inhibiting hole-induced oxidation photocorrosion and suppressing electron–hole recombination. The FE for nitrate reduction to NH_3_ on CuO_x_ and CuO_x_/Ru sharply reduced from ~31.4% to ~4.1% and from ~89.1% to ~21.3%, respectively, after 14 h of continuous use (Fig. 6I). By contrast, CuO_x_@C and CuO_x_@C/Ru maintained ~95% of their initial FE for nitrate reduction to NH_3_ after prolonged use. As shown in Fig. 6J, the dechlorination rate of p-chlorophenol on CuO_x_ and CuO_x_/Pd sharply reduced from ~39.2% to ~7.4% and from ~92.3% to ~20.7%, respectively, after 9 h of continuous use. By contrast, CuO_x_@C and CuO_x_@C/Pd maintained ~97% of their initial dechlorination rate after prolonged use. The photocurrent densities of BiVO_4_ and BiVO_4_/Pt decreased over time, remaining at only ~57% of their initial values after 40 h of chronoamperometry (Fig. 6K). By contrast, BiVO_4_@C and BiVO_4_@C/Pt exhibited stable photocurrent during continuous operation, maintaining ~98% of their initial photocurrent density at the end of the test. It was worth mentioning that the activity and durability of CuO_x_@C/Ru, CuO_x_@C/Pd, and BiVO_4_@C/Pt were outstanding and markedly superior to most existing studies (Fig. S43 and Table S1), demonstrating the synergistic carbon encapsulation and cocatalyst decoration strategy.

## Conclusion

In summary, we unveiled the photoelectrochemical transformation mechanism of CuO_x_ during the reaction, namely, the stepwise photoelectron reduction: Cu_2_O/CuO → Cu_2_O → Cu. To overcome these changes, we innovatively proposed an ultrathin carbon layer encapsulation strategy formed via electrodeposition-coupled self-assembly of carbon nanodots. The synthesized CuO_x_@C exhibits stable phase and photocurrent throughout prolonged operation exceeding 24 h. We then further modified CuO_x_@C with different cocatalysts, including Ag, Ru, and Pd, to improve the activity and selectivity of the target reactions. The results showed that the Faraday efficiencies for CO_2_ reduction to C_2_H_4_ on CuO_x_@C/Ag, nitrate reduction to NH_3_ on CuO_x_@C/Ru, and dechlorination of p-chlorophenol on CuO_x_@C/Pd were as high as ~66.4%, ~97.5%, and ~99.2%, respectively, with each maintaining >95% of its initial performance after prolonged use. Additionally, this protection approach was successfully extended beyond CuO_x_ to other photoelectrodes, such as BiVO_4_. This work provides insights into investigating the chemical alterations in metal oxides under reaction conditions and opens new avenues for the rational design of efficient and durable photoelectrocatalytic materials to realize sustainable energy conversion and environmental purification.

## Materials and Methods

Information about the materials and methods used in this work is provided in the Supplementary Materials.

## Data Availability

The data that support the findings of this study are available from the corresponding author upon reasonable request.
