# Supplementary material for: Synergistic Carbon Encapsulation and Silver Decoration Enable Durable and Selective CO2-to-Ethylene Conversion on Copper Oxide Photoelectrode
Source: Research (Wash D C). 2026 Mar 16;9:1206. doi: 10.34133/research.1206 (PMC12989650; doi:10.34133/research.1206)
Supplement: Supplementary 1 — Supplementary Methods Figs. S1 to S45 Table S1 [file research.1206.f1.doc]

*Supplementary materials for:*

**Synergistic carbon encapsulation and silver decoration enable durable and selective CO_2_-to-ethylene conversion on copper oxide photoelectrodes**

*Songying Qu^a^**, Ruiquan Yu^b^, Min Gao^b^, Jun Zhang^c^,**

*a, Macao Institute of Materials Science and Engineering, Faculty of Innovation Engineering, Macau University of Science and Technology, Taipa 999078, Macao SAR*

*b, Institute of Environment and Ecology, Tsinghua Shenzhen International Graduate School, Tsinghua University, Shenzhen 518055, China*

*c,* *Advanced Interdisciplinary Institute of Environment and Ecology, Guangdong Provincial Key Laboratory of Wastewater Information Analysis and Early Warning, School of Technology for Sustainability, Beijing Normal University, Zhuhai 519087, China*

Corresponding Author.

(J. Zhang) E-mail: [zhangboyu2016@163.com](mailto:zhangboyu2016@163.com)

Supporting information includes:

60 Pages 45 Figures 1 Tables 5532 Words

**Materials and methods**

**1. Chemicals and materials**

All chemical reagents were purchased from Aladdin reagent Co., LTD (Shanghai, China) and used directly without further purification. Ethyl alcohol (AR, 99.5%), acetone (AR, 99.9%), dimethyl sulfoxide (DMSO, AR, 99.9%), sodium sulfate (Na_2_SO_4_, ACS, ≥99%), potassium bicarbonate (KHCO_3_, AR, 99.5%), sodium nitrate (NaNO_3_, AR, 99%), sodium hydroxide (NaOH, ACS, 97%), sodium sulfate (H_2_SO_4_, 70%), hydrochloric acid (HCl, 36~38%), p-chlorophenol (4-CP, AR, 99%), phenol (AR, 99%), triethanolamine (TEOA, AR, 98%), acetonitrile (ACN, ≥99.0%), methanol (AR, 99.5%), formic acid (AR, ≥98%), p-benzoquinone (BQ, AR, ≥98%), ammonium chloride (NH_4_Cl, AR, ≥97%), RuCl_3_ (AR, ≥96%), PdCl_2_ (AR, ≥94%), H_2_PtCl_6_ (AR, ≥96%), potash iodide (KI, AR, ≥98%), bismuth nitrate (Bi(NO_3_)_3_, AR, ≥98%). Gaseous reagents, including argon (Ar) and carbon dioxide (CO_2_) were all high purity (≥99.99%) and purchased from Kaifeng Industrial Development Co., LTD (Shenzhen, China). Ti plate (≥99.99%, rectangular shape, 1 cm × 2 cm, 1 mm of thickness), Pt electrode, Ag/AgCl electrode, Fc^+^/Fc electrode, proton exchange membrane (PEM), single-chamber PEC cell, H-type PEC cell, and in-situ PEC Raman and FTIR cells were all purchased from Aida Hengsheng Technology Development Co., LTD (Tianjin, China).

**2. Photoelectrodes synthesis**

All chemical reagents were purchased from Aladdin Reagent Co., Ltd. (Shanghai, China) and used directly without further purification. The carbon nanodots were synthesized via an electrochemical etching method. Two high-purity graphite rods were used as the anode and cathode and were inserted into ultrapure water with a separation distance of 7 cm. A bias potential of 30 V was supplied by a regulated DC power source. After continuous operation for 120 h, the suspension was centrifuged at 28 000 rpm for 1 h. The collected supernatant was then dried at 60 °C for 24 h to obtain carbon nanodot powders. CuO_x_ (Cu/Cu_2_O/CuO) was prepared by a one-step calcination method. A high-purity copper foam (1 cm × 1 cm × 0.1 cm) was cleaned using 1.0 M HCl and ultrapure water, and then placed in a muffle furnace for a two-stage calcination process. Initially, a layer of Cu_2_O formed on the Cu surface after calcination at 300 °C for 2 h. Subsequently, the Cu/Cu_2_O was transformed to Cu/Cu_2_O/CuO after treatment at 500 °C for 1 h (**Figure S1**). An ultra-thin carbon layer was deposited on the CuO_x_ surface via an innovative electrodeposition method coupled with the self-assembly of carbon nanodots to prepare CuO_x_@C. A standard two-electrode system was used, with CuO_x_ and Pt serving as the work electrode and counter electrode, respectively. The electrolyte consisted of 0.1 g/L carbon nanodots and 5 mM Na_2_SO_4_. A constant bias of 0.4 V was applied for 30 min to complete the encapsulation process. The surface modification of CuO_x_@C using Ag, Ru, and Pd was performed by electrodeposition. CuO_x_@C and Pt were used as working electrode and counter electrode, respectively. The electrolyte consisted of 10 mM AgNO_3_ or RuCl_3_ or PdCl_2_ and 10 mM NH_4_Cl. A constant bias of −0.5 V was applied for 10 min to complete the loading process. BiVO_4_ was synthesized by a well-established ion exchange method as reported previously (Supplementary Information). The BiVO_4_@C and BiVO_4_@C/Pt were prepared using similar above electrodeposition methods. The results of inductively coupled plasma-mass spectrometry (ICP-MS) analysis indicated that the loading amounts of Ag, Ru, Pd, and Pt nanoparticles were ~5, ~6, ~3, and ~3 mg/g_catalyst_, respectively.

BiVO_4_ sample was fabricated following the typical procedure reported in the literature^1-3^. In brief, BiOI nanoflakes were firstly electrodeposited on the pre-cleaned FTO substrate from a 0.04 M Bi(NO_3_)_3_ solution containing 0.4 M KI and 0.23 M p-benzoquinone for 5 min. Subsequently, 150 µL of DMSO solution containing 0.2 M vanadyl acetylacetonate was impregnated on the prepared BiOI sample at 60 °C and then calcined in Air at 450 °C for 2 h with a ramping rate of 2 °C/min. The obtained samples were soaked in 1 M NaOH solution for 30 min with gentle stirring to remove the impurities. Lastly, the samples were sequentially rinsed with water and ethanol.

**3. Characterization**

Scanning electron microscopy (SEM) (GeminiSEM 500, Zeiss, Germany) and transmission electron microscopy (TEM) (JEM-2100F, JEOL, Japan) were used to observe the morphology of the materials. X-ray diffractometer (XRD) (D8 Discover, Bruker, Germany), and Raman spectrograph (LabRAM HR Evolution, HORIBA Scientific, Japan) were employed to examine the phase structure of materials. The surface chemical states of the materials were investigated using X-ray photoelectron spectroscopy (XPS) (ESCALAB 250Xi, Thermo Scientific, USA) and attenuated total reflection Fourier-transform infrared spectroscopy (ATR-FTIR) (Nicolet iS10, Thermo Scientific, USA). Diffuse reflectance spectrum (DRS) (U-3900, Hitachi, Japan) and incident photon-to-electron conversion efficiency (IPCE) were performed to assess the photoelectric conversion characteristic of the materials. Steady-state photoluminescence (PL) emission spectra and time-resolved transient PL decay spectra (FLS1000, Edinburgh, UK) were used to analyze the migration and separation of photogenerated carriers. Surface photovoltage spectroscopy (SPV) (PL-SPV/IPCE1000, Perfectlight, China) was used to analyze the voltage changes on the surface of materials. Kelvin probe force microscopy (KPFM) (Dimension FastScan, Bruker, USA) was carried out to record the surface potential of the materials. The CO_2_ temperature-programmed desorption (CO_2_-TPD) tests were carried out on a fully automatic chemical adsorption instrument (ChemiSorb 2720, Micromeritics, USA). An inductively coupled plasma-mass spectrometry (ICP-MS, Agilent 7900, USA) was operated to determine the atomic ratio and loading amount of metal element during the materials synthesis and quantify the contents of released metal ions during the reaction.

For SEM, XRD, XPS, UPS, Raman, ATR-FTIR, and CO_2_-TPD measurements: Intact electrode sheets (3 mm × 3 mm) were analyzed without any pretreatment. For SEM, the electrode was directly adhered to conductive adhesive tape. For XRD, XPS, UPS, and CO_2_-TPD, the electrode was secured onto the respective sample holders. For Raman and ATR-FTIR analyses performed under operational conditions, the electrode was firmly attached to the working electrode stage of an in-situ cell using conductive adhesive. These measurements were conducted at an applied potential of −0.1 V vs. RHE in a CO_2_-saturated 0.1 M KHCO_3_ aqueous electrolyte under illumination from an AM 1.5G solar simulator (100 mW/cm^2^). For TEM and KPFM measurements: To facilitate these analyses, the electrode material underwent a pretreatment procedure. The electrode sheet was immersed in deionized water and subjected to ultrasonic exfoliation to dislodge micron- and nano-scale particles into the solution. The resulting suspension was then drop-cast onto ultrathin carbon-coated copper grids (for TEM) or silicon wafers (for KPFM) for subsequent analysis. KPFM measurements were performed under AM 1.5G simulated sunlight (100 mW/cm^2^). For DRS and IPCE measurements: Intact electrode sheets (10 mm × 10 mm) were used without modification. For DRS, the electrode was mounted directly in a sample holder equipped with a BaSO_4_ background plate, where BaSO_4_ served as the 100% reflectance reference standard. IPCE measurements were conducted using a commercial photoelectrochemical test system (Zennium, Zahner) equipped with a monochromator (TLS-03, Zahner). A constant potential of −0.1 V vs. RHE was applied in a CO_2_-saturated 0.1 M KHCO_3_ aqueous electrolyte. Monochromatic light was generated by a 300 W Xe lamp coupled with a monochromator, and the light intensity at each wavelength was calibrated using a standard Si photodiode. The IPCE values were calculated using the equation: IPCE (%) = (1240 × I_s_c) / (λ × P_light_) × 100%, where I_s_c is the short-circuit photocurrent density (A/cm^2^), λ is the wavelength (nm), and P_light_ is the incident light power density (W/cm^2^). For PL and SPV measurements: A powder sample preparation was required. A small amount of material was scraped from the bulk electrode using a spatula and thoroughly ground using an agate mortar. For PL spectroscopy, the resulting powder was directly placed in a sample well and leveled. For SPV measurements, the powder was pressed into a pellet to ensure good electrical contact and uniformity, then mounted onto an ITO substrate to form a sandwich-like configuration for testing. A 300 W xenon lamp coupled with a monochromator provided monochromatic light, which was modulated at 20 Hz using a mechanical chopper for the SPV measurements.

**4. Photoelectrochemical test**

A standard three-electrode system was adopted, with the as-prepared photoelectrodes, Pt, and Ag/AgCl serving as working electrode, counter electrode, and reference electrode, respectively. The electrolyte consisted of 0.1 M Na_2_SO_4_, and the solution pH was adjusted using 1 M NaOH and 0.5 M H_2_SO_4_. The electrolyte was deaerated before the experiments by continuously bubbling Ar with a 20 mL/min flow rate. A constant Ar flow (10 mL/min) was used to maintain the inert atmosphere during chronoamperometry. A 300 W xenon lamp (CEL-HXF300-T3, China Education Au-light, China) was used to simulate solar light. The area of the photoelectrodes was about 1 cm^2^ and the tests were performed under simulated AM 1.5G sunlight at 100 mW/cm^2^, which was calibrated with a power meter (Solar Light, PMA2100). The electrochemical system was controlled by an electrochemical workstation (AUTOLAB PGSTAT302N, Metrohm, Switzerland). The electrochemical analysis included techniques such as linear sweep voltammetry (LSV, 10 mV/s), electrochemical impedance spectroscopy (EIS, 10^−2^‒10^6^ Hz), and chronoamperometry.

**5. Analytical methods**

A front-illuminated photoelectrochemical cell was used for the study of CO_2_ reduction to C_2_H_4_ experiments. A quartz window was placed in line with the photocathode to allow the light generated from a solar simulator to shine on the photocathode, which is in contact with the electrolyte. The area of the photocathode was about 1 cm^2^ and the tests were performed under simulated AM 1.5G sunlight at 100 mW/cm^2^, which was calibrated with a power meter (Solar Light, PMA2100). The electrolyte used for CO_2_ reduction to C_2_H_4_ experiments is an aqueous solution of 0.1 M KHCO_3_. The H_2_O and KHCO_3_ jointly functioned as the donors of electrons and protons during the CO_2_ reduction process. The overall process comprises two half-reactions: the photoelectrocatalytic CO_2_ reduction taking place at the photocathode, coupled with the electrocatalytic water oxidation occurring at the Pt anode. The anodic and cathodic solutions were deaerated before the experiments by continuously bubbling CO_2_ with a 20 mL/min flow rate. A constant CO_2_ flow (10 mL/min) was used to maintain the CO_2_ atmosphere during chronoamperometry. All the products were detected with an inline gas chromatograph (GC, SRI 8610C, USA). The amount of gas products detected by the GC were averaged over the testing period to obtain the Faradaic efficiency of different products. The experiments of nitrate reduction to NH_3_ and dechlorination of p-chlorophenol were conducted using a custom-made H-type photoelectrochemical cell, in which the cathodic and anodic compartments were separated by a proton exchange membrane (PEM). The anodic and cathodic solutions were deaerated before the experiments by continuously bubbling Ar with a 20 mL/min flow rate. A constant Ar flow (10 mL/min) was used to maintain the inert atmosphere during chronoamperometry. The electrolyte used for nitrate reduction to NH_3_ experiments is an aqueous solution of 0.1 M Na_2_SO_4_ + 10 mM NaNO_3_. The produced NH_3_ was quantitatively determined using the indophenol blue method. The ultraviolet-visible (UV-Vis) absorbance spectra were measured on a PERSEE TU-1950 UV-vis spectrophotometer. The electrolyte used for dechlorination of p-chlorophenol experiments is an aqueous solution of 0.1 M Na_2_SO_4_ + 10 mM p-chlorophenol. A high-performance liquid chromatography (HPLC) system (Alliance e2695) equipped with a 4.6 × 250 mm, 5 μm Venusil HILIC column was operated to detect the concentration of p-chlorophenol and phenol. A single-chamber photoelectrochemical cell was used for the experiments of water oxidation. The electrolyte is an aqueous solution of 0.1 M Na_2_SO_4_.

The FE value for CO_2_ reduction to ethylene was calculated based on the following equation^4-6^:

*FE* = *Q*_ethylene_/*Q*_total_ = *n*_ethylene_*NF*/*Q*_total_ = ((*v*/60S/min) × (*y*/22400cm^3^/mol)*NF*)/*j* × 100% (1)

where *v* is the gas flow rate measured by a flowmeter, *y* is the volume concentration of gas products, *N* represents the number of transferred electrons for each product, *F* denotes the Faraday constant (96,500 C/mol), *j* signifies current, *t* corresponds to the running time.

The effective photocurrent density (*I*_P_) dedicated to C_2_H_4_ production was derived by correcting the total current density for dark contributions and scaling by the C_2_H_4_ FE:

*I*_P_ = (*I*_T_ – *I*_D_) × *FE*_ethylene_ (2)

where *I*_P_ is the photocurrent density dedicated to C_2_H_4_ production, *I*_T_ is the total current density, *I*_D_ is the dark current density, FE_ethylene_ is the C_2_H_4_ FE.

The solar-to-chemical (STC) conversion efficiency for C_2_H_4_ production was calculated based on the following equation^7^:

*STC* = (R_C2H4_ × Δ*H*^0^_combustion (C2​H4​)_​)/(*I*_solar_ × *A*_illuminated_) × 100% (3)

Where R_C2H4_ is the C_2_H_4_ production rate, Δ*H*^0^_combustion (C2​H4​)_​ is the combustion enthalpy for C_2_H_4_, *I*_solar_ is the light intensity, *A*_illuminated_ is the illuminated area.

The FE value for nitrate reduction to NH_3_ was calculated via the following relation^8-10^:

*FE* = (8 × *F* × *C*_NH3_ × *V*)/(17 × *Q*) (4)

where *C*_NH3_ is the measured NH_3_ concentration, *V* is the volume of the electrolyte, *F* is the Faraday constant (96,500 C/mol) and *Q* is the total charge.

The dechlorination rate of p-chlorophenol was calculated using the following equation^11-13^:

*D* = *N*_t_/*N*_0_ × 100% (5)

where *D* (%) is the dechlorination rate, *N*_0_ is the theoretical maximum concentration of generated Cl^−^ before treatment, *N*_t_ represents the measured concentration of the Cl^−^ after treatment.

In-situ differential electrochemical mass spectrometry (DEMS) was performed with a Hiden HPR40 spectrometer interfaced with a photoelectrochemical cell under AM 1.5G simulated sunlight (100 mW/cm^2^)^14^. The cathodic and anodic chambers, separated by a Nafion^TM^ 212 membrane, were continuously supplied with 0.1 M KHCO_3_ electrolyte at 0.5 mL min^−1^ via a peristaltic pump. Volatile products were selectively sampled into the mass spectrometer through a PTFE membrane interface. The electrolyte was pre-saturated and continuously purged with CO_2_ throughout the measurements. Linear sweep voltammetry (LSV) from 0.3 to −0.2 V vs. RHE was applied at a scan rate of 10 mV s^−1^. All mass-to-charge signals were recorded using a secondary electron multiplier detector set at 1000 V.

**6. Theoretical calculations**

First-principles calculations within the framework of density functional theory (DFT) were performed to investigate the structural and electronic properties using the Cambridge Sequential Total Energy Package (CASTEP). The exchange-correlation functional was described using the generalized gradient approximation (GGA) with norm-conserving pseudopotentials and the Perdew-Burke-Ernzerhof (PBE) functional. Structural optimizations and transition state calculations were performed using the DMol^3^ code. An energy cutoff of 750 eV was employed, and a k-point sampling grid of 5 × 5 × 1 was used, which was found to be converged. The calculation parameters included a force tolerance of 0.01 eV/Å, an energy tolerance of 5.0 × 10^−7^ eV per atom, and a maximum atomic displacement of 5.0 × 10^−4^ Å. A vacuum region of 15 Å along the z-direction was introduced to prevent interactions between neighboring periodic images. The Grimme method for DFT-D correction was applied in all calculations. The CuO(110), graphite carbon(100), and CuO(110)@C surfaces were constructed, with the bottom three atomic layers being fixed while the top three atomic layers were allowed to relax.

**7. Theoretical rationale for Ru and Pd** **modifications**

Extensive literature demonstrates that Ru modification serves as an active center to effectively enhance nitrate adsorption, promote atomic hydrogen (H*) supply, and stabilize key intermediates such as *NO^15,16^. Consequently, it significantly facilitates the hydrogenation of nitrate to ammonia while suppressing N–N coupling side reactions, thereby improving both activity and selectivity toward NH_3_ formation^17,18^. On the other hand, Pd is widely recognized as an efficient hydrogenation metal with strong hydrogen adsorption and activation capabilities^19,20^. Its incorporation can substantially increase the localized H* availability, strengthen the adsorption and enrichment of halogenated organic compounds, and lower the energy barrier for hydrodechlorination^21,22^. This leads to highly efficient and selective removal of halogen atoms, making Pd an optimal candidate for dehalogenation catalysis. Together, Ru and Pd offer distinct and complementary functions, targeting nitrate-to-ammonia conversion and halogenated pollutant degradation, respectively, through enhanced reactant adsorption, optimized hydrogen utilization, and stabilization of critical reaction pathways.


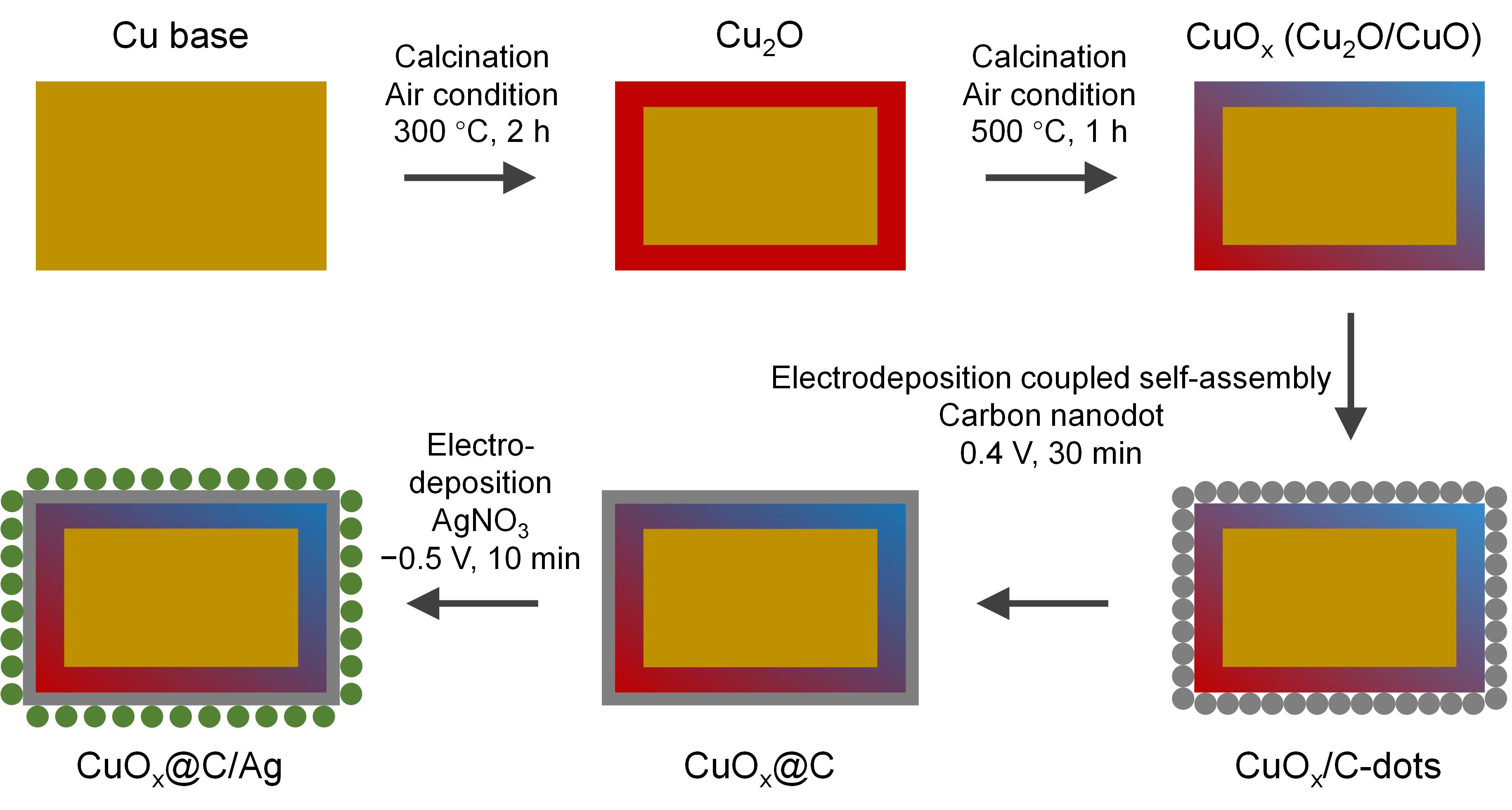


**Figure S1.** Schematic diagram of synthesis path of CuO_x_@C/Ag photoelectrode.

The synthesis of the CuO_x_@C/Ag nanocomposite proceeds through a sequential thermal and electrochemical strategy (**Figure S1**). A high-purity copper substrate is first transformed into a mixed-phase copper oxide (CuO_x_, comprising Cu_2_O/CuO) via a two-step calcination in air: initial oxidation at 300 °C for 2 h forms an ultrathin Cu_2_O layer, which is subsequently converted into a Cu_2_O/CuO heterostructure at 500 °C for 1 h. This CuO_x_ template is then conformally encapsulated within a continuous carbon layer through an innovative electrodeposition-coupled self-assembly process using carbon nanodots (C-dots). Applying a potential of 0.4 V for 30 min drives the initial attachment of C-dots, followed by a chemical interaction-driven self-assembly that yields a uniform carbon coating, resulting in the CuO_x_@C intermediate. Finally, silver nanoparticles are densely and uniformly deposited onto the CuO_x_@C surface via electrodeposition from an AgNO_3_ solution at −0.5 V for 10 min. This step produces the ternary CuO_x_@C/Ag architecture, which integrates a redox-active oxide core, a conductive carbon matrix, and dispersed Ag nanoparticles, offering synergistic functionality for advanced applications.


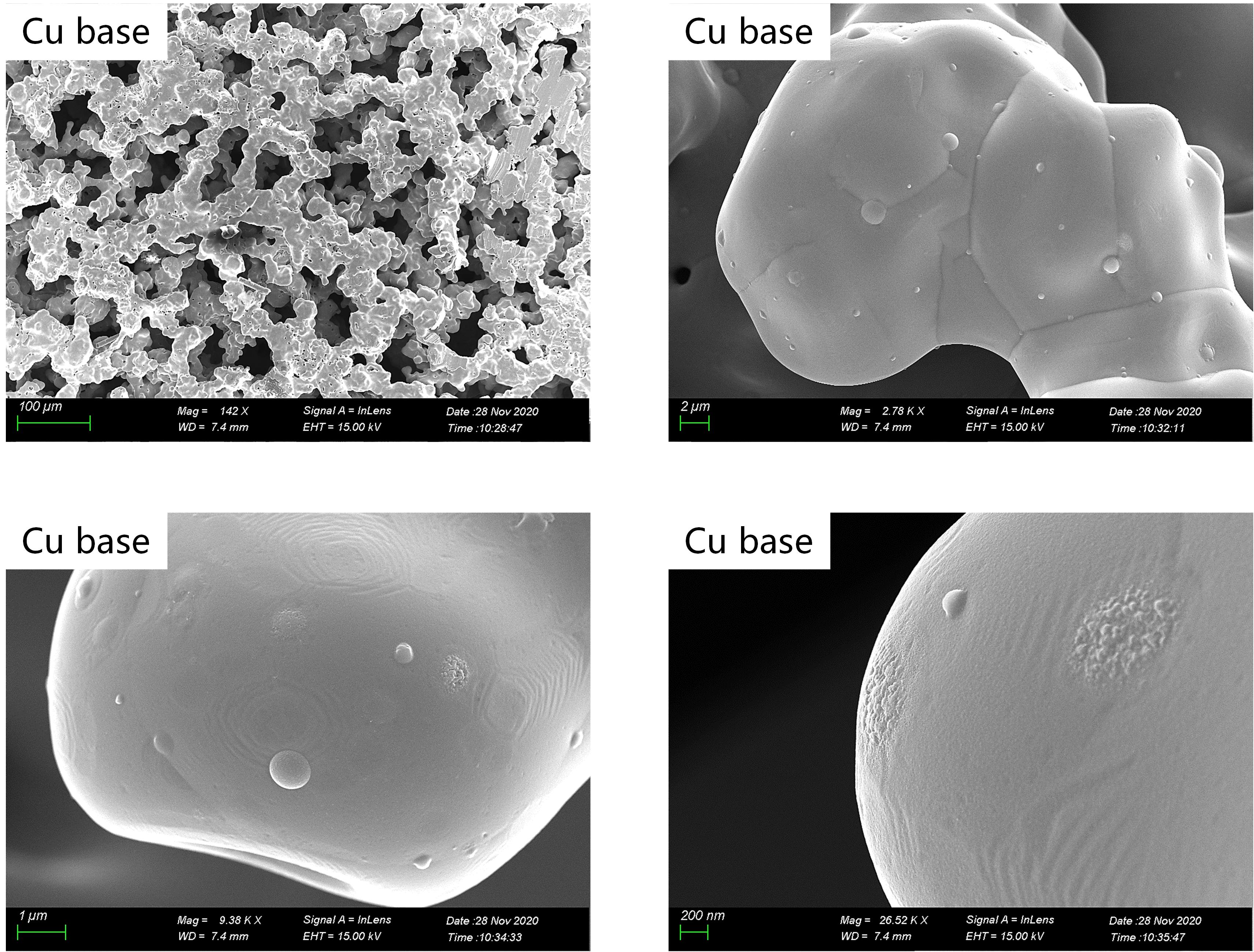


**Figure S2.** The SEM images of pristine Cu base.


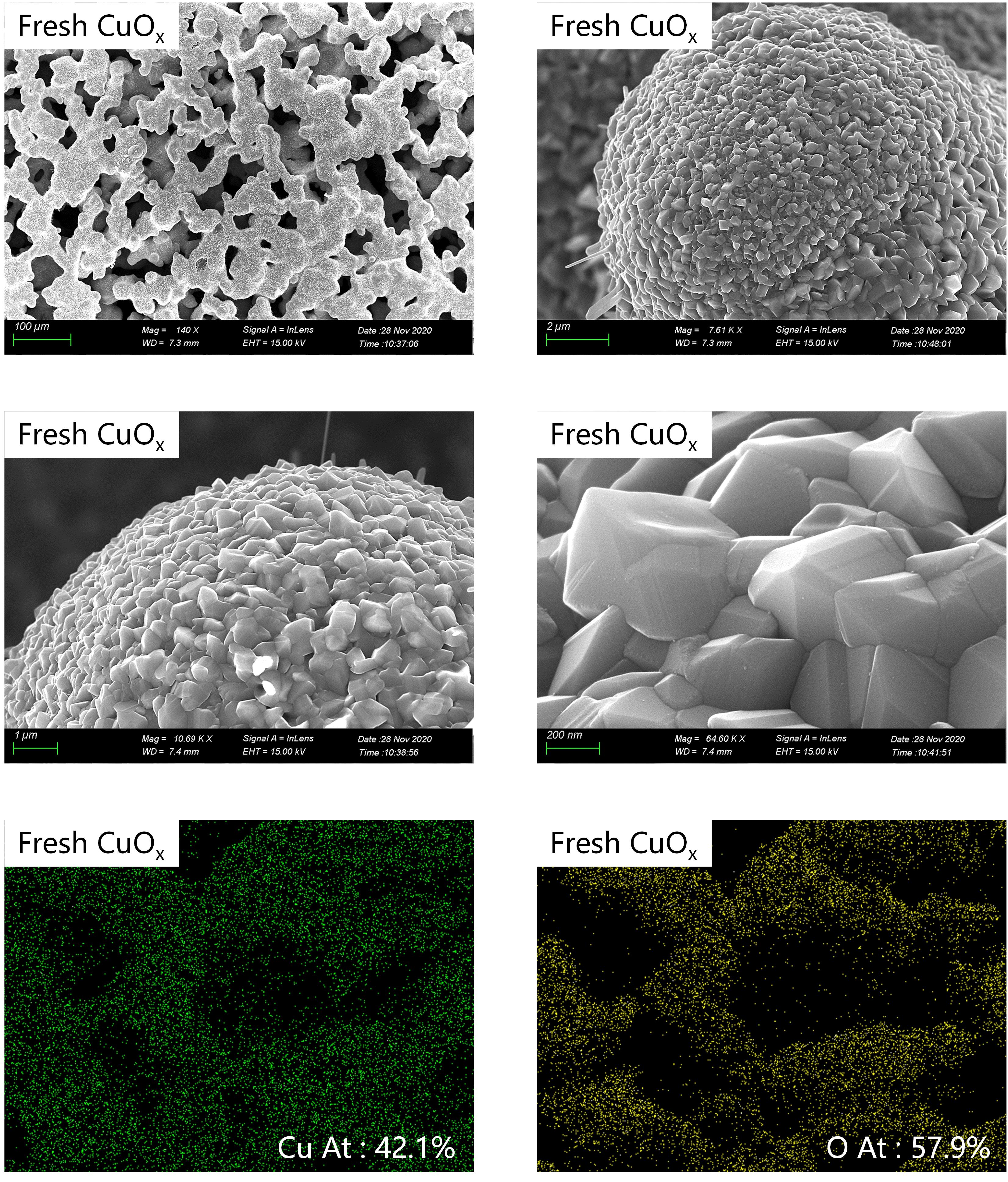


**Figure S3.** The SEM and EDX-mapping images of fresh CuO_x_.


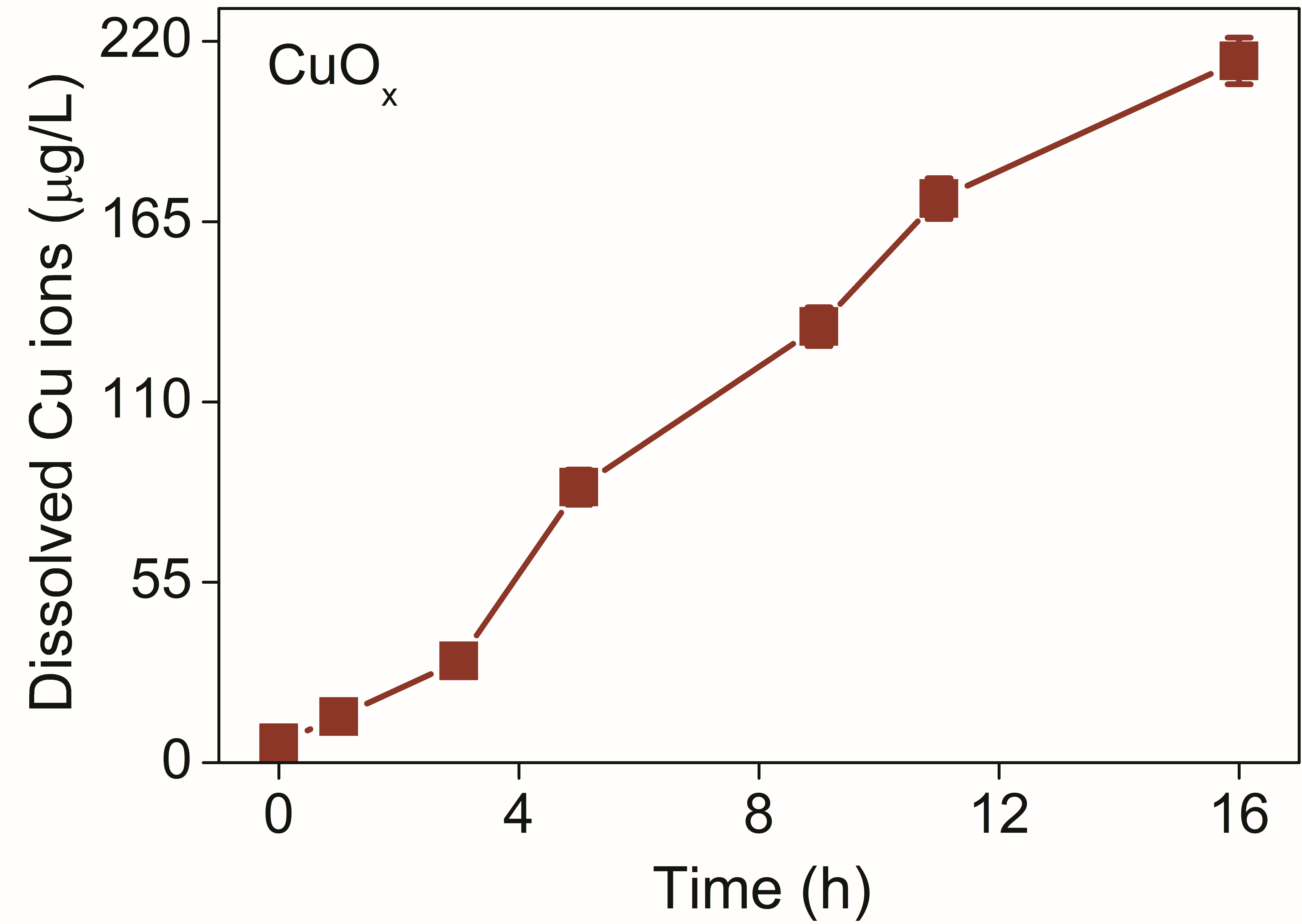


**Figure S4.** The amount of copper ions leaching from CuO_x_ during a long-time operation. Experimental conditions: at −0.1 V vs. RHE under AM 1.5G simulated sunlight (100 mW/cm^2^) using 0.1 M KHCO_3_ as electrolyte (CO_2_-saturated). Data are presented as mean ± s.d. (*n* = 3 independent chemical replicates) in d.


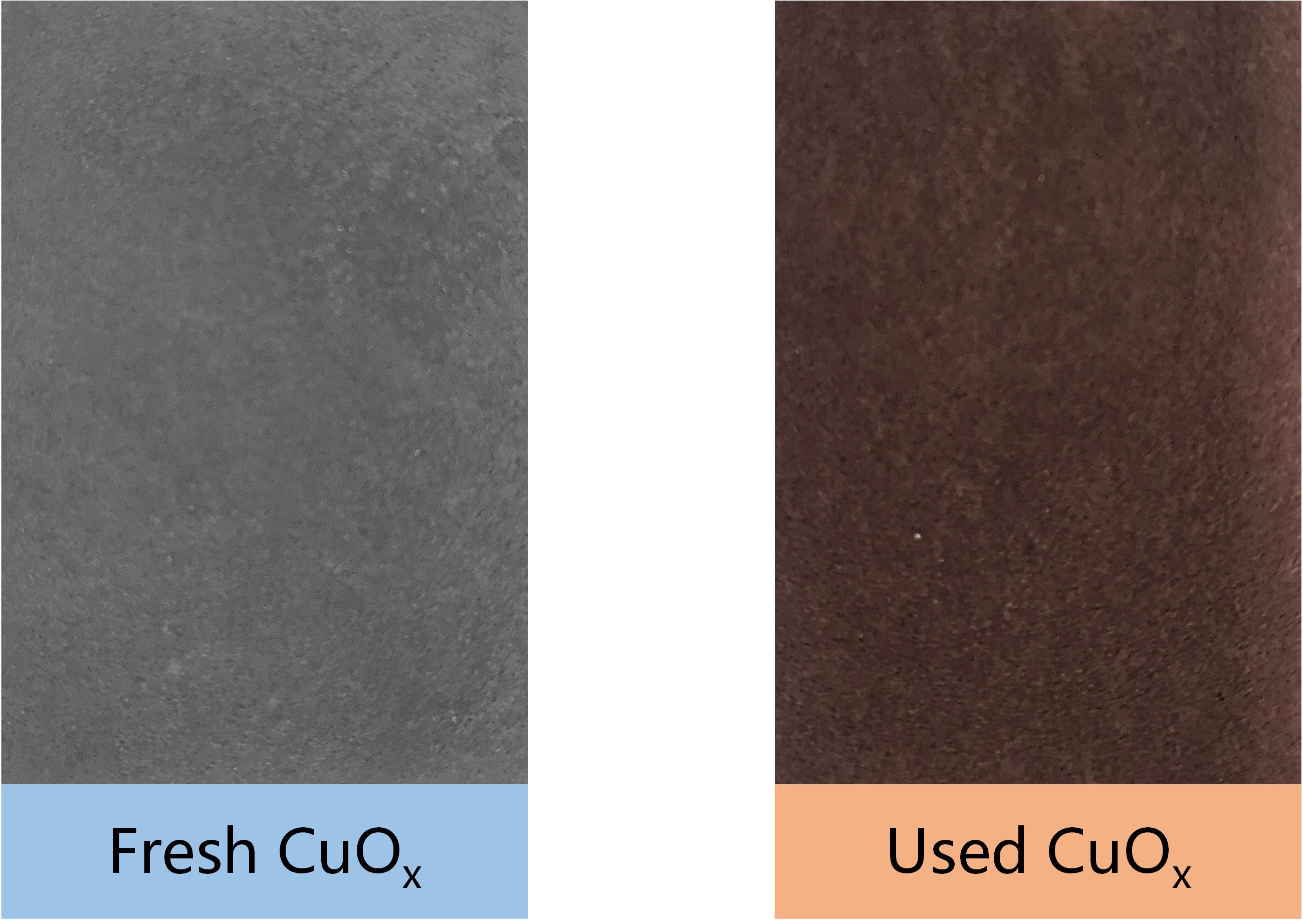


**Figure S5.** The photographs of fresh and used CuO_x_.


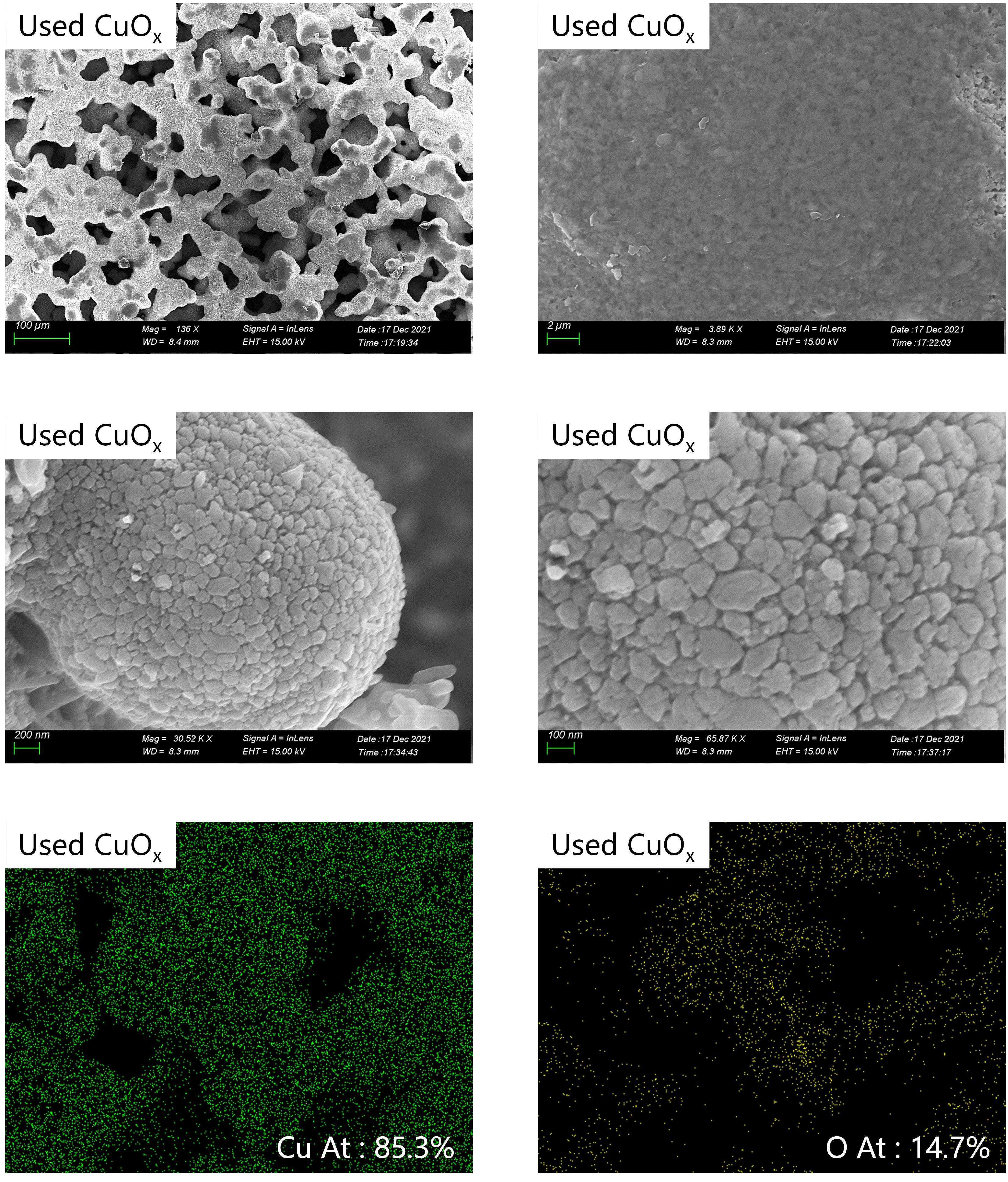


**Figure S6.** The SEM and EDX-mapping images of used CuO_x_.


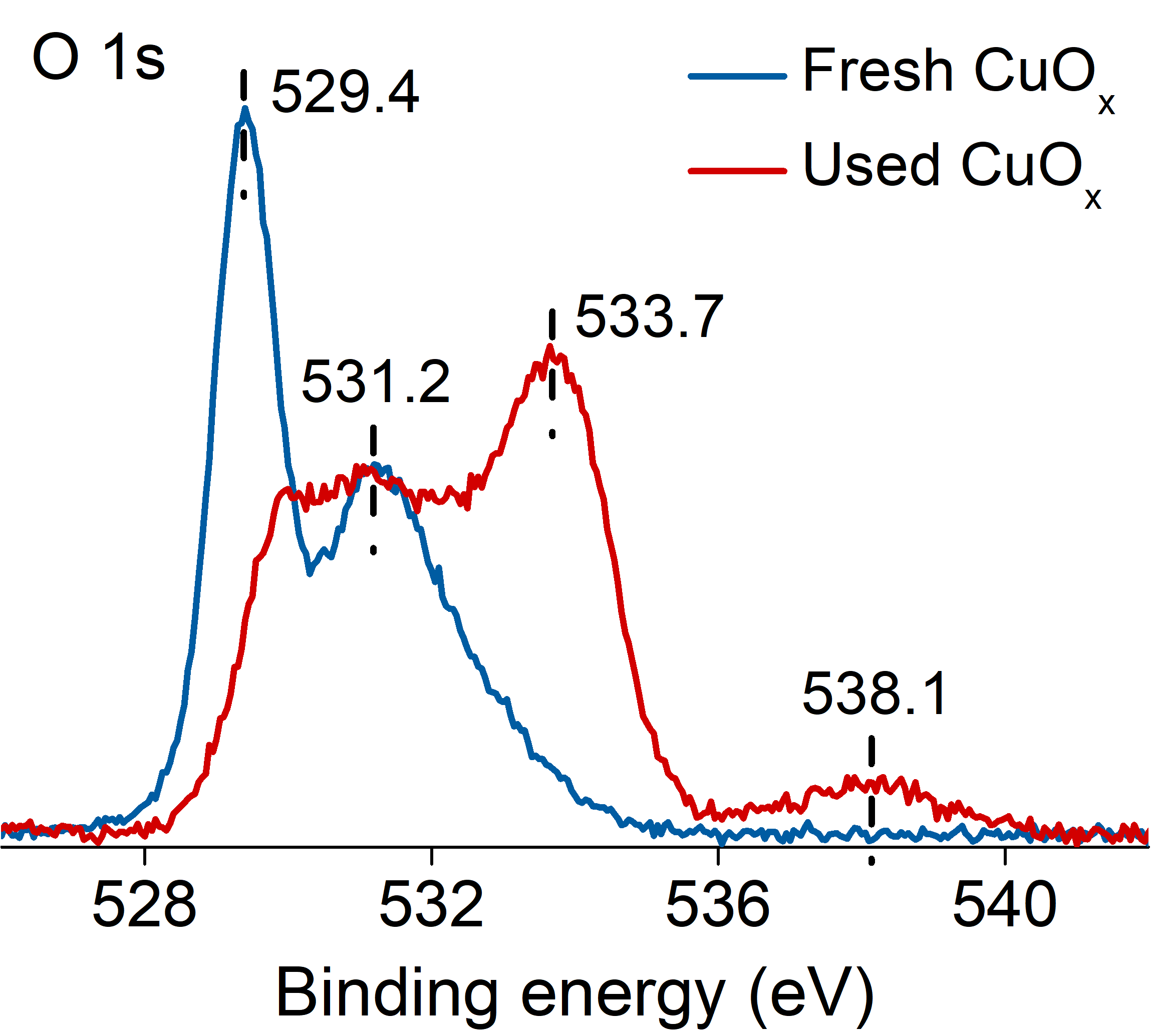


**Figure S7.** The high-resolution XPS O 1s spectra of fresh and used CuO_x_.


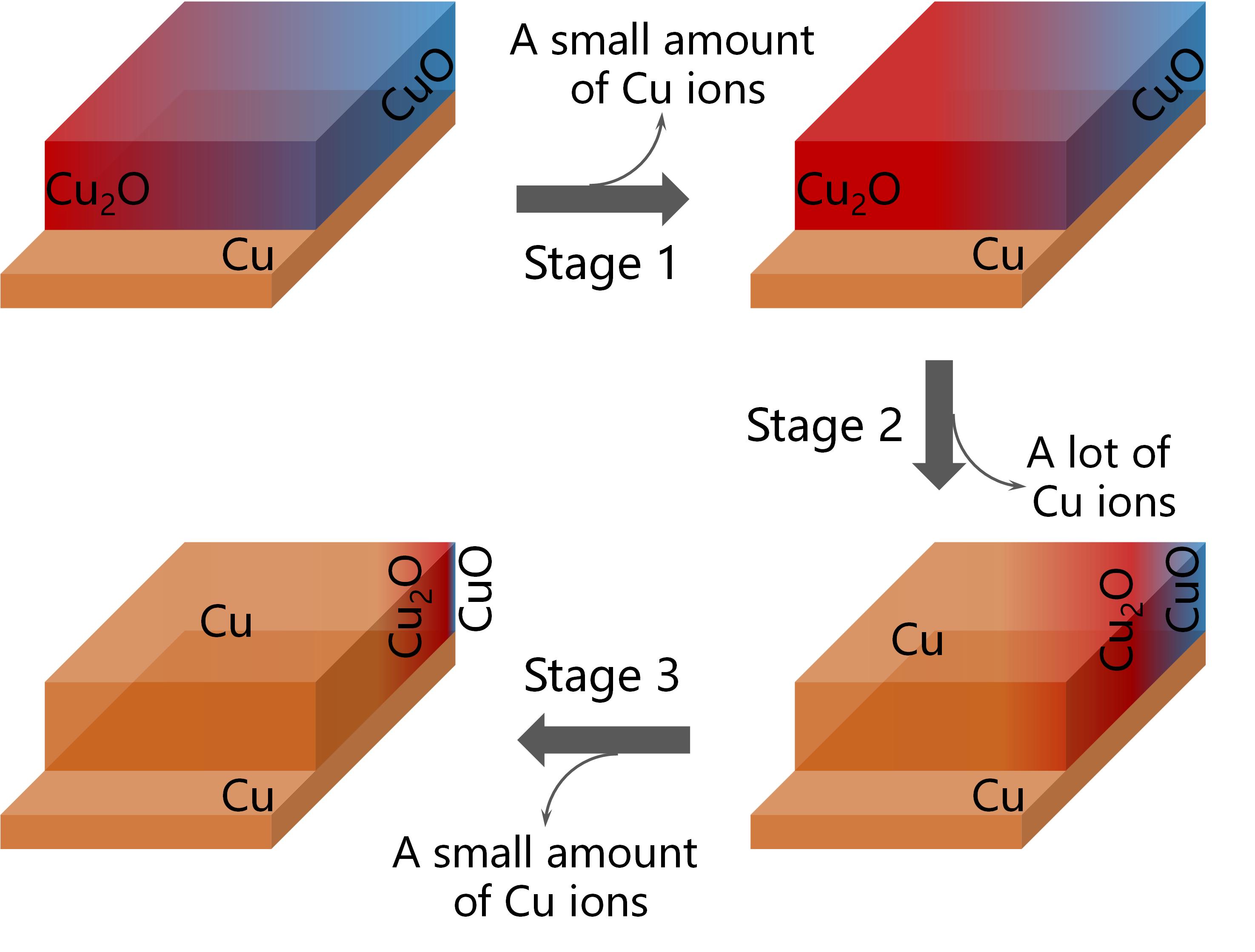


**Figure S8.** Schematic diagram of degradation process and mechanism of CuO_x_.


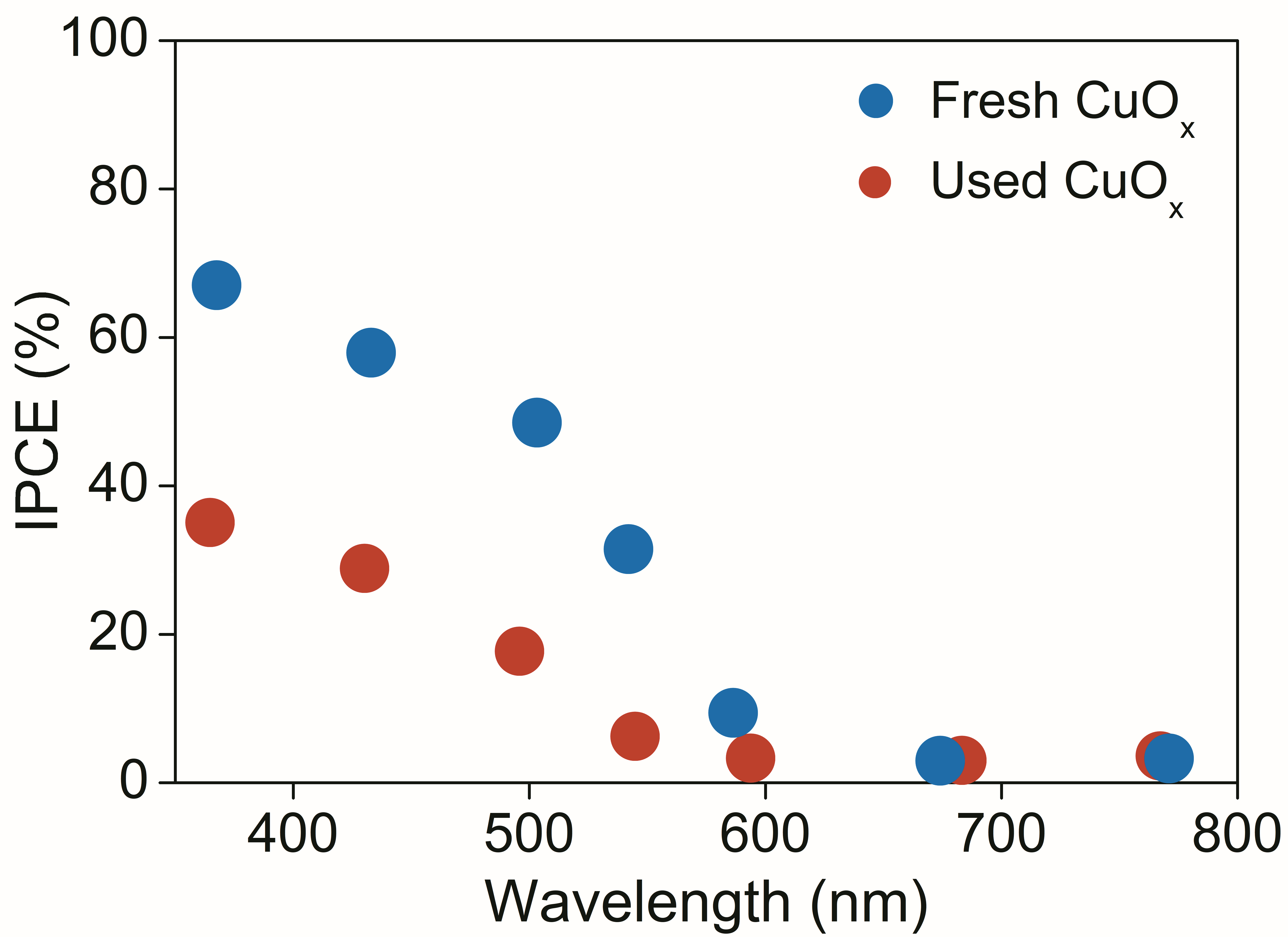


**Figure S9.** The IPCE of the fresh and used CuO_x_.


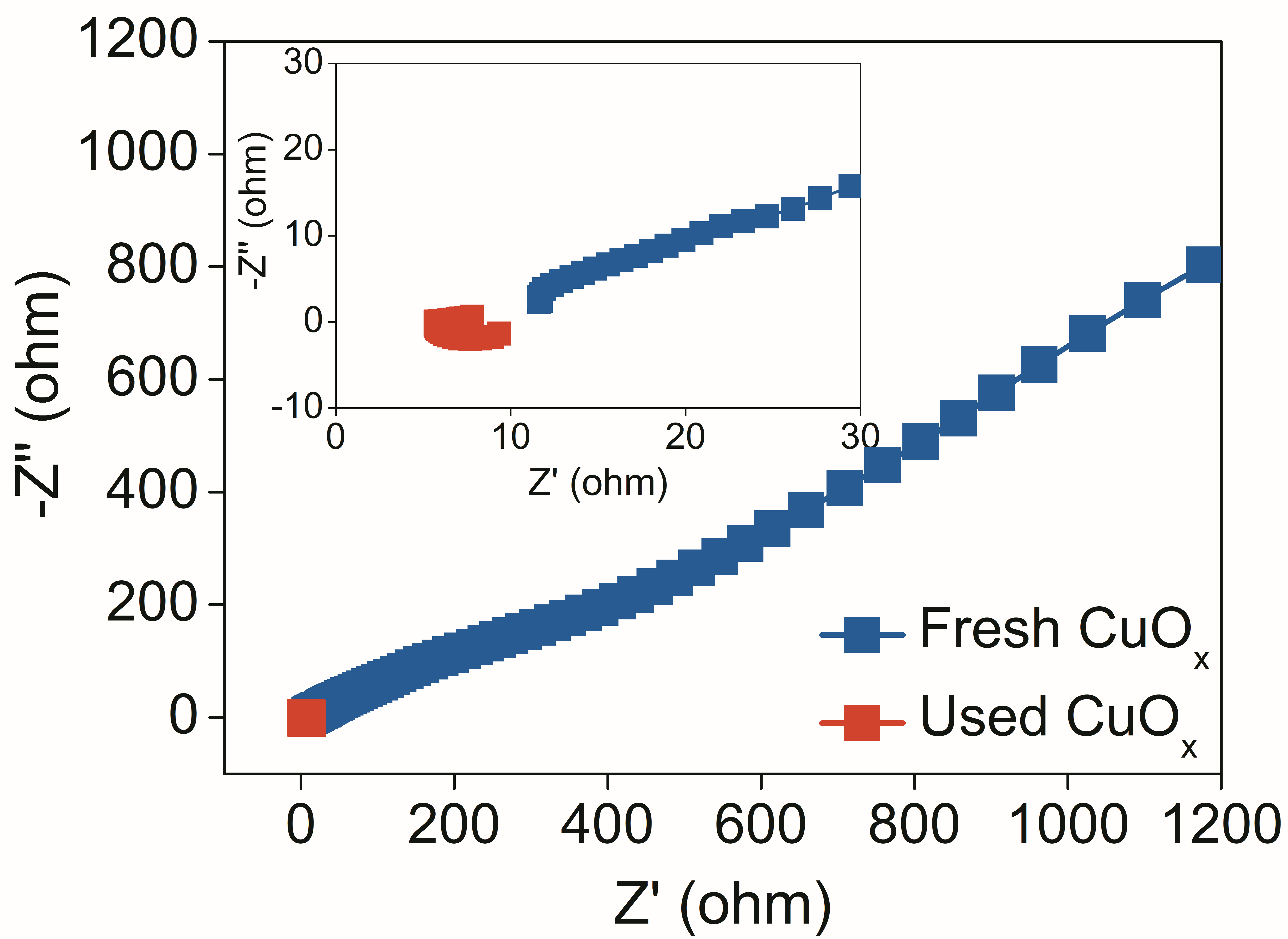


**Figure S10.** The electrochemical impedance of fresh and used CuO_x_. Experimental conditions: at −0.1 V vs. RHE under AM 1.5G simulated sunlight (100 mW/cm^2^) using 0.1 M KHCO_3_ as electrolyte (CO_2_-saturated).


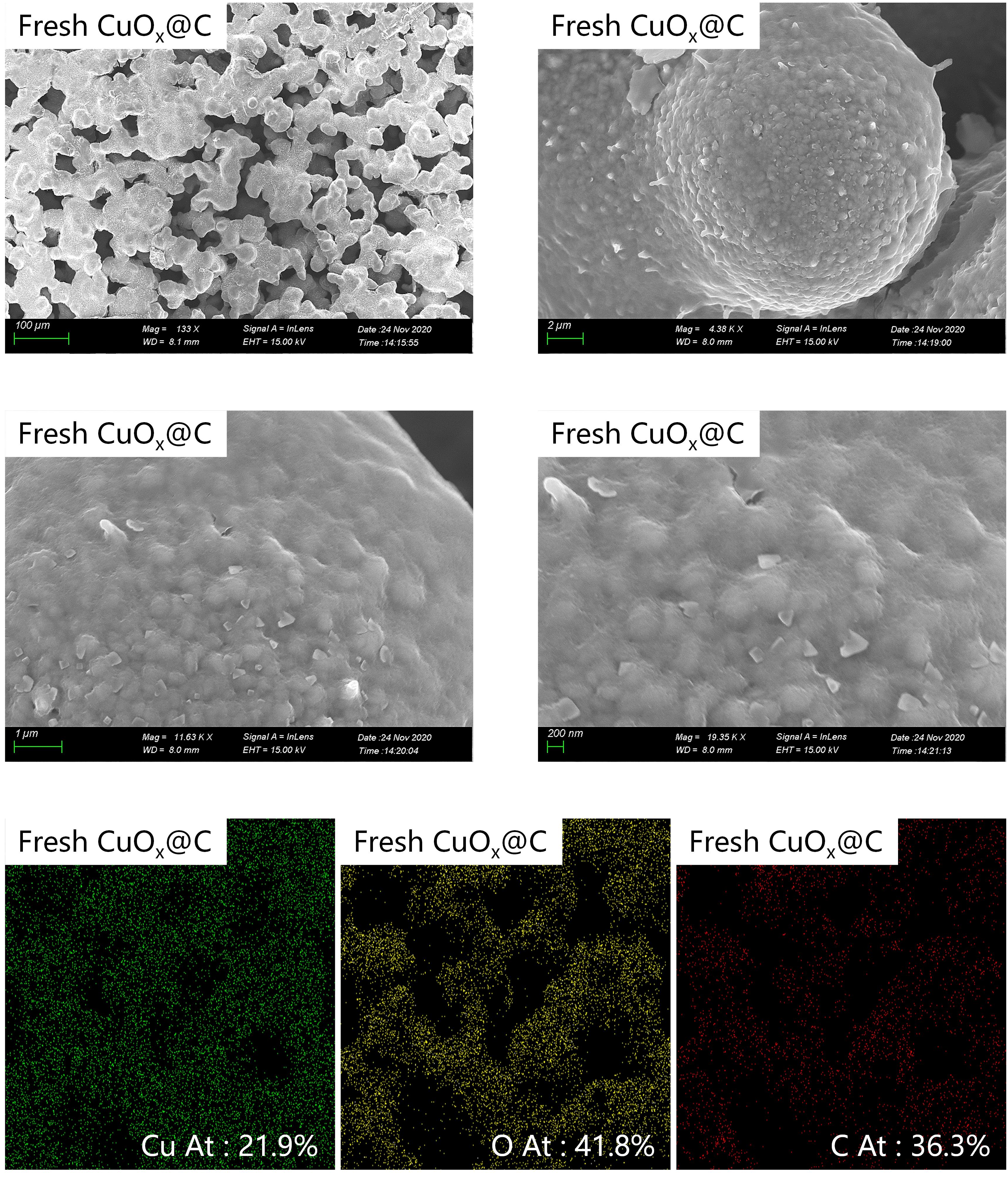


**Figure S11.** The SEM and EDX-mapping images of fresh CuO_x_@C.


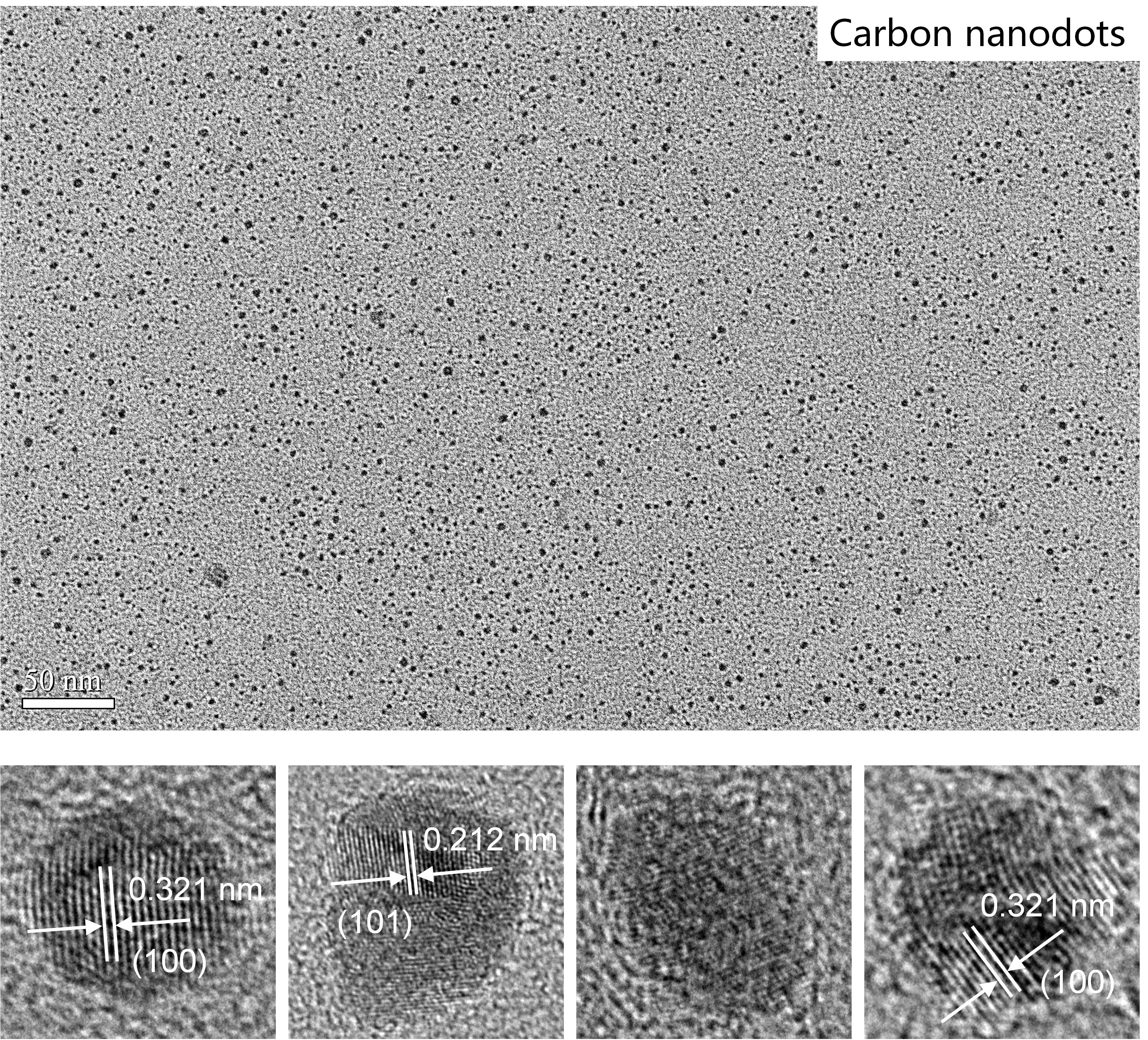


**Figure S12.** The TEM images of carbon nanodots.


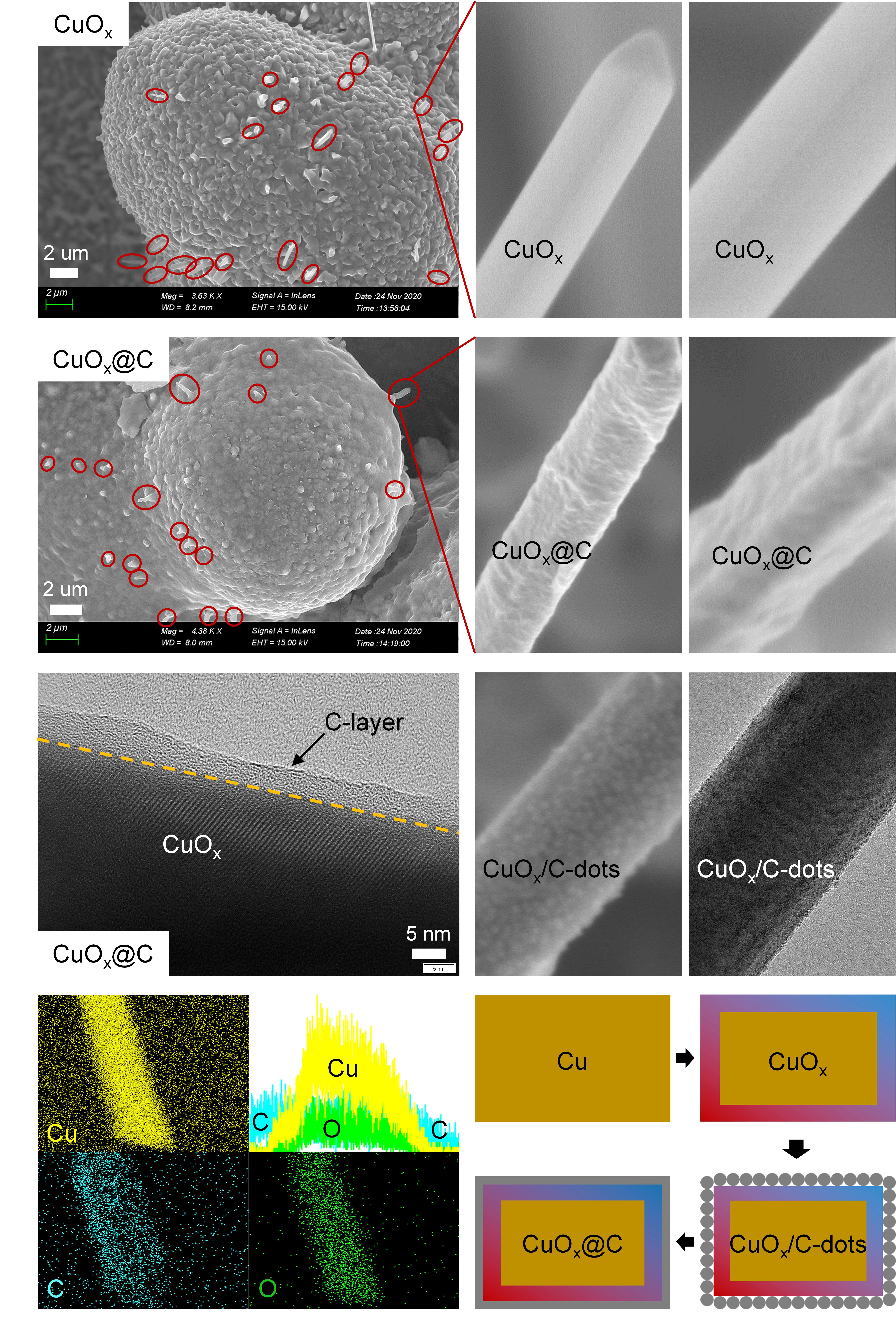


**Figure S13.** The TEM and EDX-mapping images of fresh CuO_x_ and CuO_x_@C.

Comprehensive electron microscopy analysis provides definitive evidence for the successful construction of a conformal carbon coating on CuO_x_ via an electrodeposition-coupled self-assembly mechanism (**Figure S13**). SEM imaging reveals that the pristine CuO_x_ exhibits a characteristic morphology comprised of irregular Cu_2_O/CuO heterojunction particles with a smooth surface. Upon carbon functionalization, the CuO_x_@C sample maintains the underlying particle topography but reveals complete encapsulation by a continuous carbon overlayer, a feature conspicuously evident even at prominent surface protrusions where coating integrity is most challenged. High-resolution TEM imaging focused on these critical prominent positions resolves an ultrathin, dense carbon layer adhering conformally to the CuO_x_ substrate with a remarkably uniform thickness of ~6 nm. This core-shell architecture is unambiguously confirmed by EDX-mapping, which visually demonstrates a continuous carbon signal fully encompassing the copper and oxygen domains. The observed thickness of carbon overlayer was highly consistent across the material and commensurate with the nominal diameter of an individual carbon nanodot, which constitutes compelling evidence that the coating forms via a monolayer self-assembly process rather than random aggregation. To deconvolve the dynamic formation pathway of carbon layer, we systematically tracked intermediate synthesis stages using SEM and TEM. Initial electrodeposition leads to the dense, physical attachment of carbon nanodots onto the CuO_x_ surface. This is followed by a chemical bonding-driven self-assembly process that yields the final continuous and uniform carbon overlayer.


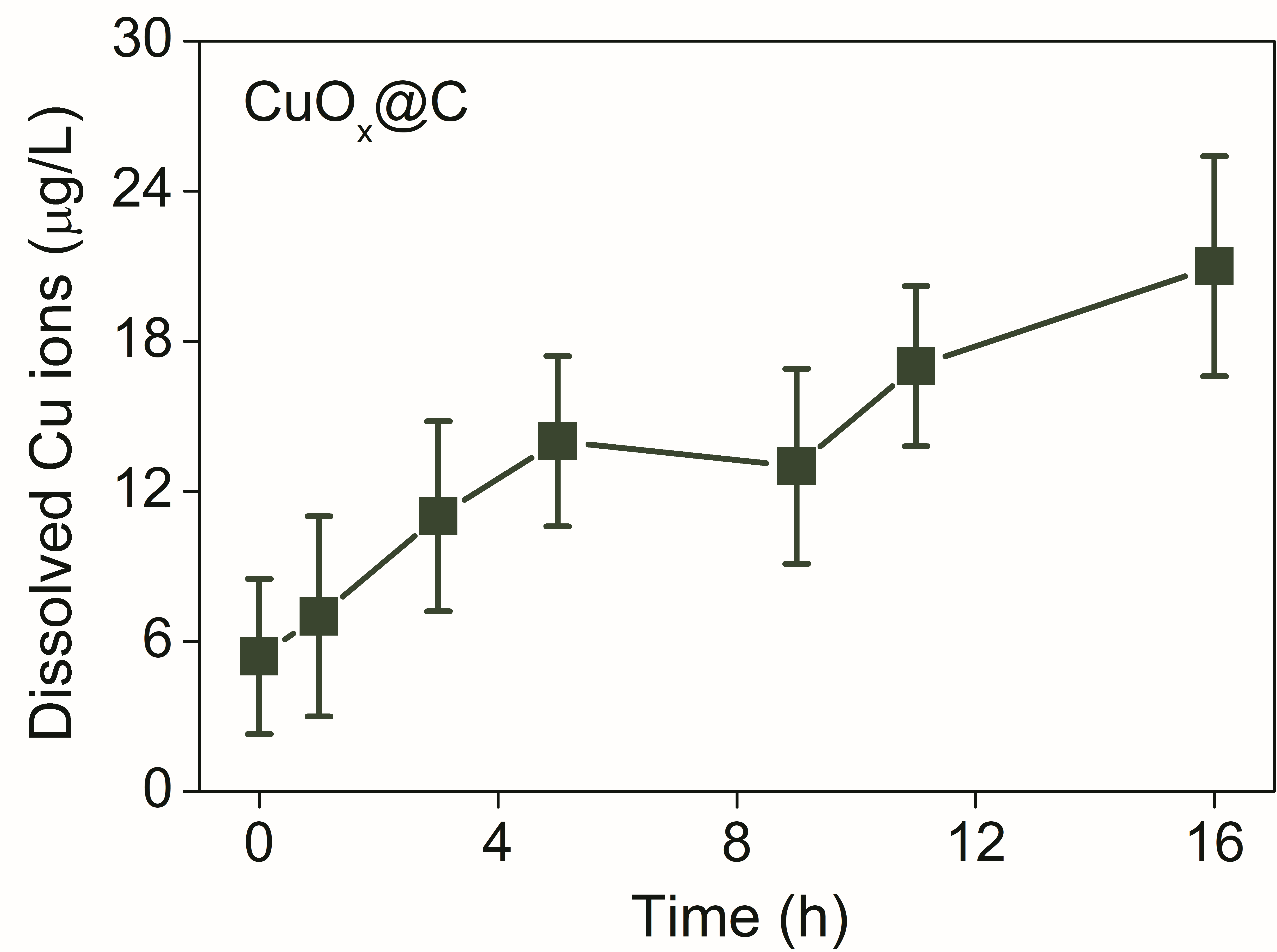


**Figure S14.** The amount of copper ions leaching from CuO_x_@C during a long-time operation. Experimental conditions: at −0.1 V vs. RHE under AM 1.5G simulated sunlight (100 mW/cm^2^) using 0.1 M KHCO_3_ as electrolyte (CO_2_-saturated). Data are presented as mean ± s.d. (*n* = 3 independent chemical replicates) in d.


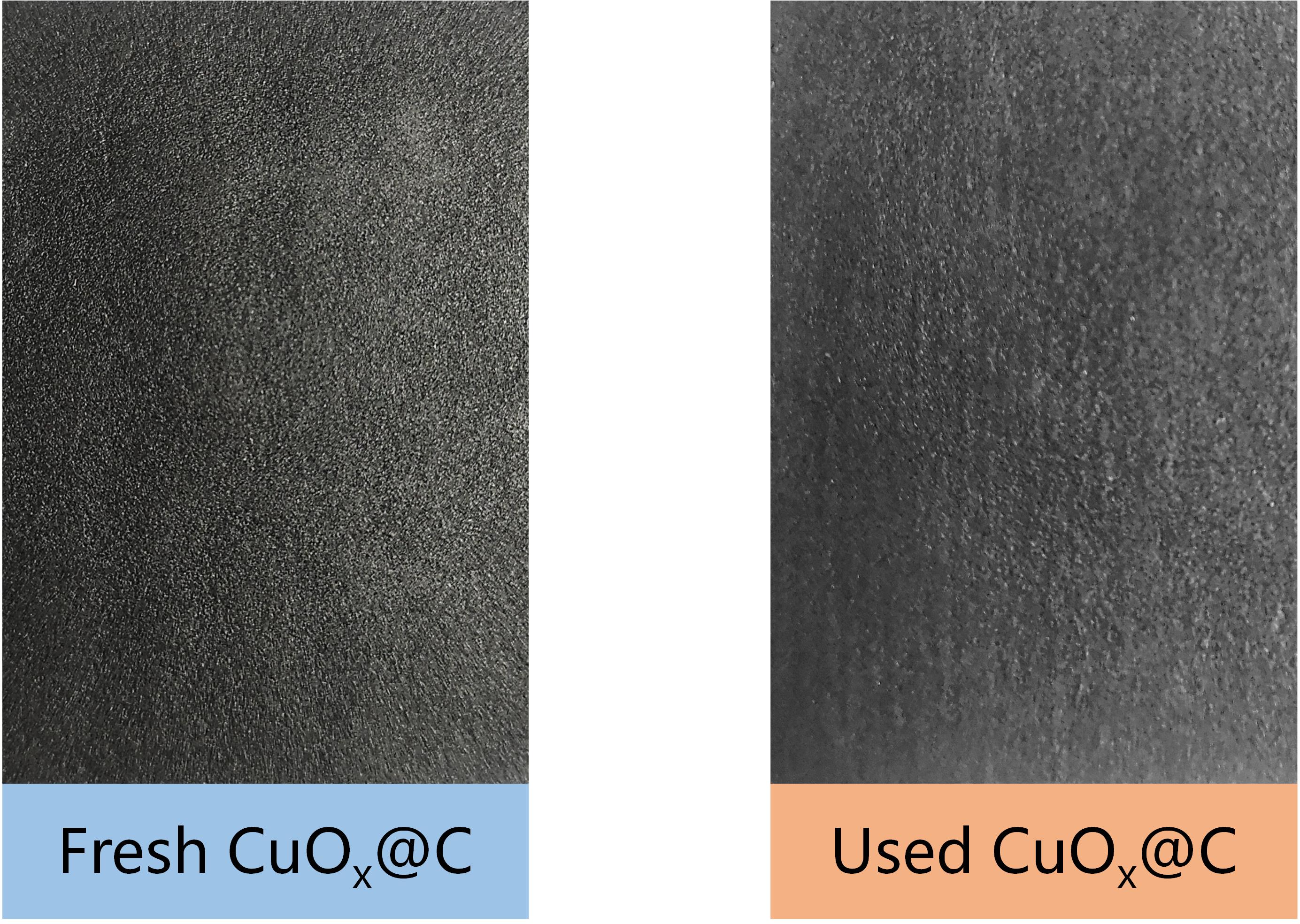


**Figure S15.** The photographs of fresh and used CuO_x_@C.


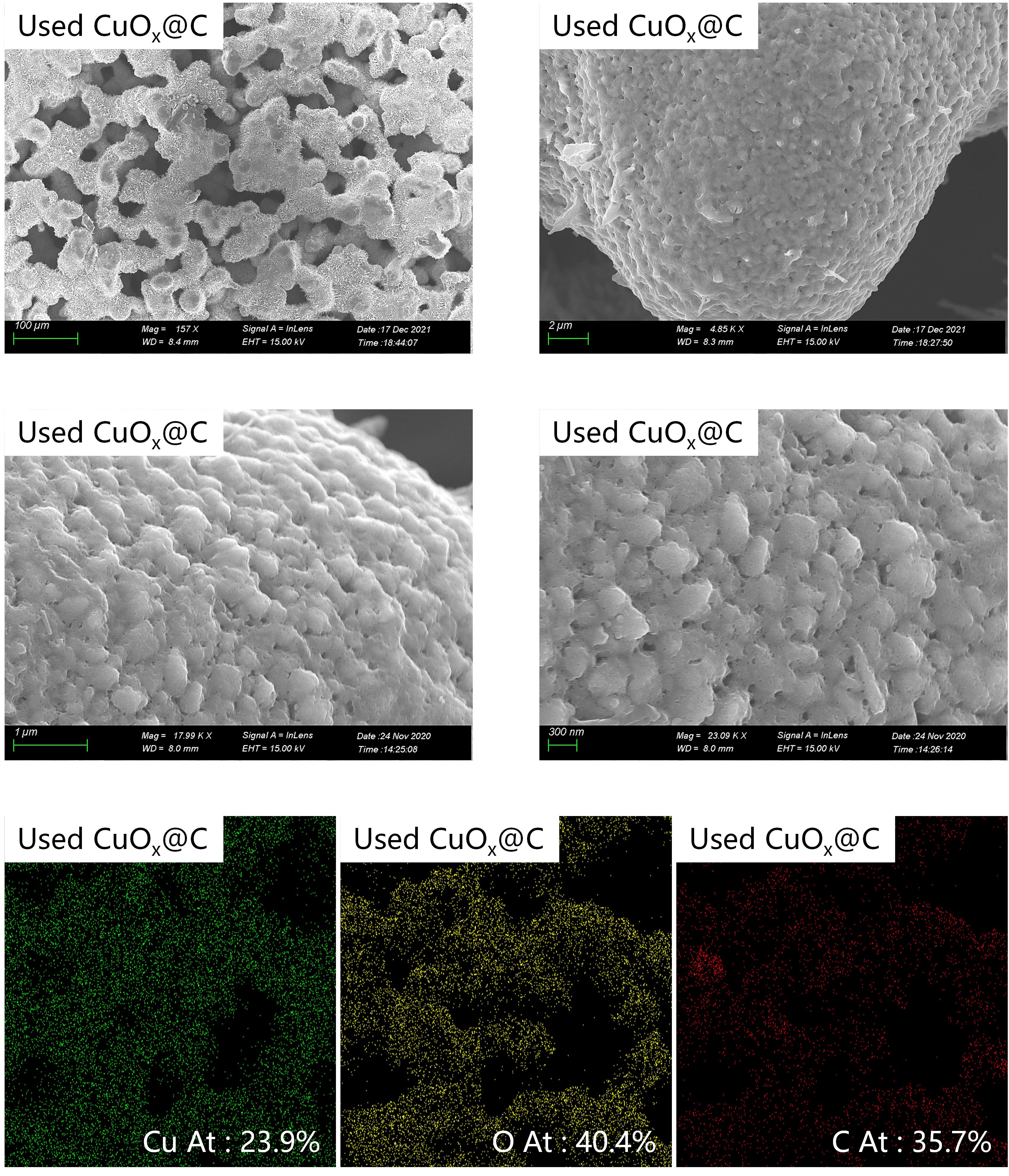


**Figure S16.** The SEM and EDX-mapping images of used CuO_x_@C.


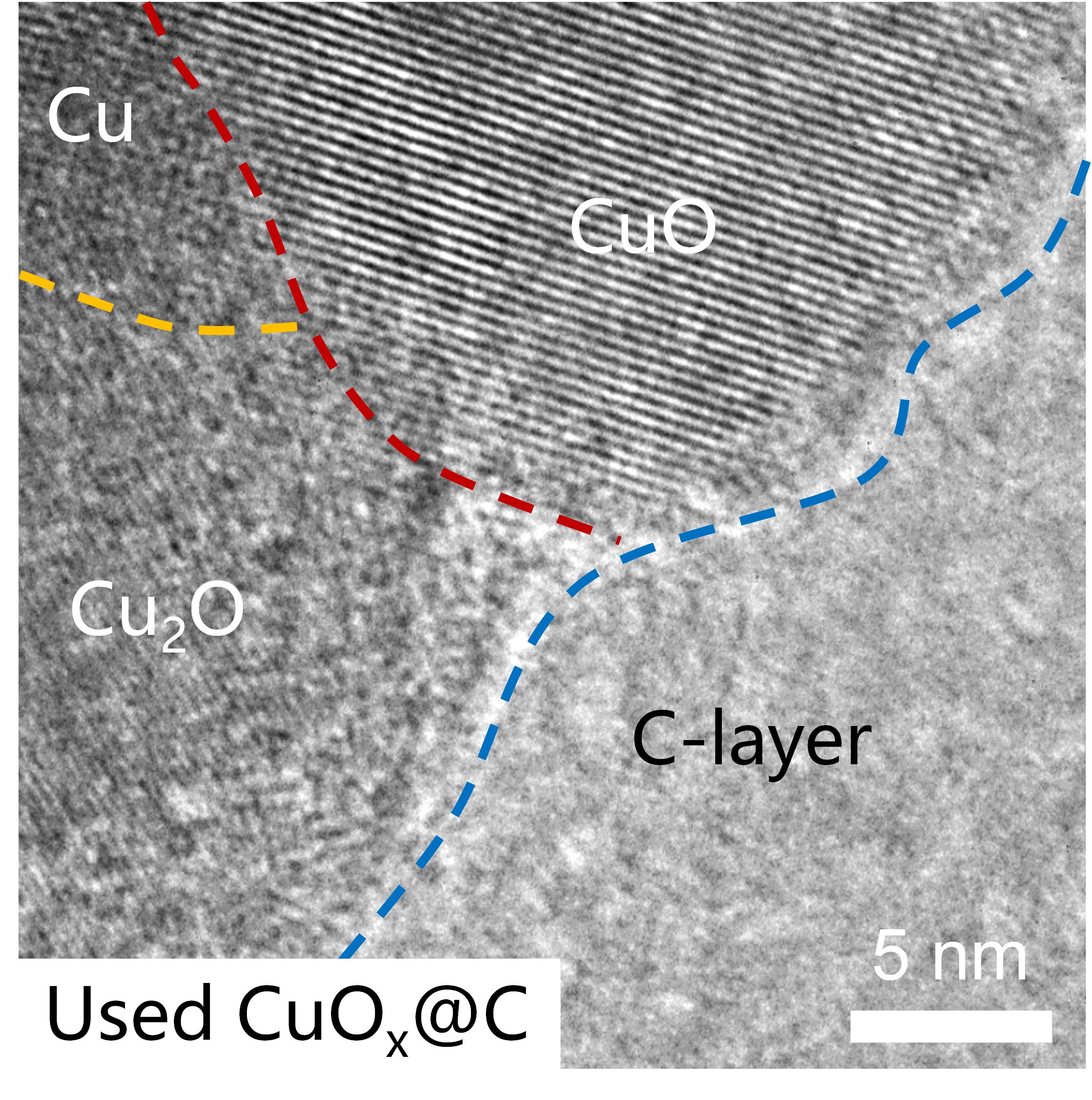


**Figure S17.** The TEM images of used CuO_x_@C.


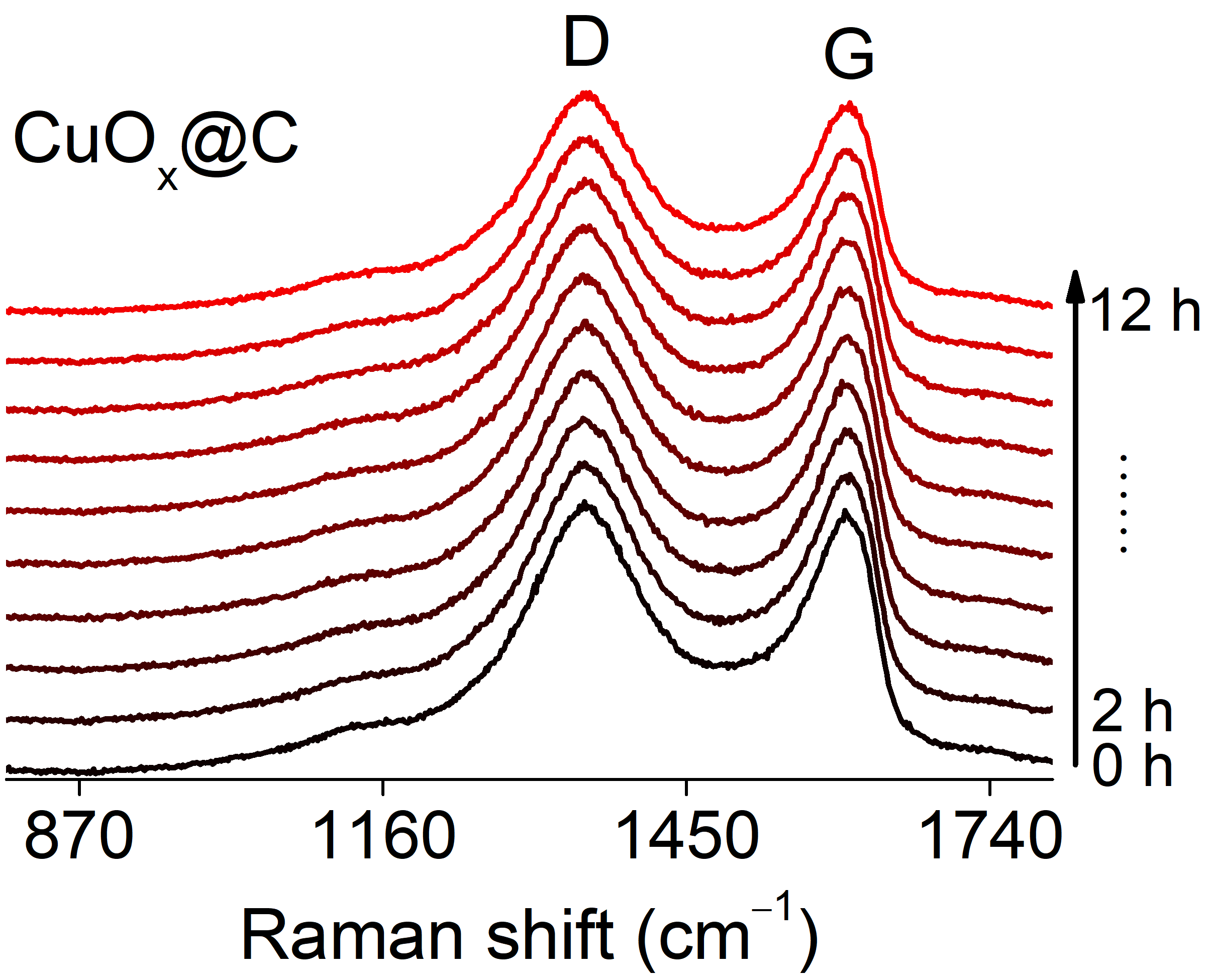


**Figure S18.** The in-situ Raman spectra of CuO_x_@C during a long-time operation. Experimental conditions: at −0.1 V vs. RHE under AM 1.5G simulated sunlight (100 mW/cm^2^) using 0.1 M KHCO_3_ as electrolyte (CO_2_-saturated).


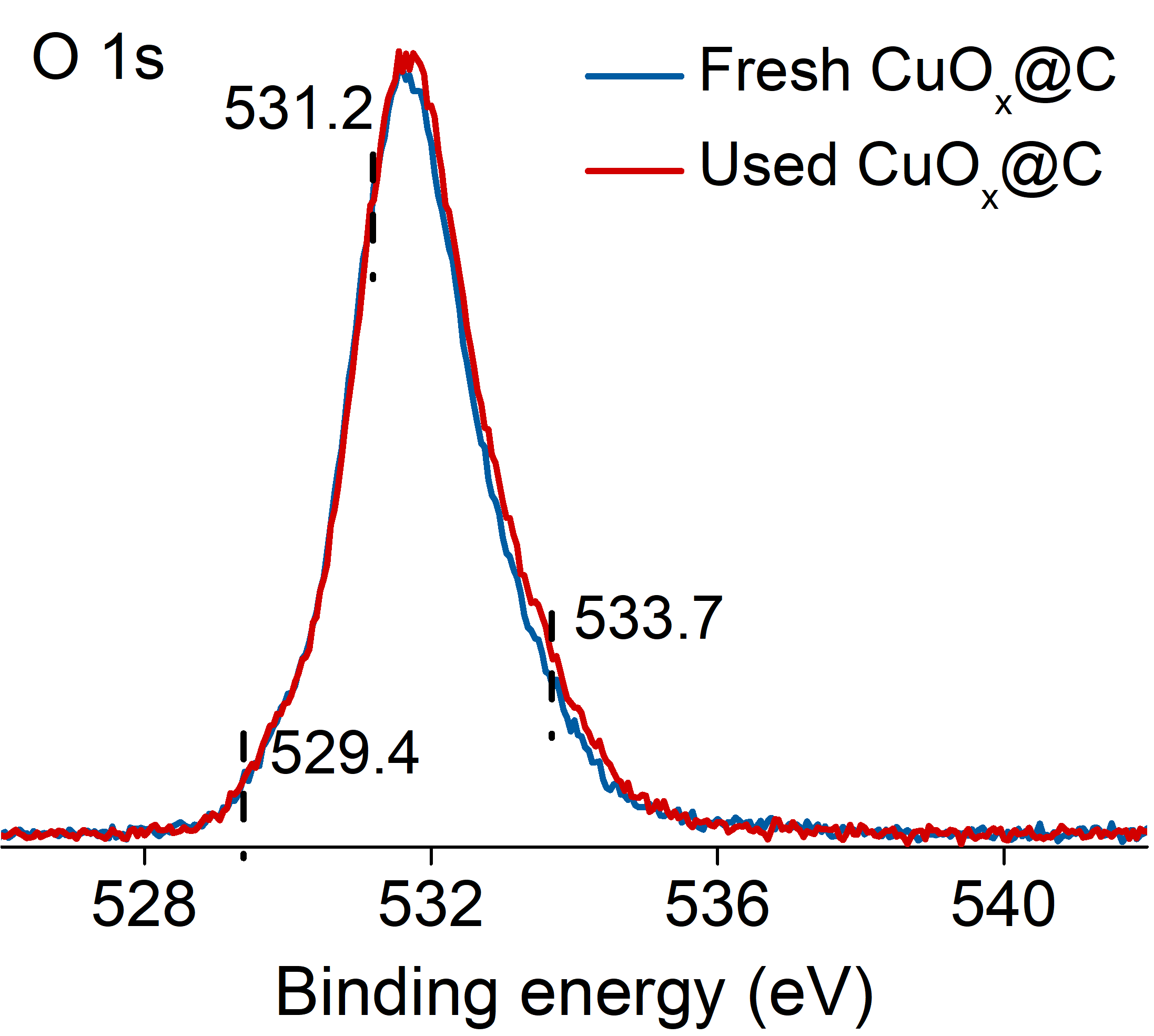


**Figure S19.** The high-resolution XPS O 1s spectra of fresh and used CuO_x_@C.


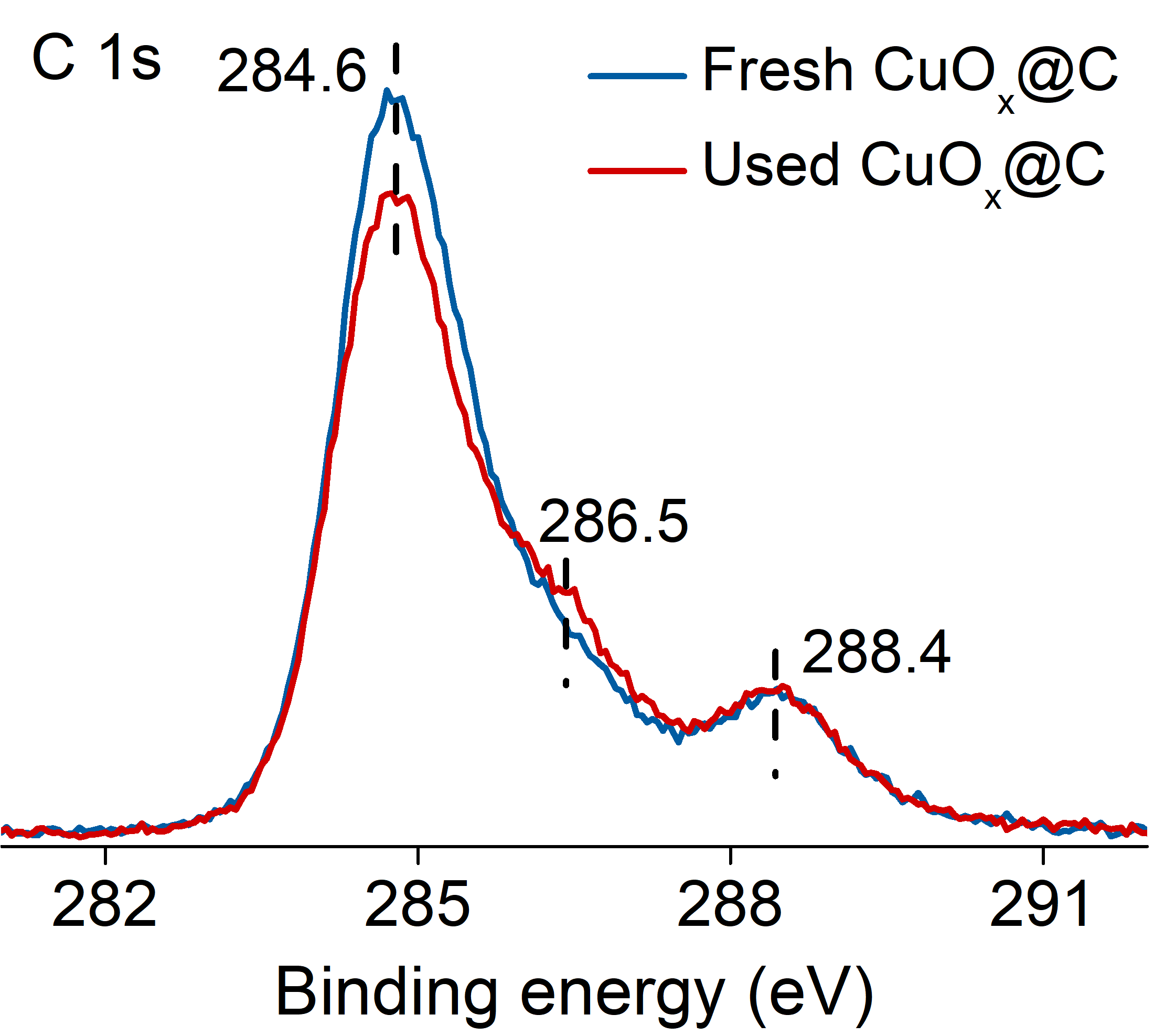


**Figure S20.** The high-resolution XPS C 1s spectra of fresh and used CuO_x_@C.


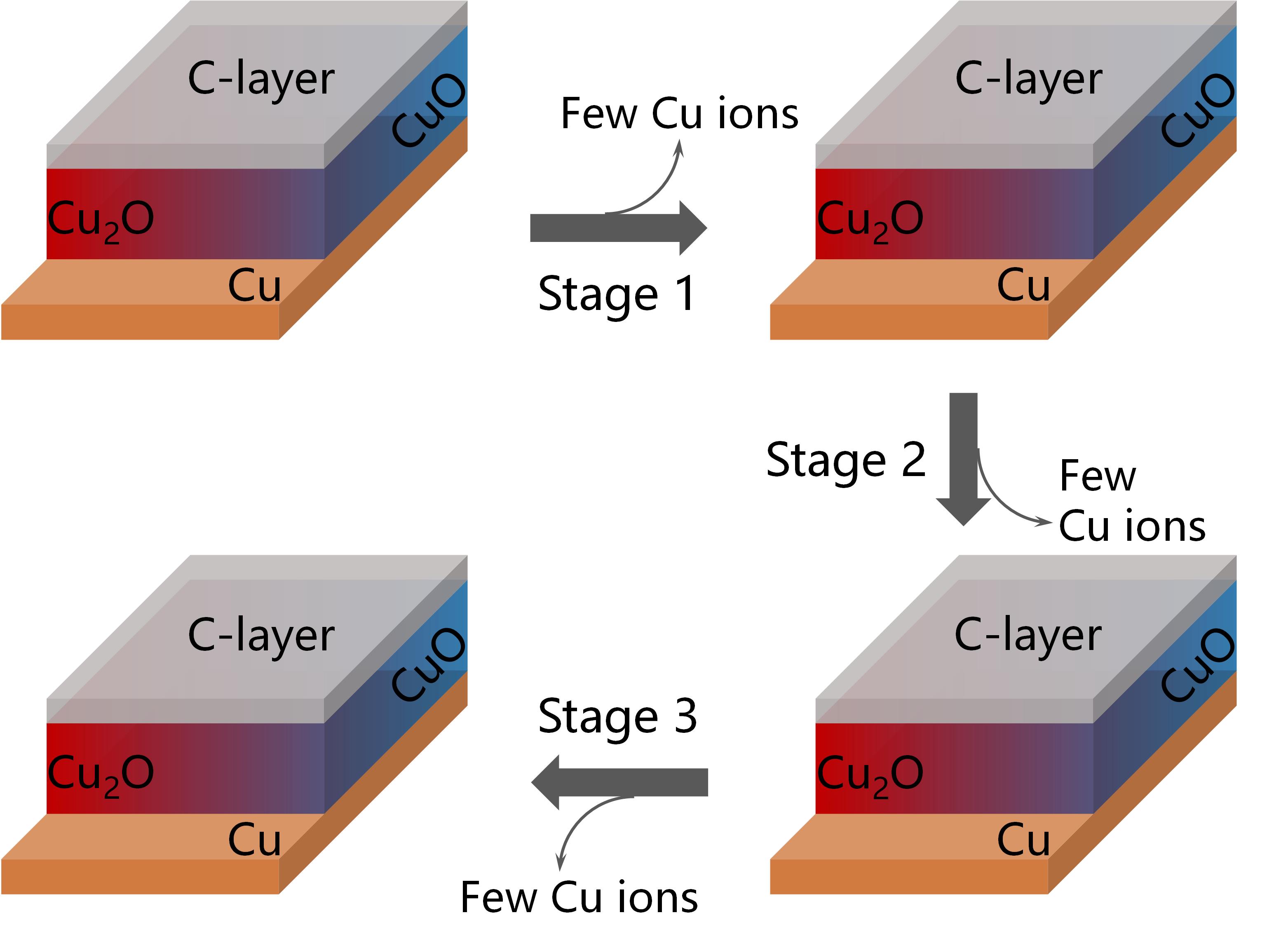


**Figure S21.** Schematic diagram of mitigating degradation process and mechanism of the CuO_x_@C photocathode.

**
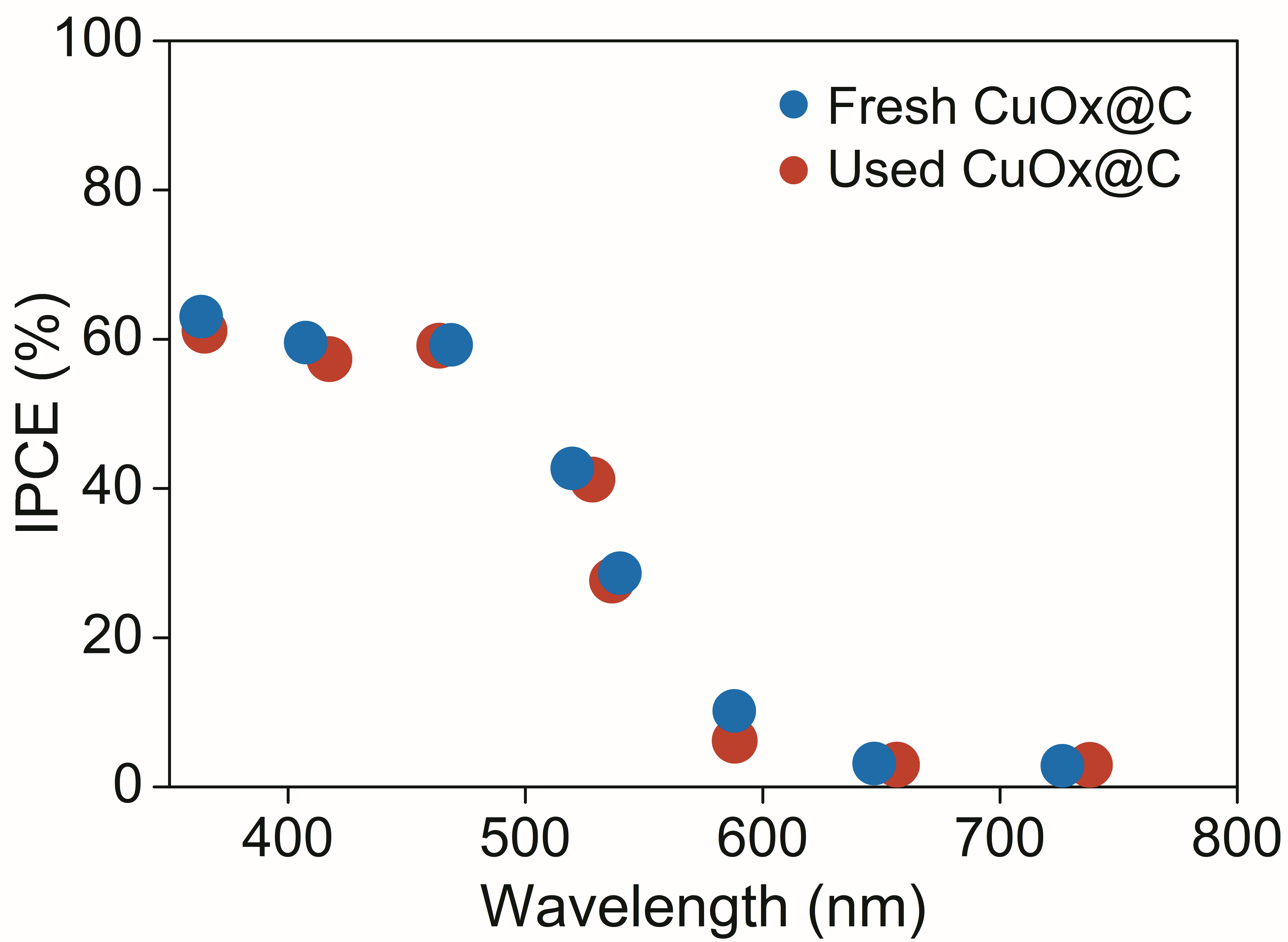
**

**Figure S22.** The IPCE of the fresh and used CuO_x_@C.


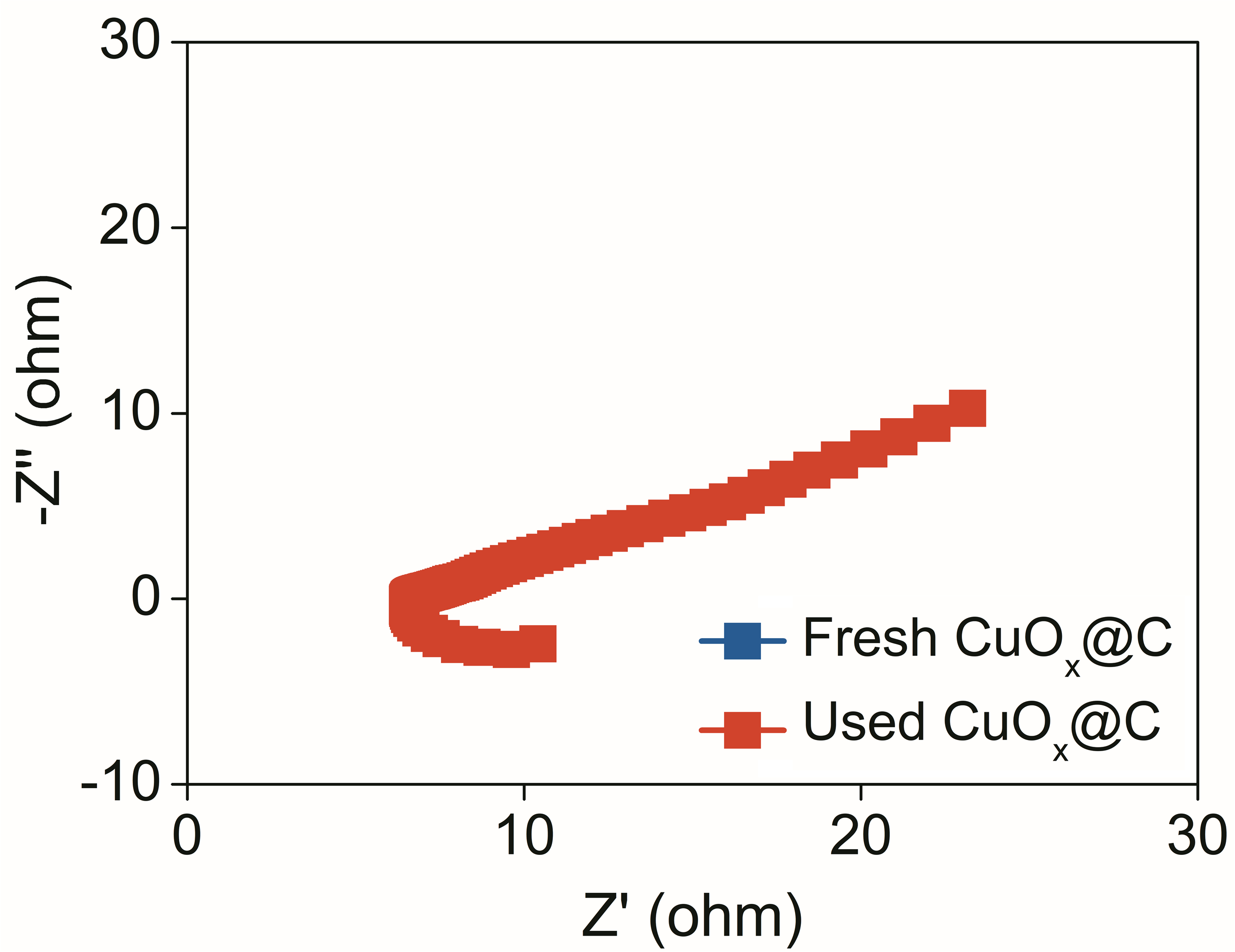


**Figure S23.** The electrochemical impedance of fresh and used CuO_x_@C. Experimental conditions: at −0.1 V vs. RHE under AM 1.5G simulated sunlight (100 mW/cm^2^) using 0.1 M KHCO_3_ as electrolyte (CO_2_-saturated).


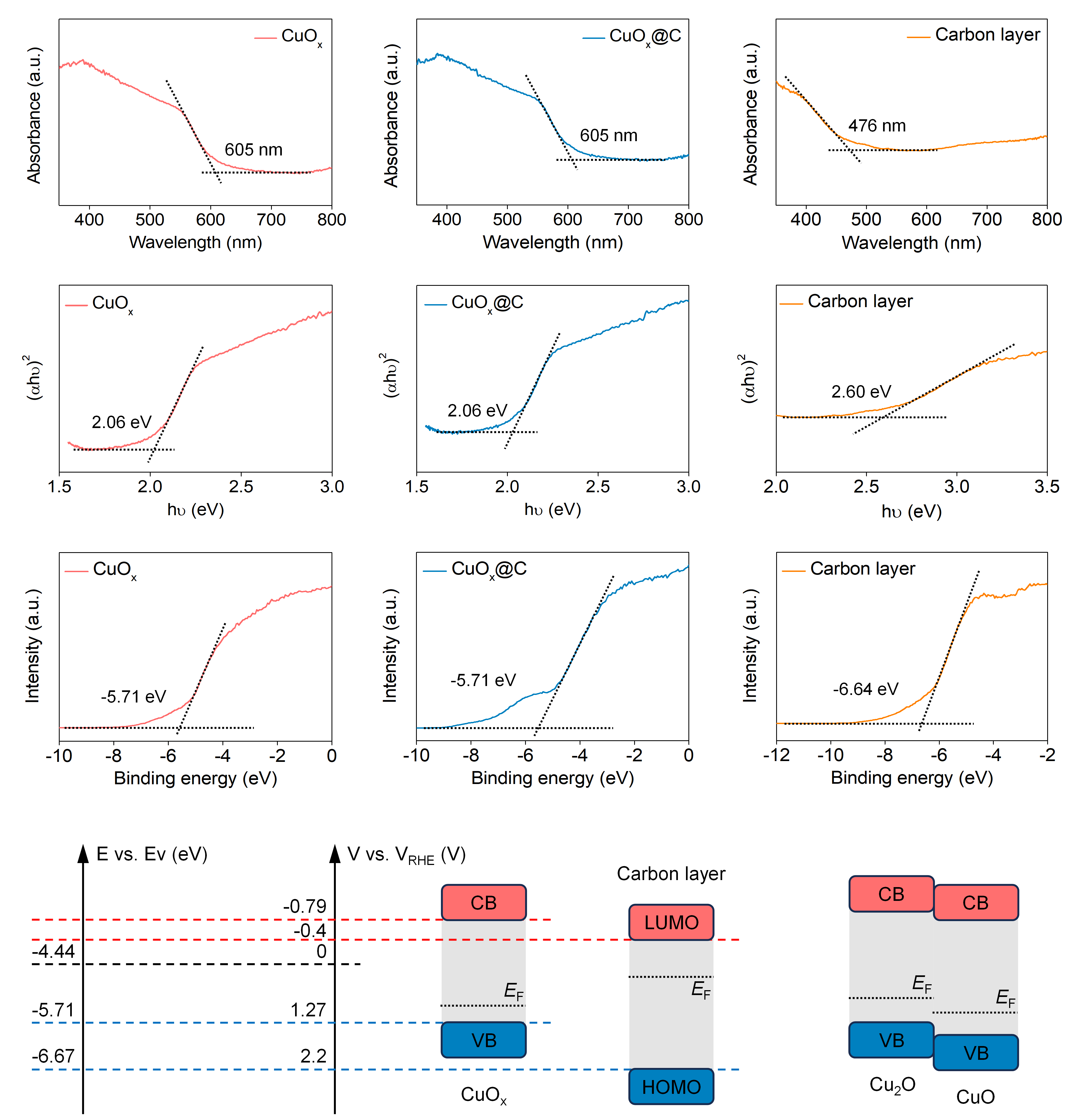


**Figure S24.** The analysis of the energy band structures of CuO_x_, carbon layer, and CuO_x_@C.


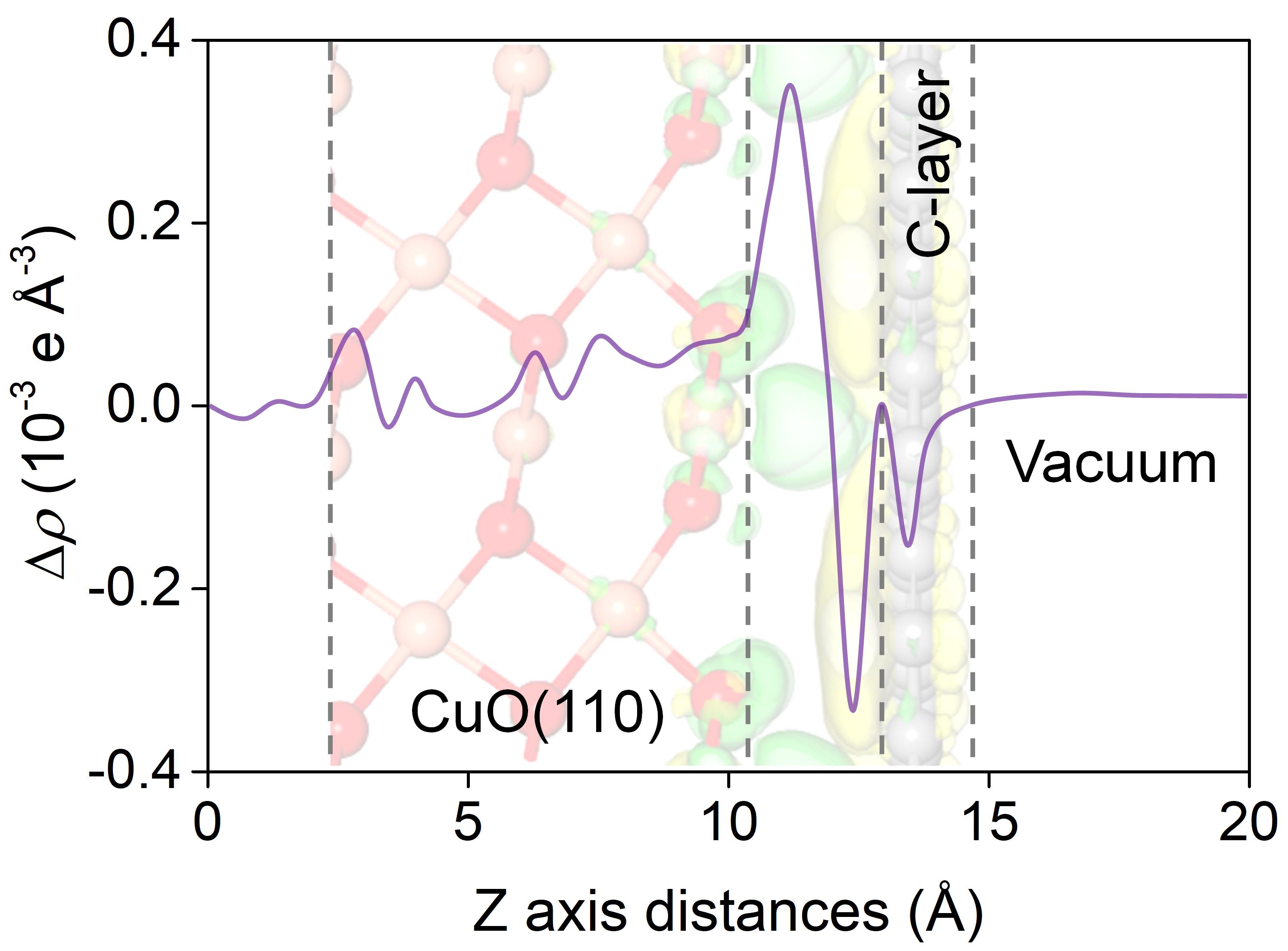


**Figure S25.** Planar-averaged charge density difference Δ*ρ* (Δ*ρ* = *ρ*_junction_ − *ρ*_CuO(110)_ − *ρ*_C_) along the *z*-direction for CuO(110)/graphite carbon(100).


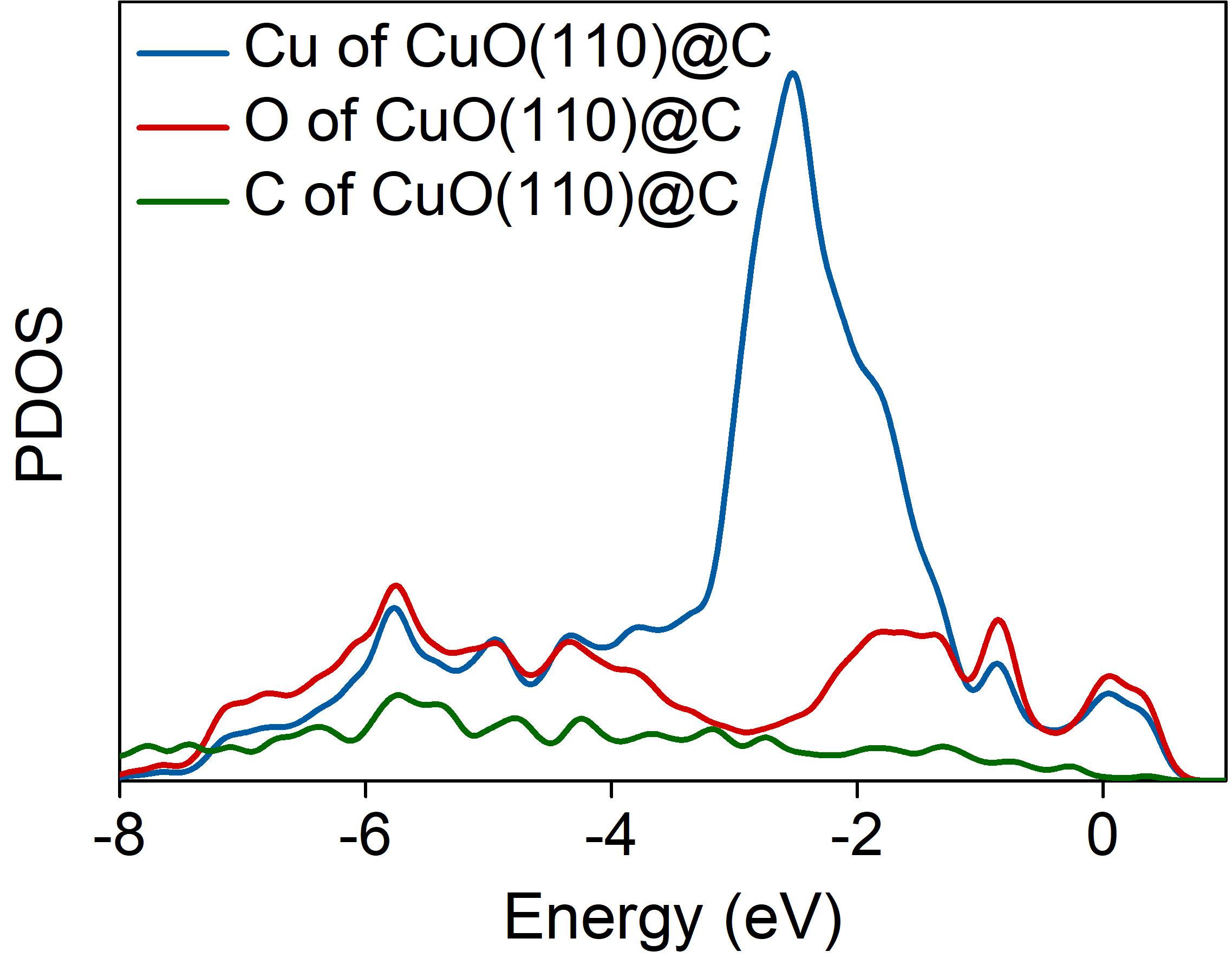


**Figure S26.** The PDOS of CuO(110)/graphite carbon(100).


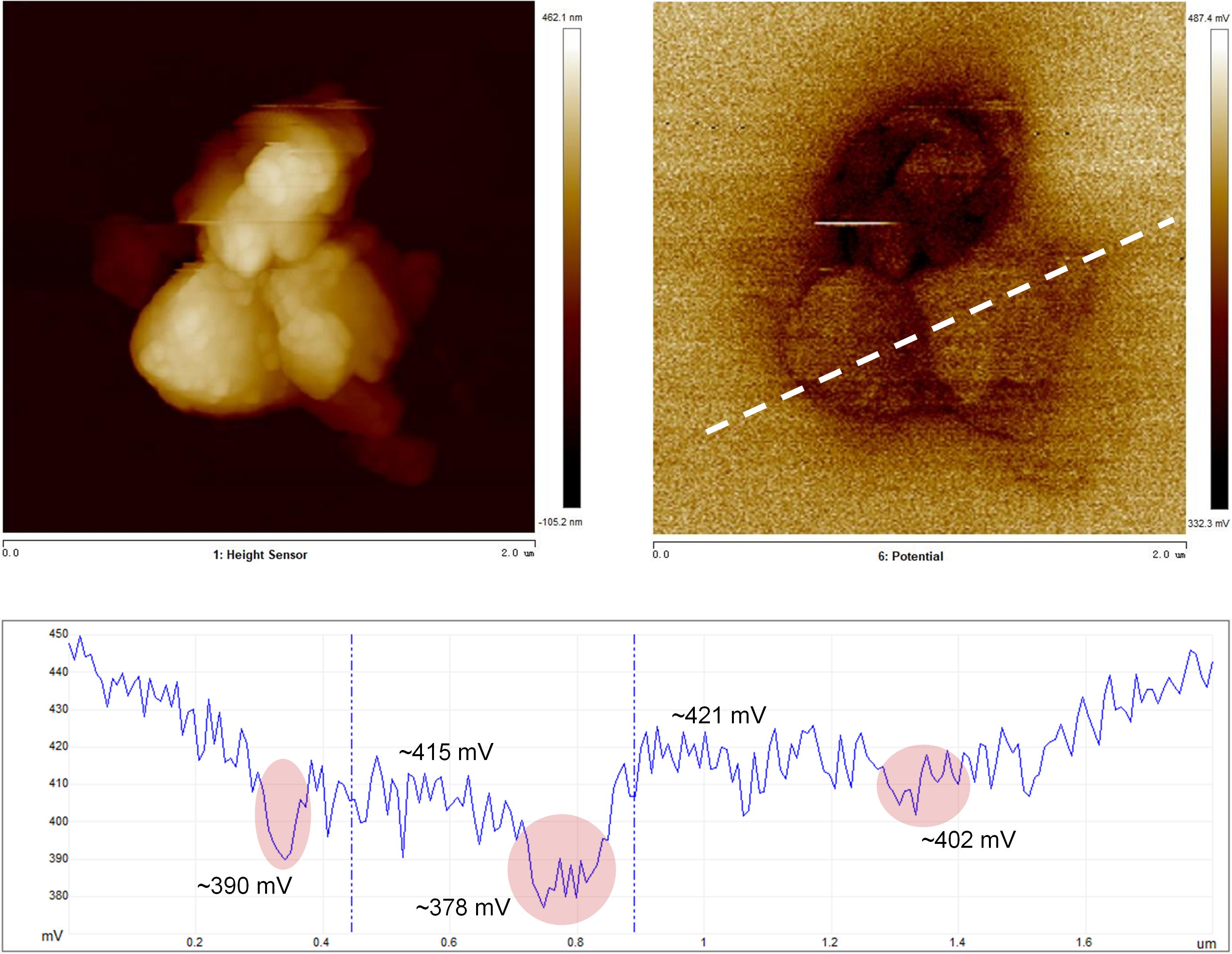


**Figure S27.** In-situ KPFM of CuO_x_@C under light irradiation.


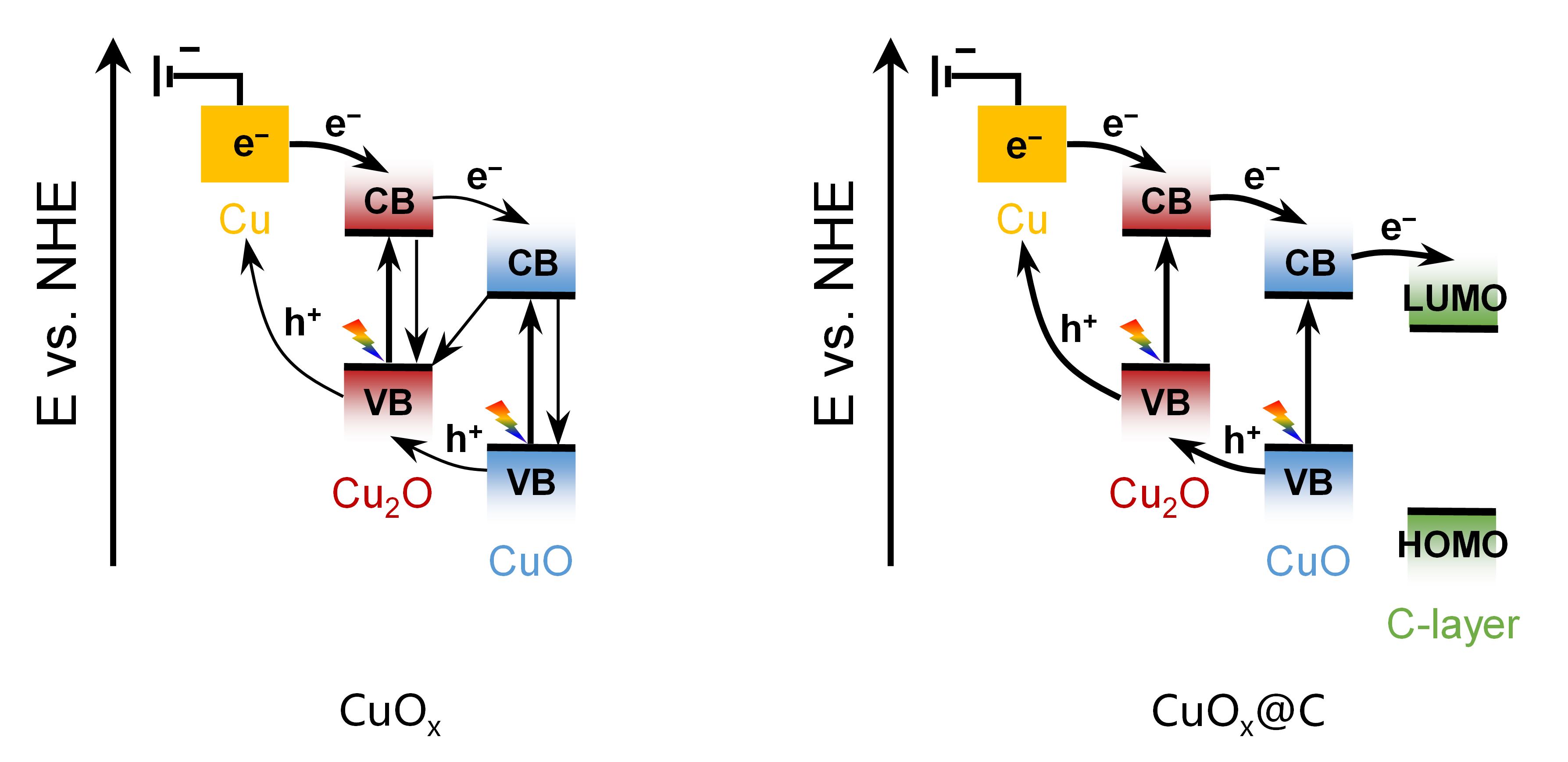


**Figure S28.** Schematic diagram of process and mechanism of photogenerated carriers' formation, recombination, migration and separation in CuO_x_ and CuO_x_@C.

The back contact in CuO_x_@C is the Cu substrate, which serves as the physical support for the entire Cu_2_O/CuO (CuO_x_) heterostructure, the electrical connection to the external circuit, and the ultimate collector for the photogenerated holes (**Figure S28**). The hole conduction function is ingeniously integrated into the Cu_2_O/CuO heterojunction itself. The built-in electric field at the Cu_2_O/CuO interface effectively separates the photogenerated charge carriers. This field drives the holes toward the Cu back contact through the Cu_2_O layer, which acts as a hole-transport pathway. Simultaneously, it drives the electrons toward the LUMO of carbon layer for the subsequent reduction reaction. Thus, the entire heterojunction structure, facilitated by the energy band alignment, performs the essential function of a hole conductor. In summary, the hole transport is managed by the designed heterojunction, while the Cu layer acts as the back contact.


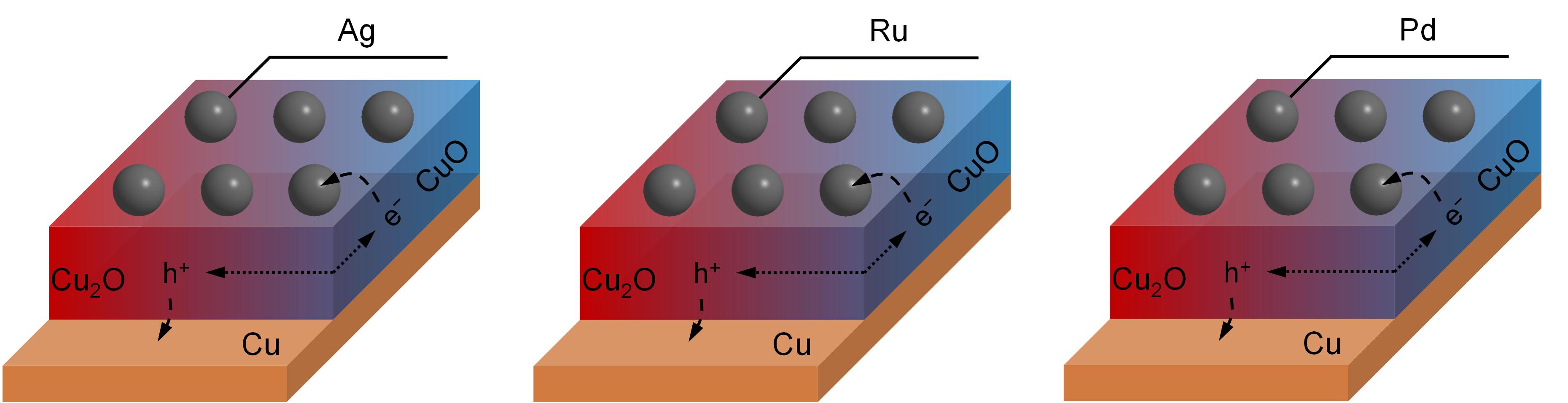


**Figure S29.** Schematic diagrams of migration and separation path of photogenerated carriers in CuO_x_/Ag, CuO_x_/Ru and CuO_x_/Pd.


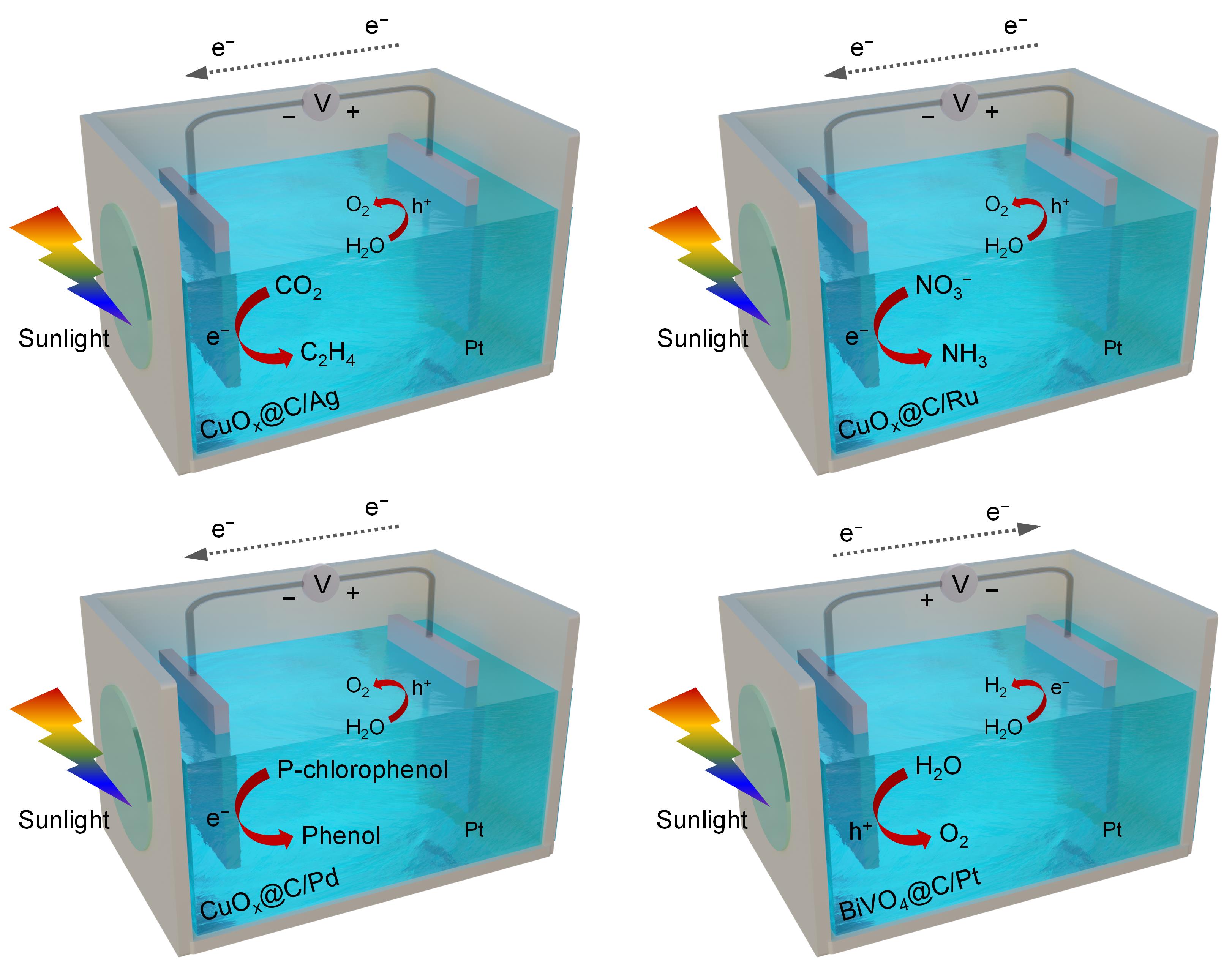


**Figure S30.** Rational design in selective catalytic conversion.


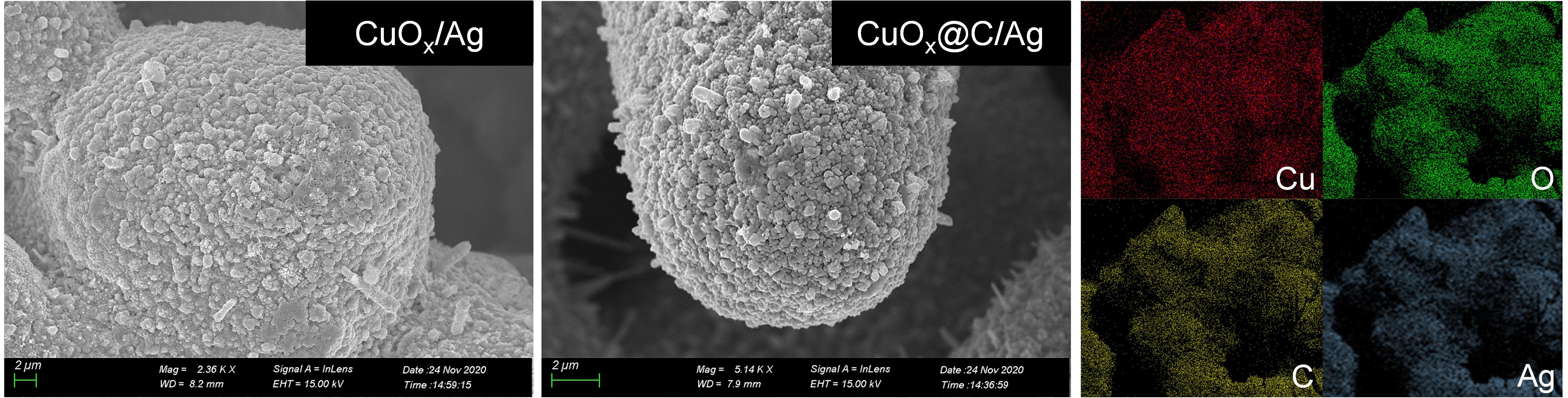


**Figure S31.** The SEM and EDX-mapping images of CuO_x_/Ag and CuO_x_@C/Ag.


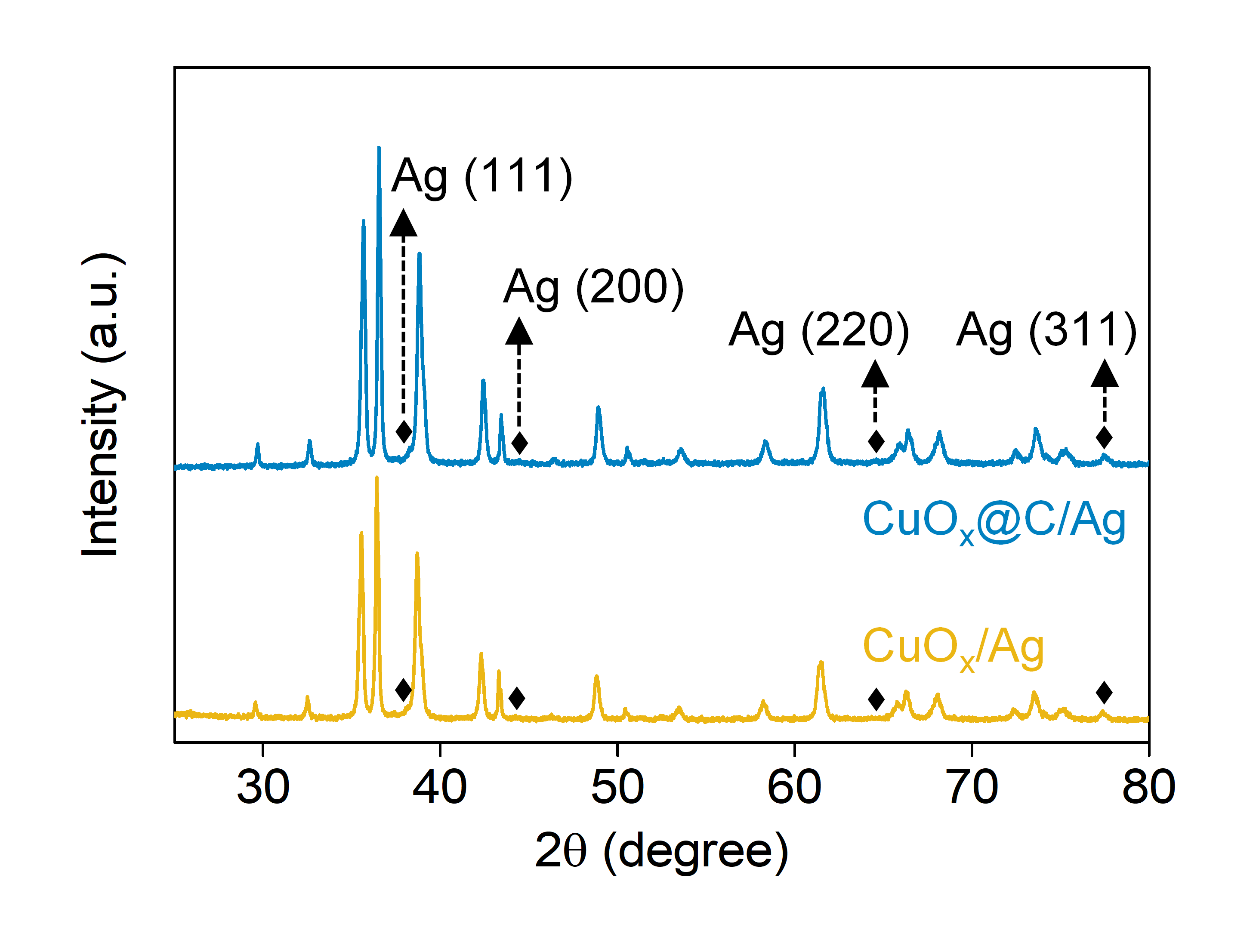


**Figure S32.** The XRD patterns of the CuO_x_/Ag and CuO_x_@C/Ag.


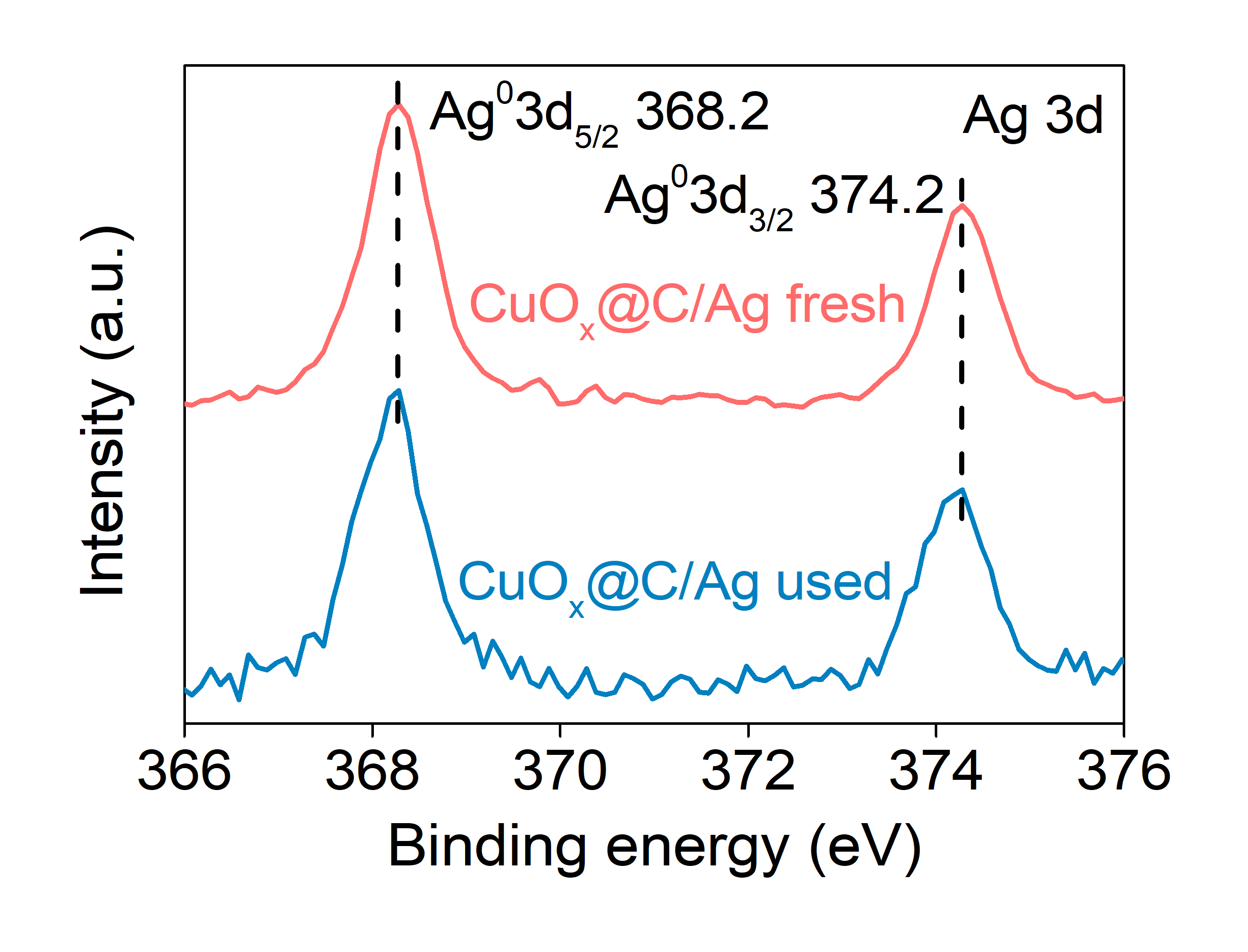


**Figure S33.** The XPS Ag 3d spectra of the fresh CuO_x_@C/Ag and used CuO_x_@C/Ag.


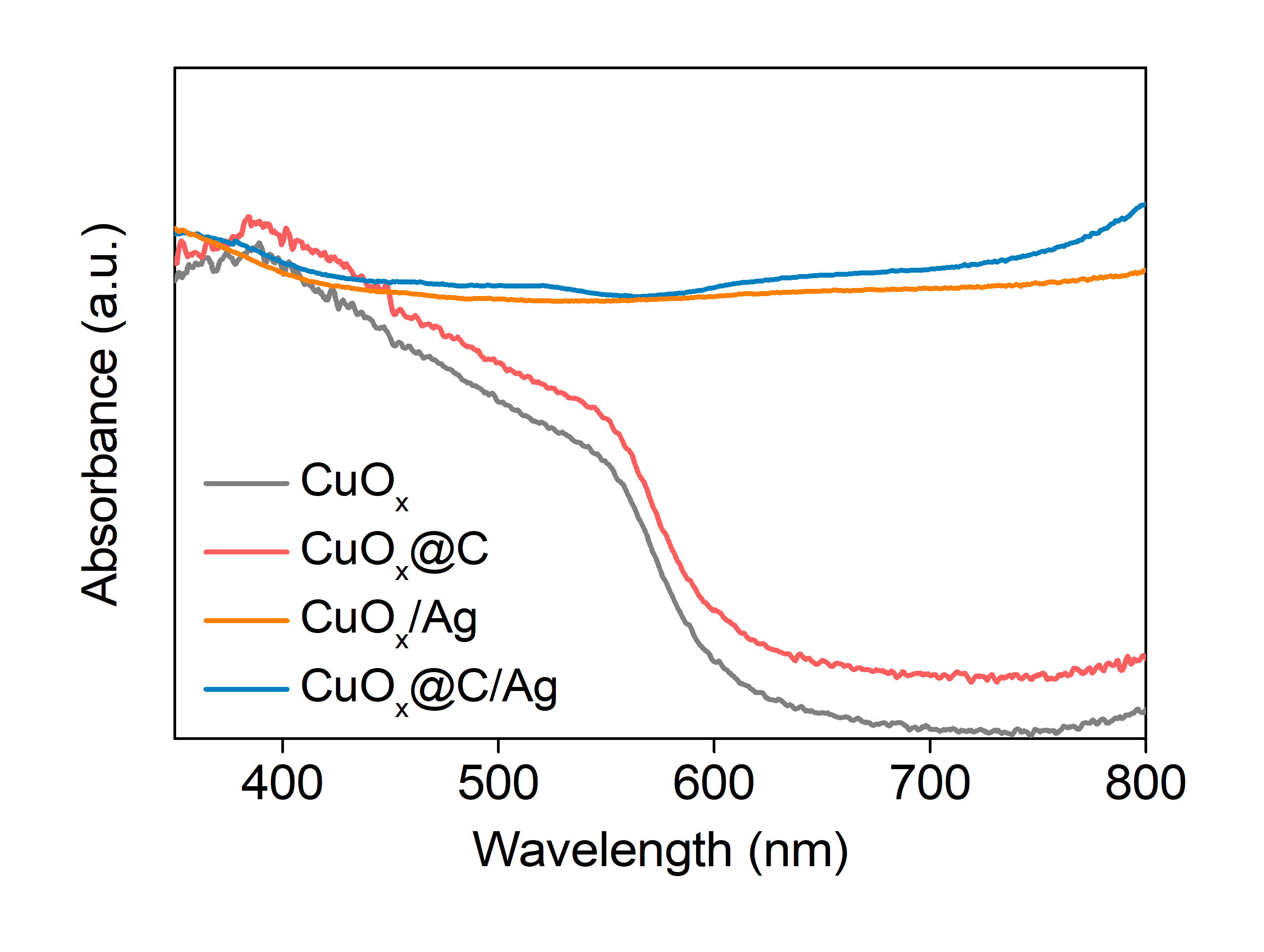


**Figure S34.** The DRS spectra of CuO_x_, CuO_x_@C, CuO_x_/Ag, and CuO_x_@C/Ag.


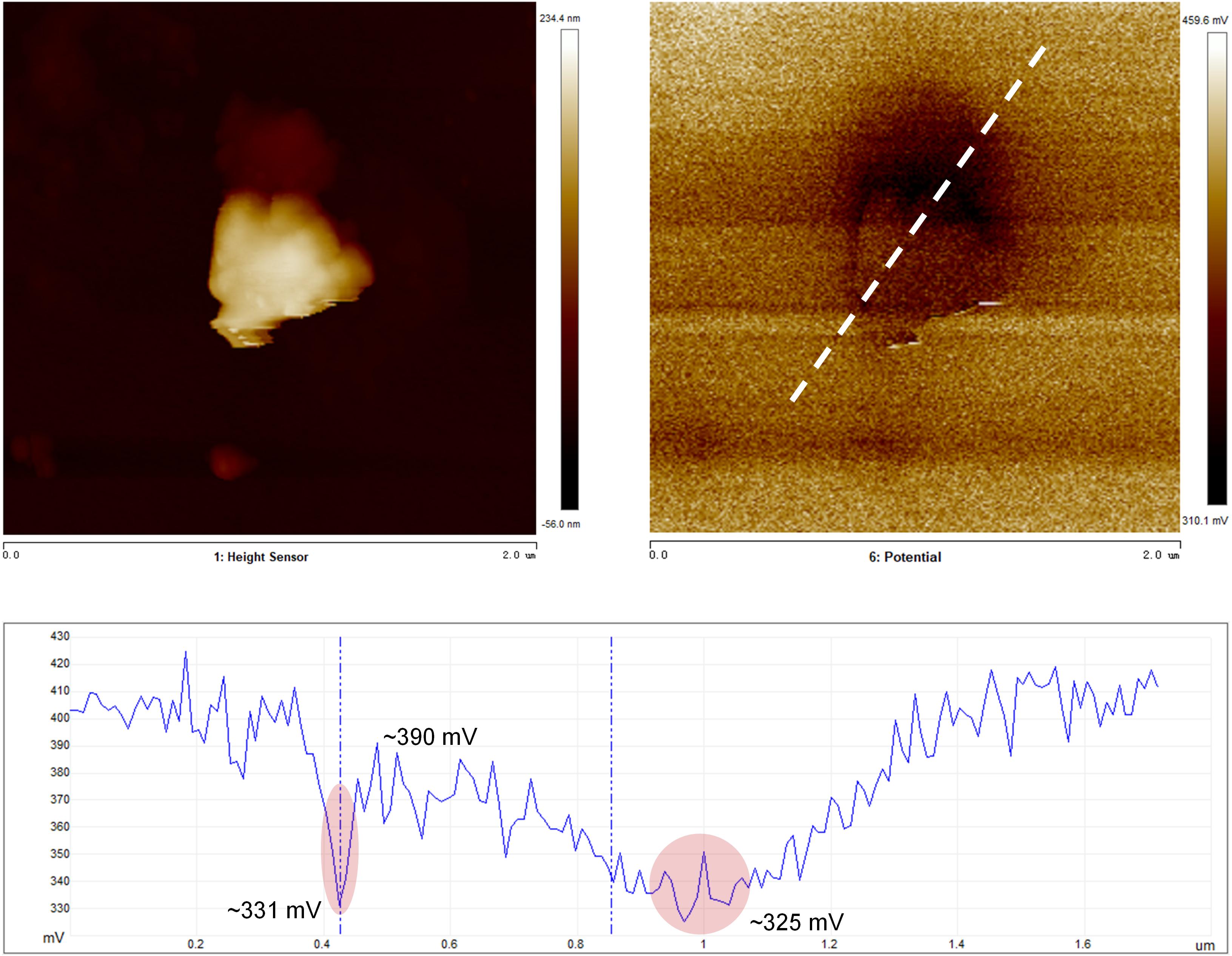


**Figure S35.** In-situ KPFM of CuO_x_@C/Ag under light irradiation.


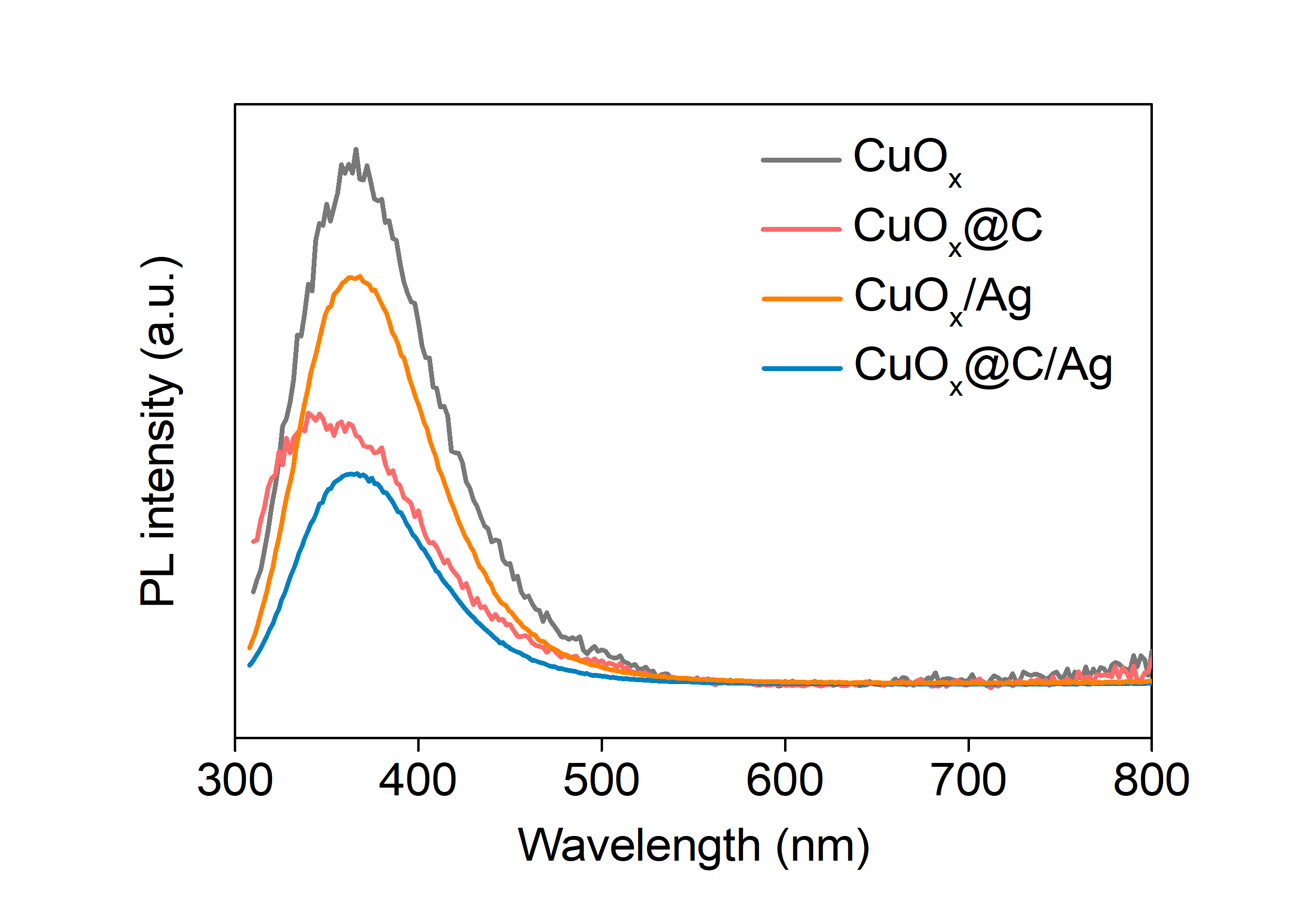


**Figure S36.** The steady-state PL spectra of CuO_x_, CuO_x_@C, CuO_x_/Ag, and CuO_x_@C/Ag.


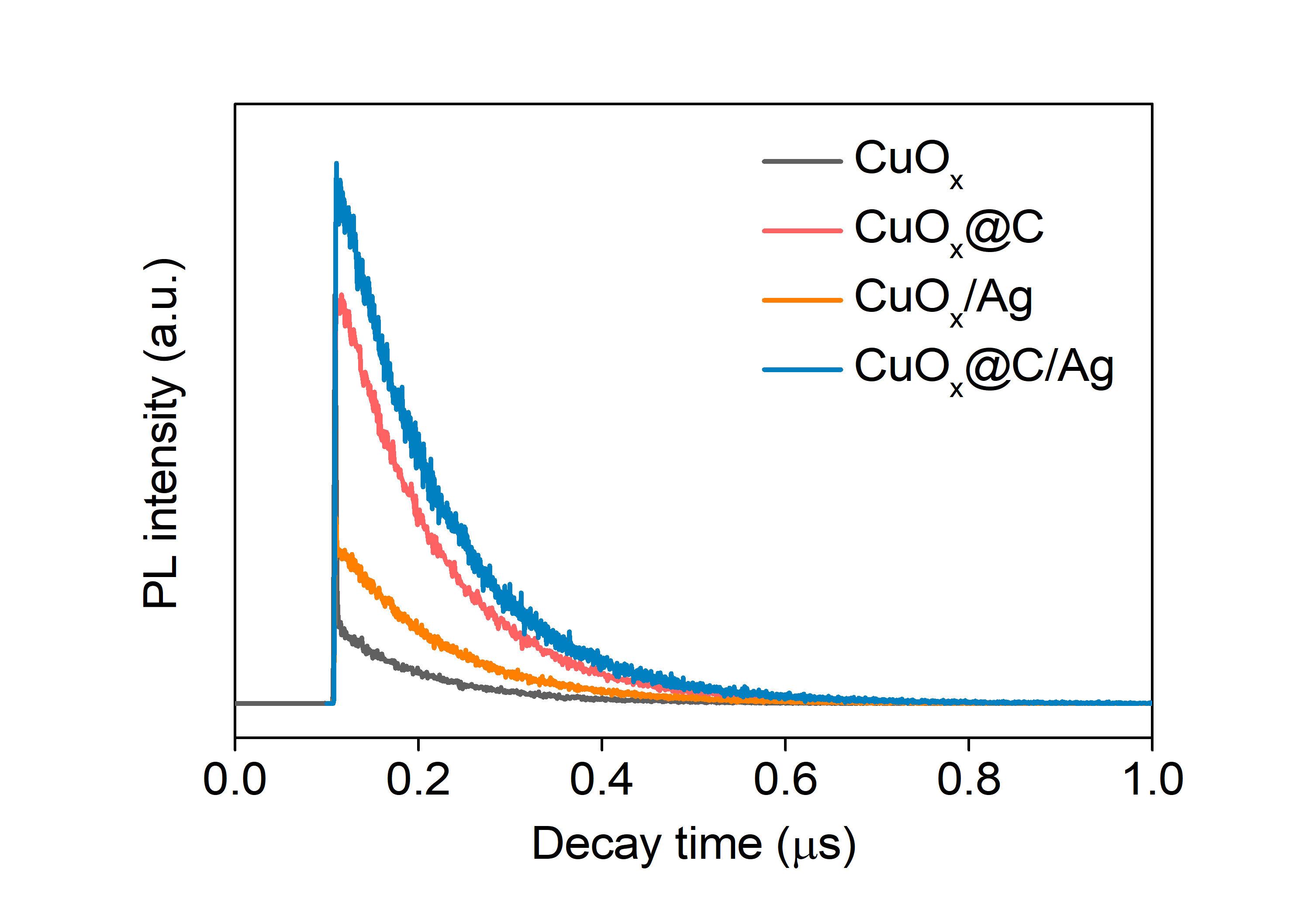


**Figure S37.** The time-resolved transient PL spectra of CuO_x_, CuO_x_@C, CuO_x_/Ag, and CuO_x_@C/Ag.


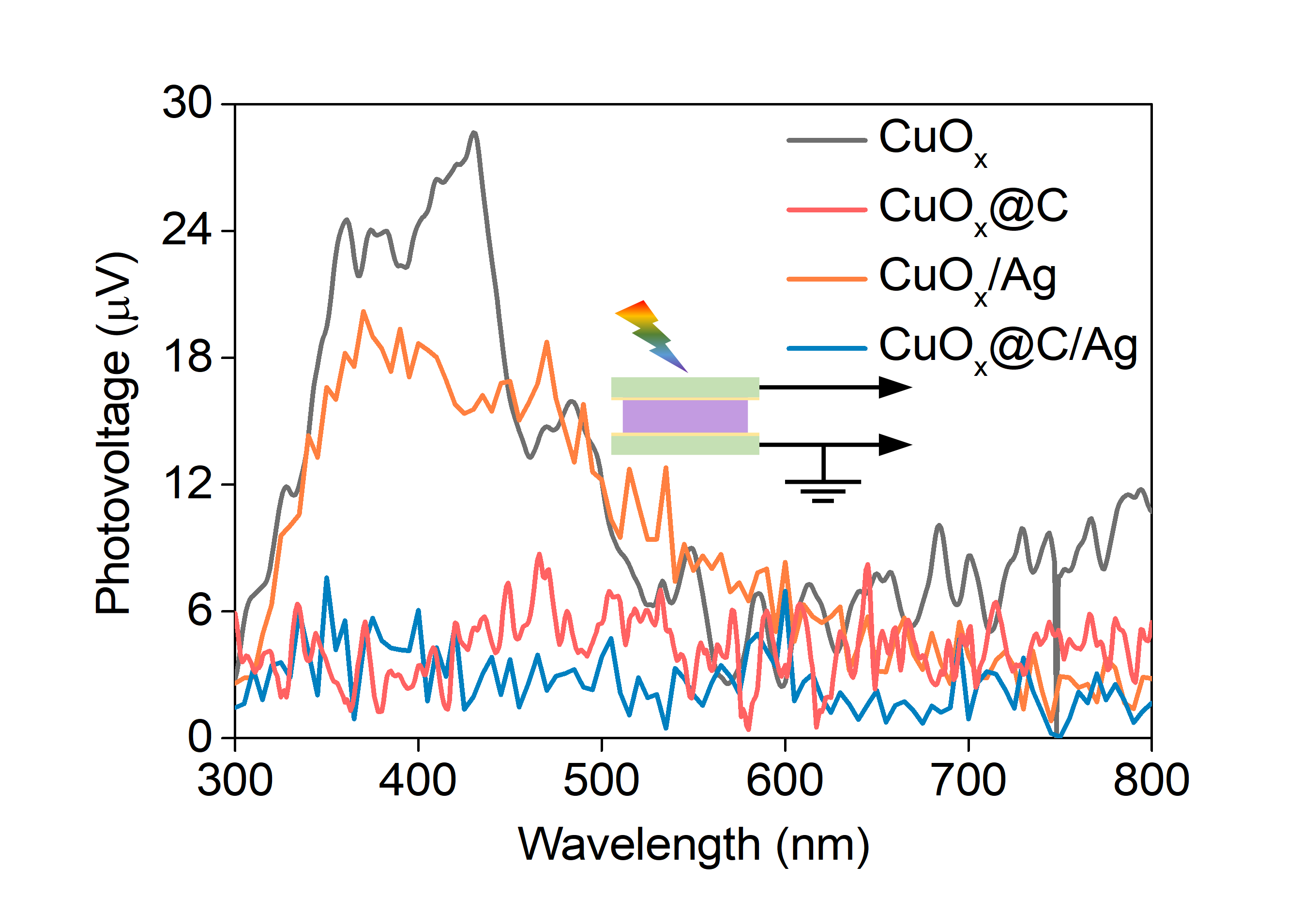


**Figure S38.** The SPV spectra of CuO_x_, CuO_x_@C, CuO_x_/Ag, and CuO_x_@C/Ag.


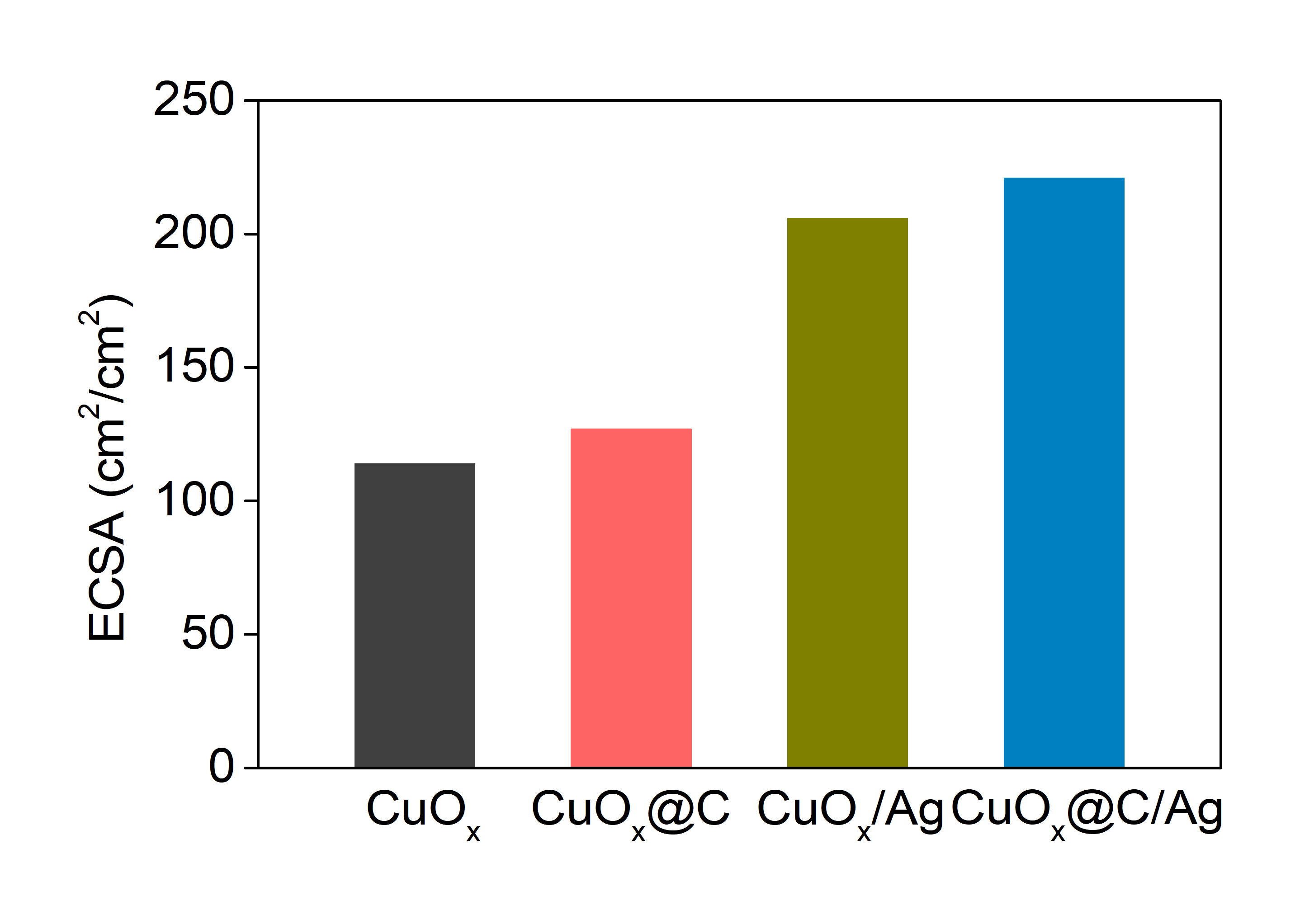


**Figure S39.** The electrochemical active surface area (ECSA) of CuO_x_, CuO_x_@C, CuO_x_/Ag, and CuO_x_@C/Ag. Experimental conditions: at −0.1 V vs. RHE under AM 1.5G simulated sunlight (100 mW/cm^2^) using 0.1 M KHCO_3_ as electrolyte (CO_2_-saturated).

As shown in **Figure S39**, both carbon-layer coating and silver modification markedly increase the electrochemical active surface area (ECSA) of the photoelectrode, with the latter exhibiting a substantially more pronounced effect. This enlargement of ECSA offers additional active sites for the subsequent CO_2_ reduction toward ethylene. The superior efficacy of silver modification is attributed to the high specific surface area of Ag nanoparticles, which provide considerably more active sites compared to the relatively smooth carbon layer.


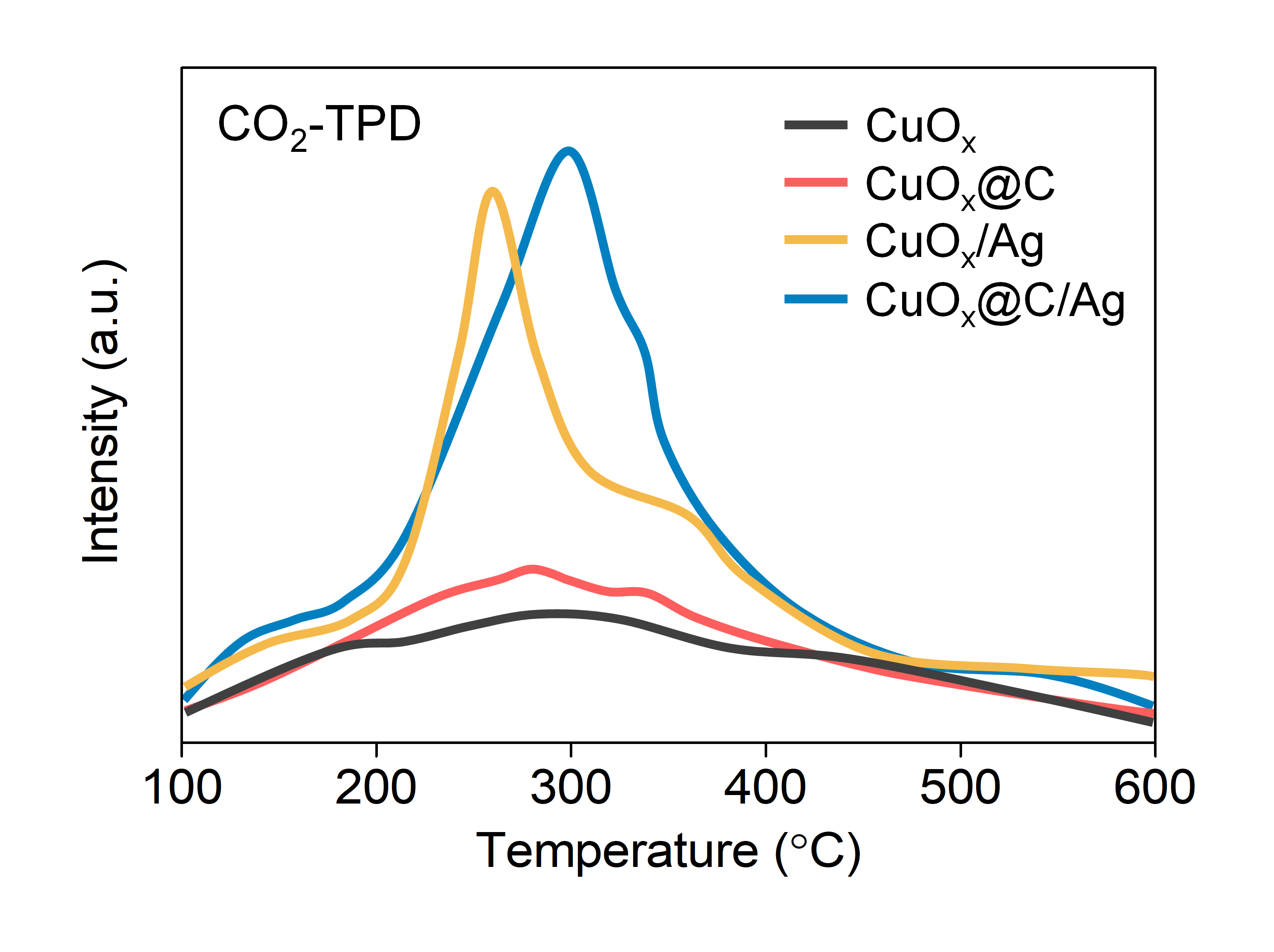


**Figure S40.** The CO_2_-TPD curves of CuO_x_, CuO_x_@C, CuO_x_/Ag, and CuO_x_@C/Ag.


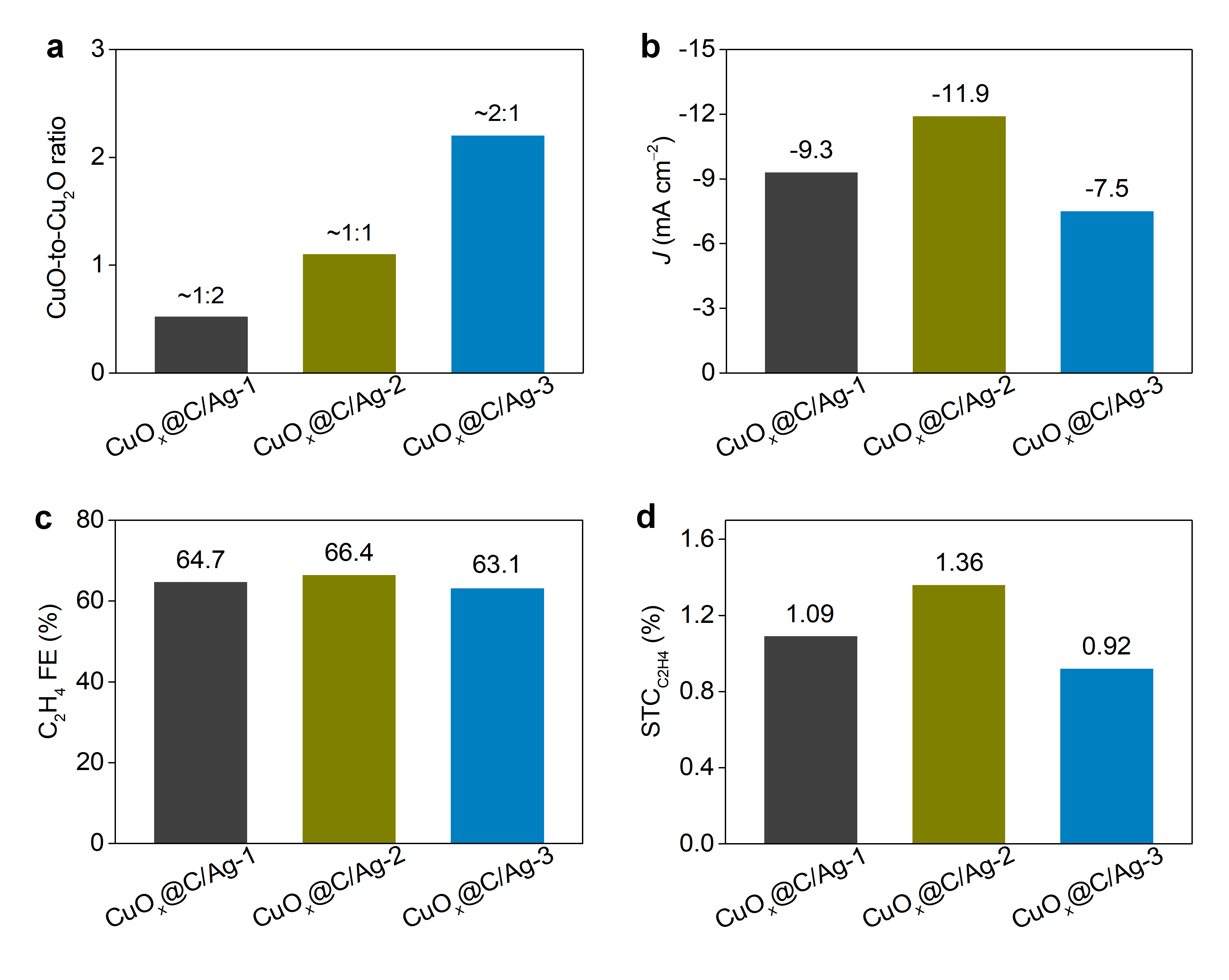


**Figure S41.** (a) The CuO/Cu_2_O ratio in three different CuO_x_@C/Ag photoelectrodes. (b) The photocurrent density of three different CuO_x_@C/Ag photoelectrodes. (c) The C_2_H_4_ FE on three different CuO_x_@C/Ag photoelectrodes. (d) The STC conversion efficiency for C_2_H_4_ production on three different CuO_x_@C/Ag photoelectrodes. Experimental conditions: at −0.1 V vs. RHE under AM 1.5G simulated sunlight (100 mW/cm^2^) using 0.1 M KHCO_3_ as electrolyte (CO_2_-saturated). Data are presented as mean ± s.d. (*n* = 3 independent chemical replicates) in d.

To investigate the effect of the CuO-to-Cu_2_O ratio on photoelectrocatalytic CO_2_ reduction to C_2_H_4_, three CuO_x_@C/Ag photoelectrodes with varying CuO/Cu_2_O ratios were fabricated by tuning the temperature and duration of the high-temperature calcination step during material synthesis. Specifically, the calcination conditions were set as follows: 300 °C for 2 h followed by 500 °C for 0.5 h; 300 °C for 2 h followed by 500 °C for 1 h; and 300 °C for 1 h followed by 500 °C for 2 h. The resulting photoelectrodes are denoted as CuO_x_@C/Ag-1, CuO_x_@C/Ag-2, and CuO_x_@C/Ag-3, respectively. Quantitative analysis of the Cu^2+^/Cu^+^ ratios was performed using XPS, confirming that the CuO-to-Cu_2_O ratios in CuO_x_@C/Ag-1, CuO_x_@C/Ag-2, and CuO_x_@C/Ag-3 were approximately ~1:2, ~1:1, and ~2:1, respectively (**Figure S41a**). Photocurrent density measurements revealed that CuO_x_@C/Ag-2, with a ~1:1 CuO-to-Cu_2_O ratio, exhibited the highest photocurrent density (~−11.9 mA cm⁻²), which is 1.3 and 1.6 times that of CuO_x_@C/Ag-1 and CuO_x_@C/Ag-3, respectively (**Figure S41b**). This result indicates that CuO_x_@C/Ag-2 possesses superior photogenerated charge carrier migration and separation efficiency, thereby affording a greater number of available electrons for CO_2_ reduction. Consistently, CuO_x_@C/Ag-2 demonstrated the highest photoelectrocatalytic activity for CO_2_ reduction to ethylene, achieving a Faradaic efficiency (FE) of ~66.4% for ethylene and a solar-to-chemical (STC) conversion efficiency of ~1.36%, both substantially outperforming those of CuO_x_@C/Ag-1 and CuO_x_@C/Ag-3 (**Figure S41c‒d**).


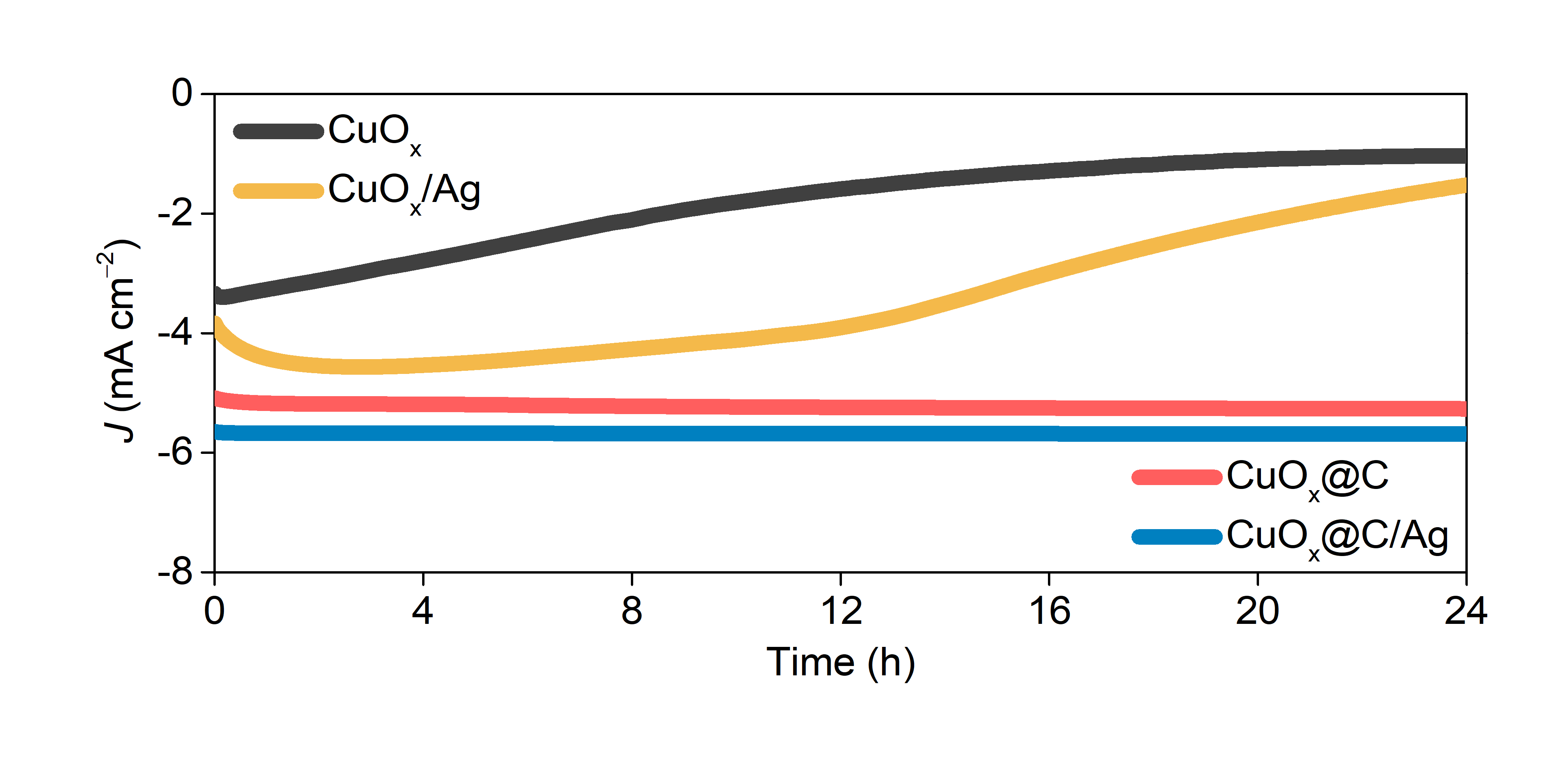


**Figure S42.** The *J*-*t* plots of the CuO_x_, CuO_x_@C, CuO_x_/Ag, and CuO_x_@C/Ag for photoelectrocatalytic CO_2_ reduction to C_2_H_4_. Experimental conditions: at −0.1 V vs. RHE under AM 1.5G simulated sunlight (100 mW/cm^2^) using 0.1 M KHCO_3_ as electrolyte (CO_2_-saturated).


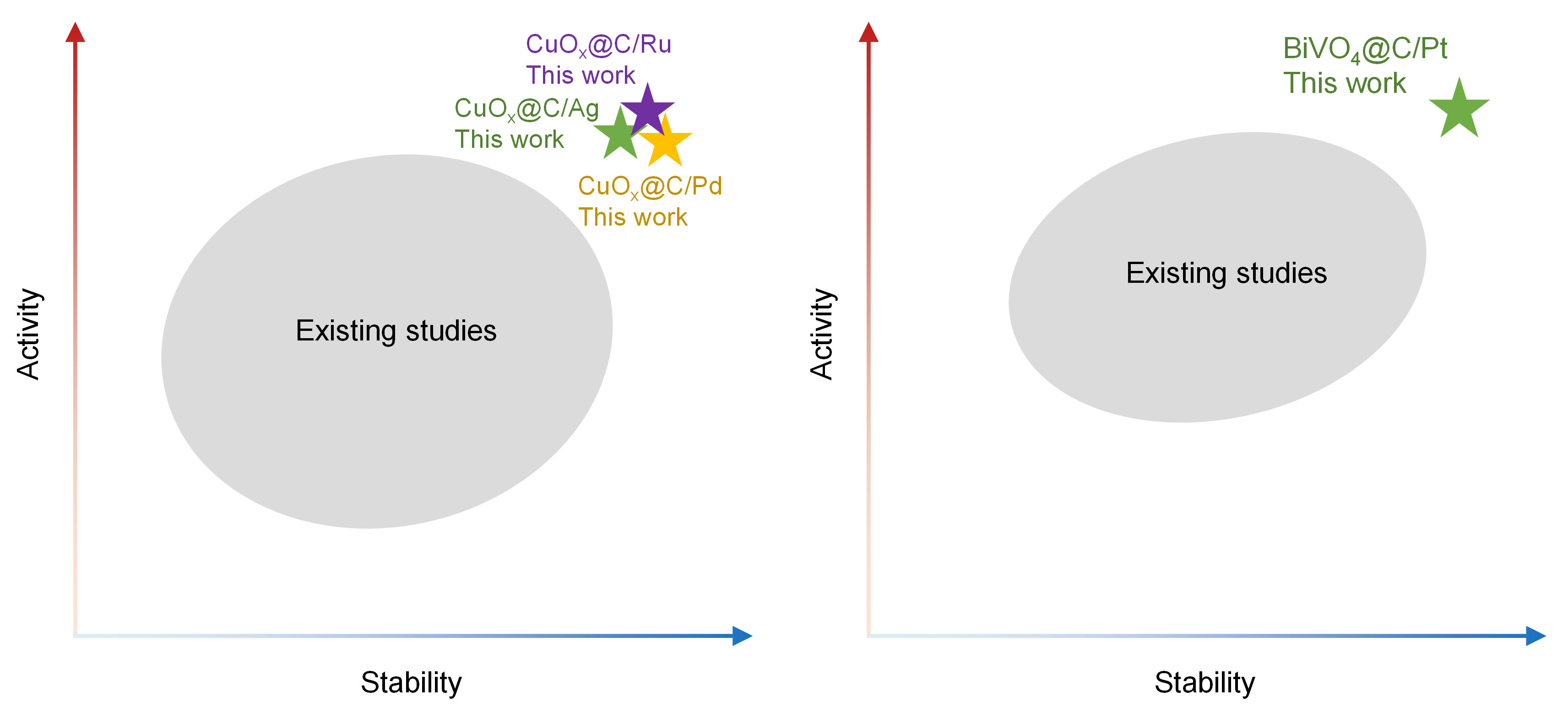


**Figure S43.** The comparison of activity and stability between this work with existing reports.


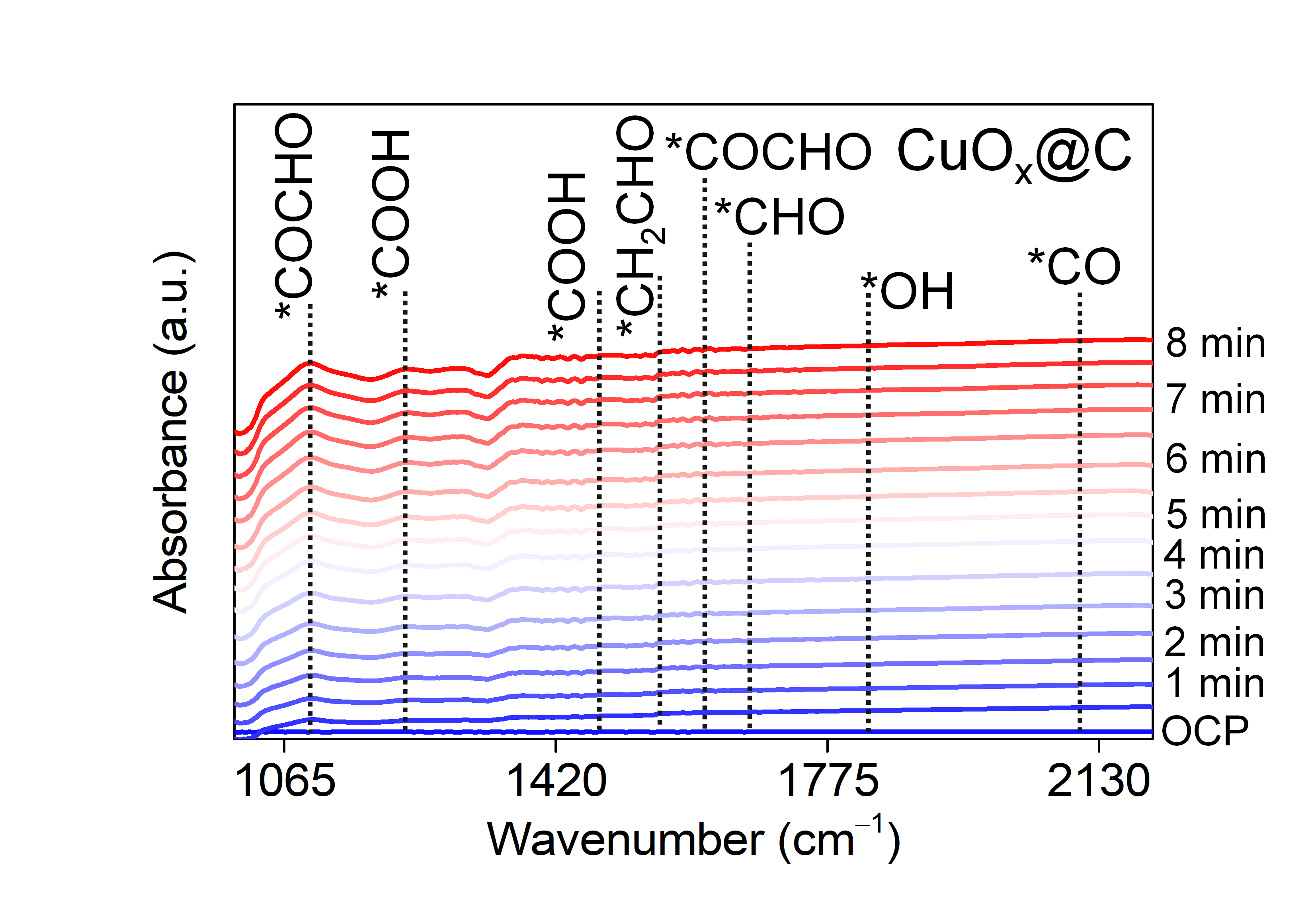


**Figure S44.** The ATR-FTIR of CuO_x_@C for photoelectrocatalytic CO_2_ reduction to C_2_H_4_. Experimental conditions: at −0.1 V vs. RHE under AM 1.5G simulated sunlight (100 mW/cm^2^) using 0.1 M KHCO_3_ as electrolyte (CO_2_-saturated).


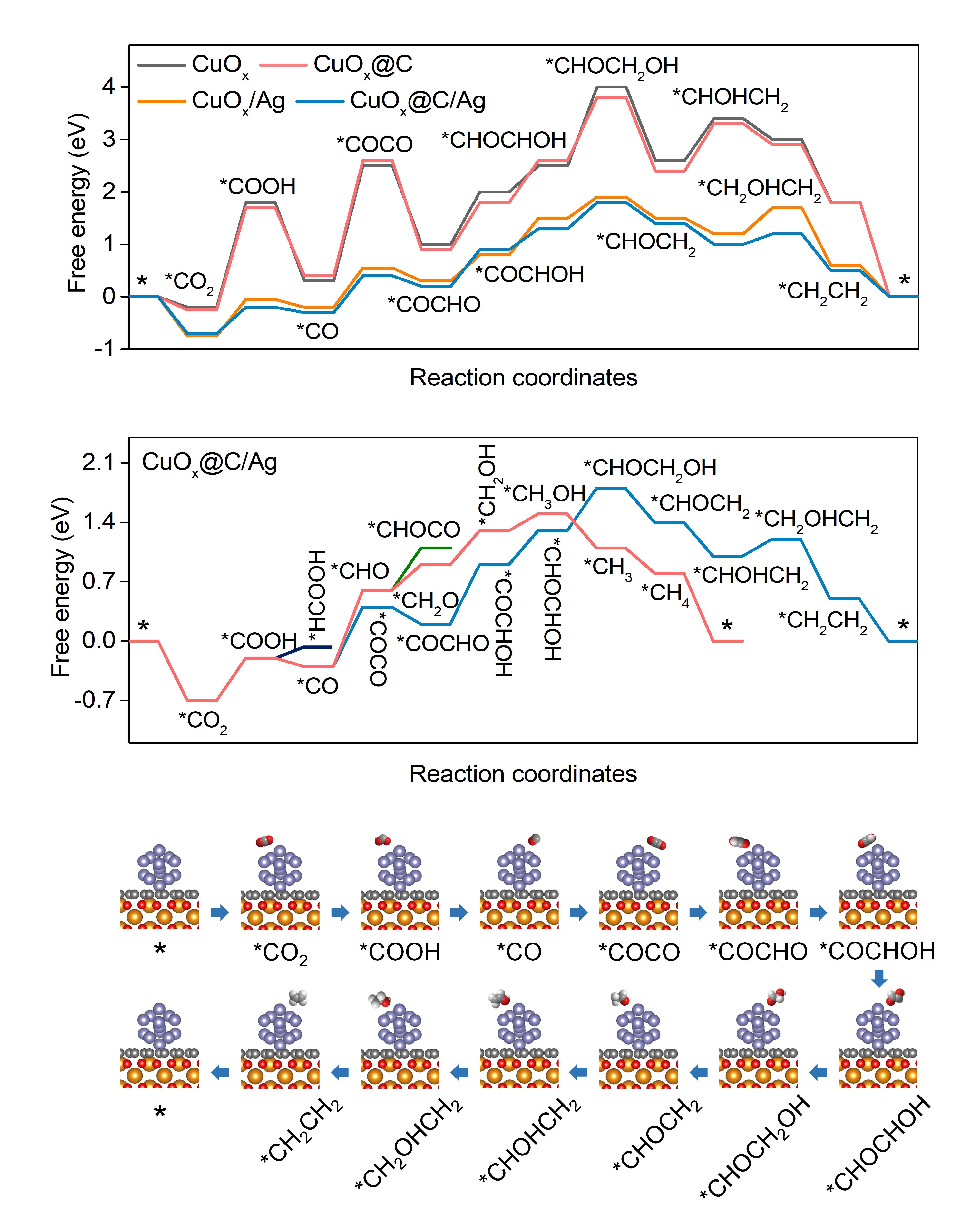


**Figure S45.** The reaction pathways and free energy changes (∆*G*) of CO_2_ reduction on CuO_x_, CuO_x_@C, CuO_x_/Ag, and CuO_x_@C/Ag.

**Table S1.** The comparison of activity and stability between this work with existing reports.

| No. | Photoelectrode | Reaction | Activity | Stability | References |
| --- | --- | --- | --- | --- | --- |
| 1 | Cu_2_O | CO_2_ reduction to CH_3_OH | Faradaic efficiency = ~72% | Stable operation for ~1 h | Nat. Energy 4, 957-968 (2019) |
| 2 | Cu_2_O | CO_2_ reduction to CO/CH_4_/CH_3_OH | Faradaic efficiency = ~85% | Stable operation for ~3 h | Angew. Chem. Int. Ed. 2016, 55, 8840-8845 |
| 3 | CuO_x_ | CO_2_ reduction to C_2_H_4_/C_2_H_5_OH | Faradaic efficiency = ~34% | Stable operation for ~3 h | J. Am. Chem. Soc. 2025, 17, 14, 13974-13984 |
| 4 | Ce-Cu_2_O | CO_2_ reduction to C_2_H_4_ | Faradaic efficiency = ~25% | Stable operation for ~8 h | ACS Nano 2023, 17, 14, 13974-13984 |
| 5 | CuO/Ni | CO_2_ reduction to C_2_H_4_ | Faradaic efficiency = ~54.1% | Stable operation for ~3 h | Sci. Bull. 67 (2022) 1679-1687 |
| 6 | CuO/NCDs | CO_2_ reduction to C_2_H_4_ | Faradaic efficiency = ~56% | Stable operation for ~6 h | Adv. Funct. Mater. 2022, 32, 2113335 |
| 7 | Cu_2_O/TiO_2_ | CO_2_ reduction to CO | Faradaic efficiency = ~80% | Stable operation for ~1.5 h | J. Am. Chem. Soc. 2016, 138, 6, 1938-1946 |
| 8 | Cu_2_O/SnO_x_ | CO_2_ reduction to CO | Faradaic efficiency = ~90% | Stable operation for ~12 h | Adv. Funct. Mater. 2022, 32, 2109600 |
| 9 | Cu_2_O@Cu_2_S | CO_2_ reduction to C_2_H_5_OH | Faradaic efficiency = ~43.9% | Stable operation for ~14 h | ACS Catal. 2024, 14, 5, 3266-3277 |
| 10 | Si/ZnO/Cu_2_O | CO_2_ reduction to C_2_H_5_OH | Faradaic efficiency = ~60% | Stable operation for ~2 h | Adv. Energy Mater. 2022, 12, 2201134 |
| 11 | Cu_2_O@Cu_3_(BTC)_2_ | CO_2_ reduction to CH_4_ | Faradaic efficiency = ~80% | Stable operation for ~8 h | Angew. Chem. Int. Ed. 2021, 60, 8455-8459 |
| 12 | Ag/Cu_2_O/ZS | CO_2_ reduction to C_2_H_4_ | Faradaic efficiency = ~60% | Stable operation for ~6 h | Nat. Energy 6, 1124-1132 (2021) |
| 13 | Cu_3_(BTC)_2_/Cu_2_O | CO_2_ reduction to CO | Faradaic efficiency = ~95% | Stable operation for ~1 h | J. Am. Chem. Soc. 2019, 141, 27, 10924-10929 |
| 14 | Cu_2_O/Ga_2_O_3_/TiO_2_ | CO_2_ reduction to HCOOH | Faradaic efficiency = ~38% | Stable operation for ~4 h | J. Am. Chem. Soc. 2023, 145, 51, 27939-27949 |
| **15** | **CuO_x_@C/Ag** | **CO_2_ reduction to C_2_H_4_** | **Faradaic efficiency = ~66.4%** | **Stable operation for >24 h** | **This work** |
| 16 | Cu_2_O | Nitrate reduction to NH_3_ | Faradaic efficiency = ~85% | Stable operation for ~5 h | ACS Energy Lett. 2024, 9, 5, 1993-1999 |
| 17 | Cu_2_O | Nitrate reduction to NH_3_ | Faradaic efficiency = ~79% | Stable operation for ~2 h | Nat. Mater. (2025) |
| 18 | Cu_2_O-Bi | Nitrate reduction to NH_3_ | Faradaic efficiency = ~95% | Stable operation for ~2.5 h | Mater. Today 76, 2024, 52-63 |
| 19 | CuO_x_ | Nitrate reduction to NH_3_ | Faradaic efficiency = ~81% | Stable operation for ~9 h | Nat. Commun. 14, 7383 (2023) |
| 20 | CuO_x_/GDY | Nitrate reduction to NH_3_ | Faradaic efficiency = ~96% | Stable operation for ~10 h | Adv. Mater. 2024, 36, 2405660 |
| **21** | **CuO_x_@C/Ru** | **Nitrate reduction to NH_3_** | **Faradaic efficiency = ~97.5%** | **Stable operation for >24 h** | **This work** |
| 22 | CuO_x_/Pd | Dechlorination of trichloroacetic acid | Dechlorination rate = ~89% | Stable operation for ~10 h | Environ. Sci. Technol. 2019, 53, 24, 14586-14594 |
| 23 | Cu_2_O/Pd | Dechlorination of polychlorinated biphenyls | Dechlorination rate = ~95% | Stable operation for ~10 h | J. Am. Chem. Soc. 2014, 136, 1, 32-35 |
| 24 | CuO_x_/Pd | Dechlorination of p-chloroaniline | Dechlorination rate = ~96% | Stable operation for ~10 h | Environ. Sci. Technol. 2021, 55, 5, 3296-3304 |
| 25 | CuO_x_/Pd | Dechlorination of p-chlorophenol | Dechlorination rate = ~96% | Stable operation for ~12 h | Appl. Catal. B Environ. Energy 343 (2024) 123554 |
| **26** | **CuO_x_@C/Pd** | **Dechlorination of p-chlorophenol** | **Dechlorination rate = ~99.2%** | **Stable operation for >24 h** | **This work** |
| 27 | BiVO_4_ | Oxygen evolution reaction | Photocurrent  = ~2.1 mA/cm^2^ | Stable operation for ~16 h | J. Am. Chem. Soc. 2022, 144, 50, 23073-23080 |
| 28 | BiVO_4_ | Oxygen evolution reaction | Photocurrent  = ~2.3 mA/cm^2^ | Stable operation for ~18 h | ACS Energy Lett. 2021, 6, 10, 3400-3407 |
| 29 | BiVO_4_/FeOOH | Oxygen evolution reaction | Photocurrent  = ~3 mA/cm^2^ | Stable operation for ~30 h | Nat. Energy 3, 53-60 (2018) |
| 30 | BiVO_4_/FeOOH | Oxygen evolution reaction | Photocurrent  = ~2.8 mA/cm^2^ | Stable operation for ~18 h | J. Am. Chem. Soc. 2023, 145, 43, 23639-23650 |
| 31 | BiVO_4_/NiFeMOFs | Oxygen evolution reaction | Photocurrent  = ~2.7 mA/cm^2^ | Stable operation for ~10 h | Angew. Chem. Int. Ed. 2021, 60, 1433-1440 |
| **32** | **BiVO_4_@C/Pt** | **Oxygen evolution reaction** | **Photocurrent**  **= ~3 mA/cm^2^** | **Stable operation for >40 h** | **This work** |

**References**

1 Zhang, K. et al. Near-complete suppression of oxygen evolution for photoelectrochemical H_2_O oxidative H_2_O_2_ synthesis. *J. Am. Chem. Soc.* **142**, 8641-8648 (2020).

2 Ye, K.-H. et al. Enhancing photoelectrochemical water splitting by combining work function tuning and heterojunction engineering. *Nat. Commun.* **10**, 3687 (2019).

3 Wu, H. et al. Low-bias photoelectrochemical water splitting via mediating trap states and small polaron hopping. *Nat. Commun.* **13**, 6231 (2022).

4 Kim, J. et al. Vitamin C-induced CO_2_ capture enables high-rate ethylene production in CO_2_ electroreduction. *Nat. Commun.* **15**, 192 (2024).

5 Yao, Y. et al. A surface strategy boosting the ethylene selectivity for CO_2_ reduction and in situ mechanistic insights. *Nat. Commun.* **15**, 1257 (2024).

6 Zhan, C. et al. Key intermediates and Cu active sites for CO_2_ electroreduction to ethylene and ethanol. *Nat. Energy* **9**, 1485-1496 (2024).

7 Chen, Z. H. et al. Precision Molecular Engineering of Carbon Nitride for Efficient and Selective Photoreduction of CO_2_ to C_2_H_6_ in Pure Water. *Adv. Funct. Mater.* **35**, 202423213 (2025).

8 Tayyebi, A. et al. Bias-free solar NH_3_ production by perovskite-based photocathode coupled to valorization of glycerol. *Nat. Catal.* **7**, 510-521 (2024).

9 Dai, J. et al. Spin polarized Fe1-Ti pairs for highly efficient electroreduction nitrate to ammonia. *Nat. Commun.* **15**, 88 (2024).

10 Gao, J. et al. Coupling curvature and hydrophobicity: A counterintuitive strategy for efficient electroreduction of nitrate into ammonia. *ACS Nano* **18**, 10302-10311 (2024).

11 Cheon, S. et al. Neighboring catalytic sites are essential for electrochemical dechlorination of 2-chlorophenol. *J. Am. Chem. Soc.* **146**, 25151-25157 (2024).

12 Zhang, J. et al. Synchronous reduction-oxidation process for efficient removal of trichloroacetic acid: H* initiates dechlorination and •OH is responsible for removal efficiency. *Environ. Sci. Technol.* **53**, 14586-14594 (2019).

13 Xiao, Q., Li, W., Xie, S., Wang, L. & Tang, C. Y. Ultrafast complete dechlorination enabled by ferrous oxide/graphene oxide catalytic membranes via nanoconfinement advanced reduction. *Nat. Commun.* **15**, 9607 (2024).

14 Zhao, Y. et al. Gold Single Atom Doped Defective Nanoporous Copper Octahedrons for Electrocatalytic Reduction of Carbon Dioxide to Ethylene. *ACS Nano* **19**, 4505-4514, (2025).

15 Yao, Y. C. et al. Single Atom Ru Monolithic Electrode for Efficient Chlorine Evolution and Nitrate Reduction. *Angew. Chem. Int. Ed.* **61**, 202208215 (2022).

16 Lin, Y. X. et al. Optimizing Local Configuration of Interphase Copper Oxide by Ru Atoms Incorporation for High-Efficient Nitrate Reduction to Ammonia. *Adv. Funct. Mater.* **35**, 202417486 (2025).

17 Yang, M. Q. et al. Highly Selective Electrochemical Nitrate to Ammonia Conversion by Dispersed Ru in a Multielement Alloy Catalyst. *Nano Lett.* **23**, 7733-7742, (2023).

18 Chen, F. Y. et al. Efficient conversion of low-concentration nitrate sources into ammonia on a Ru-dispersed Cu nanowire electrocatalyst. *Nat. Nanotechnol.* **17**, 759-767, (2022).

19 Wu, Y. F. et al. Enhanced electrocatalytic dechlorination of para-chloronitrobenzene based on Ni/Pd foam electrode. *Chem. Eng. J.* **316**, 146-153, (2017).

20 Mao, Z. C. et al. Atomically dispersed Pd electrocatalyst for efficient aqueous phase dechlorination reaction. *Electrochim. Acta* **391**, 138886 (2021).

21 Wu, C. Y. et al. Dechlorination of 2,4-dichlorophenol in a hydrogen-based membrane palladium-film reactor: Performance, mechanisms, and model development. *Water Res.* **188**, 116465 (2021).

22 Yu, W. T., Jiang, H., Fang, J. H. & Song, S. Designing an Electron-Deficient Pd/NiCoO Bifunctional Electrocatalyst with an Enhanced Hydrodechlorination Activity to Reduce the Consumption of Pd. *Environ. Sci. Technol.* **55**, 10087-10096, (2021).
